# Health impact and cost-effectiveness of COVID-19 booster vaccination strategies in the early post-Omicron era: a dynamic modelling study

**DOI:** 10.1136/bmjgh-2024-016096

**Published:** 2025-09-29

**Authors:** Thao P Le, Eamon Conway, Edifofon Akpan, Isobel R Abell, Patrick Abraham, Christopher M Baker, Patricia T Campbell, Deborah Cromer, Michael J Lydeamore, Yasmine McDonough, Ivo Mueller, Gerard Ryan, Camelia Walker, Cyan Yingying Wang, Natalie Carvalho, Jodie McVernon

**Affiliations:** 1 School of Mathematics and Statistics, The University of Melbourne, Melbourne, Victoria, Australia; 2 Melbourne Centre for Data Science, School of Mathematics and Statistics, The University of Melbourne, Melbourne, Victoria, Australia; 3 Centre of Excellence for Biosecurity Risk Analysis, The University of Melbourne, Melbourne, Victoria, Australia; 4 Population Health & Immunity Division, Walter and Eliza Hall Institute of Medical Research, Melbourne, Victoria, Australia; 5 Melbourne School of Population and Global Health, The University of Melbourne, Melbourne, Victoria, Australia; 6 Department of Infectious Diseases at The Peter Doherty Institute for Infection and Immunity, The University of Melbourne, Melbourne, Victoria, Australia; 7 Kirby Institute, University of New South Wales, Sydney, New South Wales, Australia; 8 Department of Econometrics and Business Statistics, Monash University, Melbourne, Victoria, Australia; 9 Department of Medical Biology, The University of Melbourne, Melbourne, Victoria, Australia; 10 The Kids Research Institute Australia, Nedlands, Western Australia, Australia; 11 Victorian Infectious Diseases Reference Laboratory Epidemiology Unit, The Royal Melbourne Hospital at the Peter Doherty Institute for Infection and Immunity, Melbourne, Victoria, Australia

**Keywords:** COVID-19, Mathematical modelling, Health economics

## Abstract

**Introduction:**

Following widespread exposure to Omicron variants, SARS-CoV-2 has transitioned to endemic circulation. Populations now have diverse infection and vaccination histories, resulting in heterogeneous immune landscapes. Careful consideration of the value of ongoing vaccination is required through the post-Omicron phase of COVID-19 management to minimise disease burden. We demonstrate the utility of a modelling approach to address this question, supporting recommendations for targeted vaccine use across different country settings.

**Methods:**

We integrated immunological, transmission, clinical and cost-effectiveness models and simulated populations with different characteristics and immune landscapes over the early post-Omicron period. We calculated the expected number of infections, hospitalisations and deaths for different vaccine scenarios. Costs (from a healthcare perspective) were estimated for exemplar country income-level groupings in the Western Pacific Region using pandemic-era vaccine prices and healthcare-seeking behaviour assumptions. We assessed the impact and cost-effectiveness of targeted vaccination strategies. Results are reported as incremental costs and disability-adjusted life years averted compared with no additional vaccination. Parameter and stochastic uncertainty were captured through scenario and sensitivity analysis.

**Results:**

Across different population demographics and income levels, we consistently found that annual elder-targeted boosting strategies are most likely to be cost-effective or cost-saving (>75% probability of being cost-effective among older, high-income settings; >50% probability of being cost-effective in younger, middle-income settings), while paediatric programmes are unlikely to be cost-effective. Results remained broadly consistent while accounting for uncertainties in the epidemiological and economic models, although they were sensitive to the cost of home-based care and vaccination. Use of pandemic-era vaccine prices may underestimate current vaccine prices available in upper-middle-income and high-income settings, potentially overestimating the cost-effectiveness of boosting in these settings. Half-yearly boosting may only be cost-effective in higher income settings with older population demographics and higher cost-effectiveness thresholds.

**Conclusion:**

Competing health priorities and resource constraints mean COVID-19 vaccine allocation needs to be carefully considered in context. These results, reflecting modelling conducted on the early post-Omicron period, demonstrate the value of continued booster vaccinations to protect against severe COVID-19 disease outcomes across high-income and middle-income settings and show that the biggest health gains relative to vaccine costs are achieved by targeting older age groups.

**WHAT IS ALREADY KNOWN ON THIS TOPIC**

- COVID-19 is now globally endemic and populations exhibit varying levels of natural and vaccine-acquired immunity to SARS-CoV-2.
- Consensus is that allocating vaccine doses to older age groups and those at higher risk of severe disease is most beneficial, although past studies typically assume either only past natural immunity or no waning immunity.
- COVID-19 vaccination strategies must consider the cost-effectiveness of gains from vaccination given prior immunity, and in the context of income and health system capacity to manage COVID-19 alongside other pressing concerns.

**WHAT THIS STUDY ADDS**

- This study considers multiple population demographics with varying degrees of hybrid immunity resulting from both prior infection and vaccination, with protection that wanes over time.
- COVID-19 booster doses targeted towards older age groups at risk of severe outcomes can be cost-effective or cost-saving in high-income settings with populations that have a higher proportion of individuals at risk.
- In younger, lower-resourced settings, annual boosting of older age groups may still be cost-effective or cost-saving in some scenarios.
- Paediatric vaccination is consistently found to be not cost-effective.

**HOW THIS STUDY MIGHT AFFECT RESEARCH, PRACTICE OR POLICY**

- Findings emphasise the importance of ongoing COVID-19 vaccination, especially to reduce severe disease.
- The results give evidence to support vaccination recommendations and ongoing global efforts to provide and equitably distribute vaccines and strengthen adult immunisation programmes.

## Introduction

 Since the emergence of SARS-CoV-2 in late 2019, the world has experienced multiple epidemic waves of COVID-19 disease and diverse evolutionary variants of SARS-CoV-2, especially Omicron variant B.1.1.529 and its many subvariants (BA.1, BA.2, BA.3, BA.4, BA.5, XBB, recombinants and further descendent lineages).^1^ In parallel to the evolution of the virus, there have been multiple rounds of vaccination around the world, administering a range of COVID-19 vaccines, from those based on the ancestral variant to contemporary Omicron-variant-adjusted vaccines and bivalent vaccines.^2^

Both prior infection and vaccination can reduce the chance of future infection and severity of outcomes, combining to form ‘hybrid immunity’^3^ against COVID-19. In the post-Omicron era (starting late 2021), most populations have high levels of past infection across multiple epidemic waves, creating exposure-derived ‘natural’ immunity. Vaccine coverage has been variable due to inequities of access, eligibility and uptake, with consequences for hybrid immunity landscapes.^4–6^ Unfortunately, all forms of immunity wane over time, enabling possible reinfection within a matter of months, though protection against severe outcomes is longer lived.^6 7^

How can COVID-19 vaccines incorporated into routine immunisation schedules help minimise the impact of recurring epidemic waves and promote resilience against future variants? Heterogeneous population experiences of infection and vaccination, along with the irregular emergence of immune escape variants, make it challenging to anticipate the timing, magnitude and clinical burden of future epidemics. The WHO recently reviewed evidence for the impact and cost-effectiveness of COVID-19 booster vaccine strategies. Key questions to inform strategic guidelines included: the incremental benefits of boosters, identification of optimal vaccine target groups in high seroprevalence settings, ongoing vaccine-preventable disease burden, optimum boosting strategy including frequency for priority populations and the cost effectiveness of those vaccination strategies. We need flexible frameworks to investigate this multidimensional problem space.

As one of several groups commissioned by WHO to support decision making, we adapted an existing model representing diverse population and hybrid immunity landscapes^5^ to address questions relevant to future COVID-19 vaccine prioritisation.^8^ We mimicked realistic epidemic exposure histories and specified timings for the emergence of immune escape variants. We also proposed plausible vaccine rollout and coverage assumptions. We developed exemplar demographies and costings based on high-middle and low-middle income countries in the Western Pacific Region.

Our findings played a role in informing the WHO updated COVID-19 vaccination guidance for March 2023.^9^

## Methods

**Figure 1:**
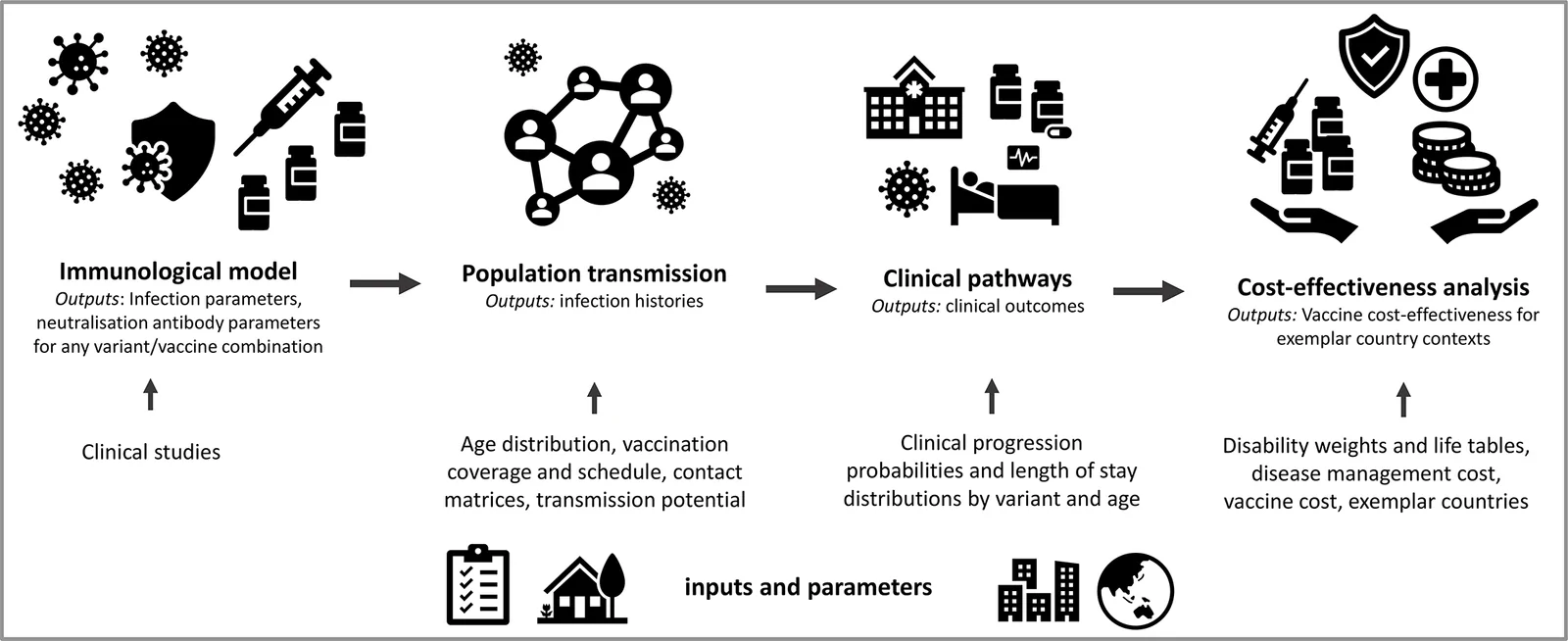
Simulation and analysis model framework from the immunological model to the cost-effectiveness analysis. The immunological model converts vaccinations and infections into neutralising antibody parameters, which influence outbreak dynamics and clinical outcomes. The simulation model has two parts: first, an agent-based model of population level transmission, with multiple primary inputs including vaccination and demography, followed by a clinical pathways model, which generates outcomes using the time-series of symptomatic infections, neutralising antibodies and age-dependent clinical progression probabilities. The resultant clinical outcomes are used in the cost-effectiveness analysis, along with other input parameters such as vaccine and disease management costs.

Our modelling pipeline is depicted in figure 1. First, the immunological model^10^ informs an infection transmission dynamics model within a mechanical agent-based model.^11^ The outputs of the agent-based model are input to a clinical pathways model to obtain clinical outcomes.^11^ These clinical outcomes then link to a cost-effectiveness model^12^ evaluating alternative vaccination strategies. Based on the problem space, we configure our model using numerous parameters, including population distribution, vaccine programme and health systems costs for exemplar country contexts.

Our methods extend previous work^5^ with adjusted scenarios to answer policy-relevant questions and include cost-effective analyses. Full model details can be found in the online supplemental material and references.^5 13^ We used the Consolidated Health Economic Evaluation Reporting Standards (CHEERS) checklist when writing this paper.^14^

### Immunological model

Our immunological model is based on the established correlation between neutralising antibodies and protection against COVID-19.^15 16^ We use the model developed by Khoury *et al*^15^ and Cromer *et al*^16^ as implemented by Hao *et al*.^10 17^ This model maps the dynamics of antibodies over the first 250 days since an immune event to protection from disease, including infection acquisition, symptomatic disease, hospitalisation and death. The model uses data on infection acquisition, symptomatic infection, hospitalisation, death and onward transmission from the Delta and Omicron variants to estimate the efficacy of a range of vaccines in the presence and absence of infection.^17^ For this work, we assume a fixed exponential decay rate.^15–17^ See table A1 in the online supplemental material for the immunological parameters.

Evolutionary variants interact with our model through changes in the baseline transmissibility of the virus, and through their ability to ‘escape’ host immunity. While many early variants had transmission advantages over their predecessors, the Omicron variant and its sublineages developed significant ability to escape host immunity compared to other variants. Our models’ parameters are defined to incorporate the properties of the Delta variant. We then incorporate an ‘escape parameter’ to account for the difference between the Omicron BA.1/2-like variant and the Delta variant, and another ‘escape parameter’ for the difference between the Omicron BA.4/5-like variant and Omicron BA.1/2. We estimated escape as a latent parameter in a model based on reinfection and reproduction of the Omicron and Delta variants in South Africa.^17^ Functionally, this escape parameter results in reducing effective individual neutralisation titre against new variants and allows us to combine data on efficacy for multiple variants—in this case Delta and Omicron—in a single model, and thus produce better informed estimates of efficacy at a time when Omicron variants were newly emerging and had scarce data.^17^

For bivalent vaccines, Khoury *et al*^7^ found that variant-adapted vaccines produced, on average, 1.61-fold higher titres than ancestral vaccines. We therefore implement bivalent boosters by using this multiplier on top of the neutralisation titres given by an ancestral mRNA booster vaccine.

### Population transmission model

We model the transmission of SARS-CoV-2 (Omicron BA.1/2-like and BA.4/5-like variants) with an agent-based model adapted from the work of Conway *et al*.^11 13 18^ Each simulated individual has their own neutralising antibodies, age and history of vaccination and infection exposure. Transmission is simulated by directly modelling contact between infectious and susceptible individuals, where the probability of transmission, infection and symptomatic disease is determined by neutralising antibody levels.

We configured two distinct populations, representing typical ‘younger’ and ‘older’ demographics found within the Western Pacific Region.^5^

We implement baseline hybrid immunity over the first 1.5 years by rolling out vaccinations and introducing infection transmission, spending 6-month blocks on distributing the first primary doses, the second primary doses and first booster doses in turn. This 6-month duration is based on the general principle of having 4–6 months between primary doses and booster doses.^19^ Our populations have one of three past vaccination coverage levels, serving as a proxy for both health system capacity and access: low (20% coverage), medium (50% coverage) and high (80% coverage). We introduce Omicron BA.1/2 circulation at around 7 months to develop natural immunity.

Note that populations have either low (15%–45%) or high (80%–100%) past attack rates, corresponding to low or high seroprevalence. We implemented this by varying the transmission potential parameter. This accounts for other factors that can affect transmission potential which we do not explicitly model, such as climate, housing and population density.^20^

After the baseline hybrid immunity is achieved, we test different boosting vaccination strategies between 1.5–3 years after Omicron introduction (around 2023–2024), detailed in Vaccination scenarios. The timing of emergence of the immune escape variant (Omicron BA.4/5-like) is also varied at either 1.5 years, 2 years or 2.5 years. This allows the observation of resurgent epidemic waves driven by waning and/or immune escape. Note that the immune escape variant has a transmission potential multiplier (1.3) on top of the baseline transmission potential and that when the immune escape variant emerges, we also implement a drop in neutralising antibodies (almost threefold) for all individuals, representing the immune escape from Omicron BA.1/2 (see Immunological model and online supplemental appendix A).

### Clinical pathways

The clinical pathways model is based on the framework proposed by Knock *et al*.^21^ We extend this model, building on previous work,^18^ to transform it into an agent-based model. We use the ages and neutralising antibody titres of individuals from the population transmission model, along with information on a variant’s clinical severity, to generate clinical trajectories.^11 13^ The outcomes for individuals include whether they experienced severe disease, required intensive care unit (ICU) admission or died. The duration of hospitalisation in general wards and ICU follows a Gamma distribution, with means and variances sampled from estimates during the Australian Omicron outbreak.^22^ Details of the clinical pathways model are given here.^13^ Note that we assume the clinical severity of the modelled Omicron variants to be the same, and hence they have the same infection fatality rate in a naïve population.

Clinical outcomes are age-dependent. This key assumption, informed by clinical data, suggests that older age groups are at higher risk of severe disease and could have greater benefits from protective vaccination. This premise significantly contributes to our results, particularly regarding cost-effectiveness of vaccination in different population demographics.

### Cost-effectiveness analysis

The cost-effectiveness model^12^ uses outputs from the clinical pathways model. The cost-effectiveness analysis is conducted from a healthcare system perspective, including the following direct medical cost categories:

1. Programmatic costs related to the vaccination intervention, including vaccine dose costs, wastage and delivery costs (logistics, cold chain requirements and transport).
2. Disease management costs at home, in outpatient and inpatient settings for symptomatic COVID-19-related illness.

Costs are estimated for exemplar countries in the Western Pacific Region. We categorise exemplar countries into three distinct groupings with different demographics, health system strength, income levels and vaccine coverage: group A (high income, older population, strong health systems, high vaccination coverage); group B (upper-middle and lower-middle income, younger population, varied health systems, medium or high vaccination coverage); and group C (lower-middle income, younger population, weak health systems, low vaccination coverage).

The sources of unit costs for the cost-effectiveness analysis model are provided in table 1, with full details provided in online supplemental material section D.3. Vaccine dose prices are estimated at $14.20 (range: $3.90–$25.20) for group A, $7.80 (range: $4.00–$12.50) for group B and $7.80 (range: $3.90–$10.00) for group C, based on publicly available estimates from WHO by country income group, reflecting best available estimates as of March 2022. Delivery costs were added to vaccine dose costs to estimate the total cost per vaccine dose administered. The total vaccination programme cost calculation formula is as follows: the number of vaccines delivered × (vaccine dose price+delivery cost per dose) × (1+wastage rate). Take a group A country for example, with an average vaccine cost of $14.20 per dose, a delivery cost of $23.10 per dose and a wastage rate of 10%, the total cost per vaccine dose is $41.44. If 11 000 doses are delivered (see Vaccination scenarios), the total vaccination programme cost is $455 840. We also calculated disease management costs separately for group A (older) and group B/C (younger) countries.

**Table 1:** Source of unit costs for the cost-effectiveness analysis model

Cost	Source	Notes
**Vaccine dose prices**, by country income group (high, upper middle, lower middle)	WHO COVID-19 vaccine price report as of March 2022	Assuming 10% dose wastage rate from UNICEF reports
**Vaccine delivery costs**		
Group A	Governmental reports and peer-reviewed studies	
Group B	Griffiths *et al*^34 38^	
Group C	Same as group B, except doubled at 20% coverage	No country-specific estimates at 20% coverage
**Disease management costs**		
Group A	Publicly available medical fee schedules, published studies, WHO-CHOICE database	
Group B/C	Torres-Rueda *et al*^39^	
**COVID-19-related deaths**	Torres-Rueda *et al*^39^	Only includes cost of body bags

Country groups refer to group A (high income, older population, strong health systems, high vaccination coverage); group B (upper-middle and lower-middle income, younger population, varied health systems, medium or high vaccination coverage); and group C (lower-middle income, younger population, weak health systems, low vaccination coverage).

Health outcomes are presented as disability-adjusted life years (DALYs) using disability weights from the Global Burden of Disease study and Japanese disability weight measurement studies.^23 24^ Duration of illness estimates based on illness severity are from previous studies^25–28^ and estimates of life years lost due to premature mortality are from WHO life tables for each 10-year age band. We use the Japan life table for group A and the global lower-middle-income life table for groups B and C.^29^ Future costs and health outcomes are discounted by 3%. We report costs in 2020 US dollars.

We present cost-effectiveness results of each boosting strategy as incremental cost-effectiveness ratios (ICERs), compared with a ‘no further boosting’ scenario. These ICERs are compared with a range of recently proposed country-specific cost-effectiveness thresholds (CETs) based on health opportunity costs.^30^ We adapted CETs based on 2020 GDP per capita data from the World Bank. Average CETs for group A are $19 000–$30 000, $200–$1600 for group B and $100–$1000 for group C. If a scenario’s ICER falls below the thresholds provided, it is considered likely to be cost-effective.

Deterministic sensitivity analysis and probabilistic sensitivity analysis were conducted to consider the uncertainty in parameters, including reduced care-seeking and/or access to donated vaccines in lower-income settings (see online supplemental material section E).

Our cost-effectiveness analysis has used the CHEERS reporting guidelines.^14^

### Vaccination scenarios

#### High vaccination coverage scenarios

In high-coverage settings (older group A and younger group B demographics), we consider three boosting strategies: paediatric boosting (ages 5–15), high-risk boosting (65+ in the older population and 55+ in the younger population) and random boosting. We fix the number of vaccine doses (11 000) across these scenarios to focus on the impact of vaccine allocation and assume the administration of ancestral monovalent vaccines. This number of doses is sufficient to boost approximately 80% of the 65+ age group in the older population, or approximately 80% of individuals aged 5–15 in the older population. Note that we model all primary doses as monovalent ChAdOx1 nCoV-19 (AstraZeneca) and monovalent booster doses as monovalent BNT162b2 (Pfizer/BioNTech).

We also explore the timing and frequency of high-risk boosting, with boosting occurring at 1.75, 2.0, 2.25 or 2.5 years, or every 6 months starting from 1.75 years.

We also compare additional boosting strategies, where we boost the 65+ age group and expand booster eligibility to younger age groups. This allows us to test the limits of cost effectiveness of extending coverage to lower-risk groups. We do not fix the number of vaccine doses across these scenarios.

#### Low-medium vaccination coverage scenarios

When primary coverage is lower (younger demographic countries in group B with medium coverage and group C with low coverage), we explore three vaccination strategies: new paediatric primary vaccination, high risk boosting (older first) and new random primary vaccinations. We fix the number of vaccine doses (11 000) across these scenarios, administering ancestral monovalent vaccines.

We also consider the impact of switching from monovalent to bivalent vaccines, given that bivalent vaccines are being administered globally.^2 7^ We anticipate that bivalent boosting would have the biggest impact in populations with relatively low vaccine and infection-derived immunity.^7^

### Patient and public involvement

As this is a mathematical modelling study, we did not engage patients or the public.

## Results

### High vaccination coverage scenarios

#### Comparing target use groups

**Figure 2:**
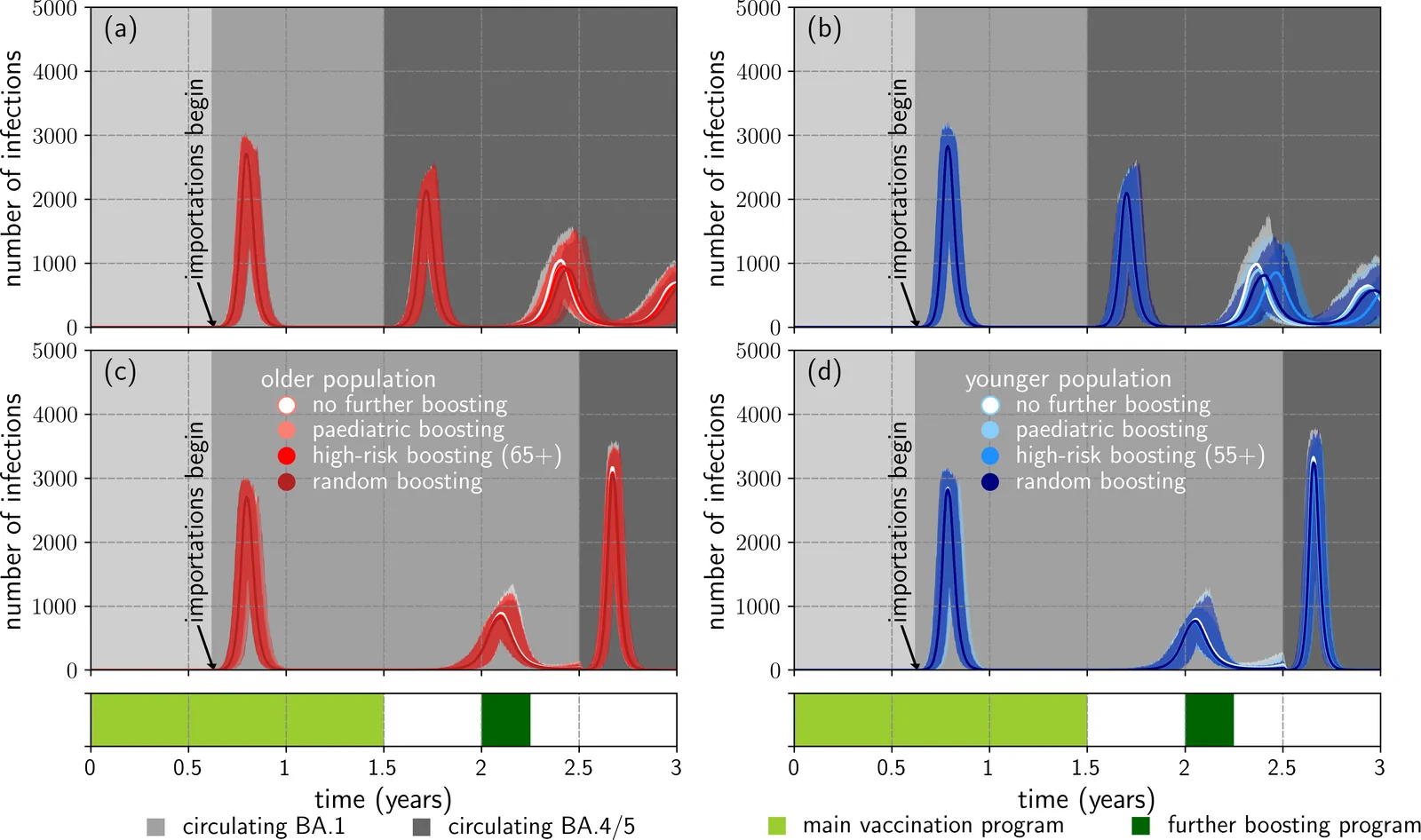
Outbreaks in high transmission settings with high vaccination coverage, for older and younger demographics with early and late seeding of an immune escape variant (dark grey shading). (a) Older population with early immune escape (1.5 years); (b) younger population with early immune escape (1.5 years); (c) older population with late immune escape (2.5 years); (d) younger population with late immune escape (2.5 years). Scenarios (a–d) are run with strategies of no further boosting, paediatric boosting (ages 5–15), high-risk boosting (65+ in the older population, 55+ in the younger population) and random boosting at 2 years. Scenarios are presented with lines representing pointwise medians from 1000 simulations and shaded regions representing the minimum and maximum from the simulations. Results are for a population of 100 000 individuals. The medium grey and dark grey background define the currently circulating variant, Omicron BA.1-like and BA.4/5-like respectively. The impact of boosting on infections is limited.

Figure 2 compares the impacts of alternative vaccine allocations in older and younger populations depending on the time of immune escape emergence (1.5 vs 2.5 years), given past (1.5 years) high seroprevalence. We find limited impact of different strategies on infection dynamics. In the late (2.5 years) immune escape scenarios (figure 2c,d), the epidemic peaks are shifted to the right, but overall the infection curves maintain the same qualitative shape.

We find that vaccination has greater impact on severe outcomes. Across the scenarios, high-risk boosting averts the most severe disease (table 2).

**Table 2:** Median number of deaths and cost-effectiveness results for boosting strategies compared with no further boosting in a population of 100 000 individuals over 1.5–3 years, in high transmission settings, high vaccination coverage and the immune escape variant emerging at either 1.5 years (early) or 2.5 years (late)

Boosting strategy	Median deaths*	Total costs ($)	Total DALYs	Incremental costs ($)	DALYs averted	ICER ($ per DALY averted)†
Older population, early immune escape						
No further boosting	40 (28–53)	5 775 662	607.7	–	–	–
Paediatric (age 5–15)	40 (29–53)	6 160 852	616.5	385 190	−8.8	Dominated
High-risk (age 65+)	34 (23–46)	5 722 929	536.9	−52,733	70.8	Dominant
Random	39 (27–52)	5 901 691	594.1	126 029	13.6	9283
Younger population, early immune escape						
No further boosting	11 (5–18)	2 419 793	187.1	–	–	–
Paediatric (age 5–15)	12 (6–18)	2 567 955	191.4	148 162	−4.3	Dominated
High-risk (age 55+)	9 (4–15)	2 274 229	150.4	−145 564	36.7	Dominant
Random	11 (5–18)	2 484 042	186.4	64 249	0.7	96 182
Older population, late immune escape						
No further boosting	49 (36–62)	6 099 953	746.8	–	–	–
Paediatric (age 5–15)	49 (36–62)	6 386 931	739.2	286 978	7.6	37 685
High-risk (age 65+)	34 (23–47)	5 700 515	558.0	−399 438	188.8	Dominant
Random	44 (32–59)	6 171 180	674.7	71 227	72.1	988
Younger population, late immune escape						
No further boosting	15 (8–24)	2 239 512	237.4	–	–	–
Paediatric (age 5–15)	17 (9–25)	2 393 653	250.0	154 141	−12.6	Dominated
High-risk (age 55+)	9 (4–16)	2 216 146	157.6	−23 367	79.8	Dominant
Random	15 (8–23)	2 342 591	230.2	103 079	7.2	14 360

* Values in parentheses represent 0.025 and 0.975 quantiles from 5000 simulations (five clinical pathway simulations are produced for each of the 1000 infection transmission simulations). Each scenario is run with four different boosting strategies, with boosting occurring at 2 years.

† The ICER is calculated as incremental costs divided by the DALYs averted. A strategy *dominates* no further boosting when it is less costly and averts DALYs. Likewise, a strategy is *dominated* by no further boosting when it is more costly and incurs DALYs.

DALYs, disability-adjusted life years; ICER, incremental cost-effectiveness ratio.

**Figure 3:**
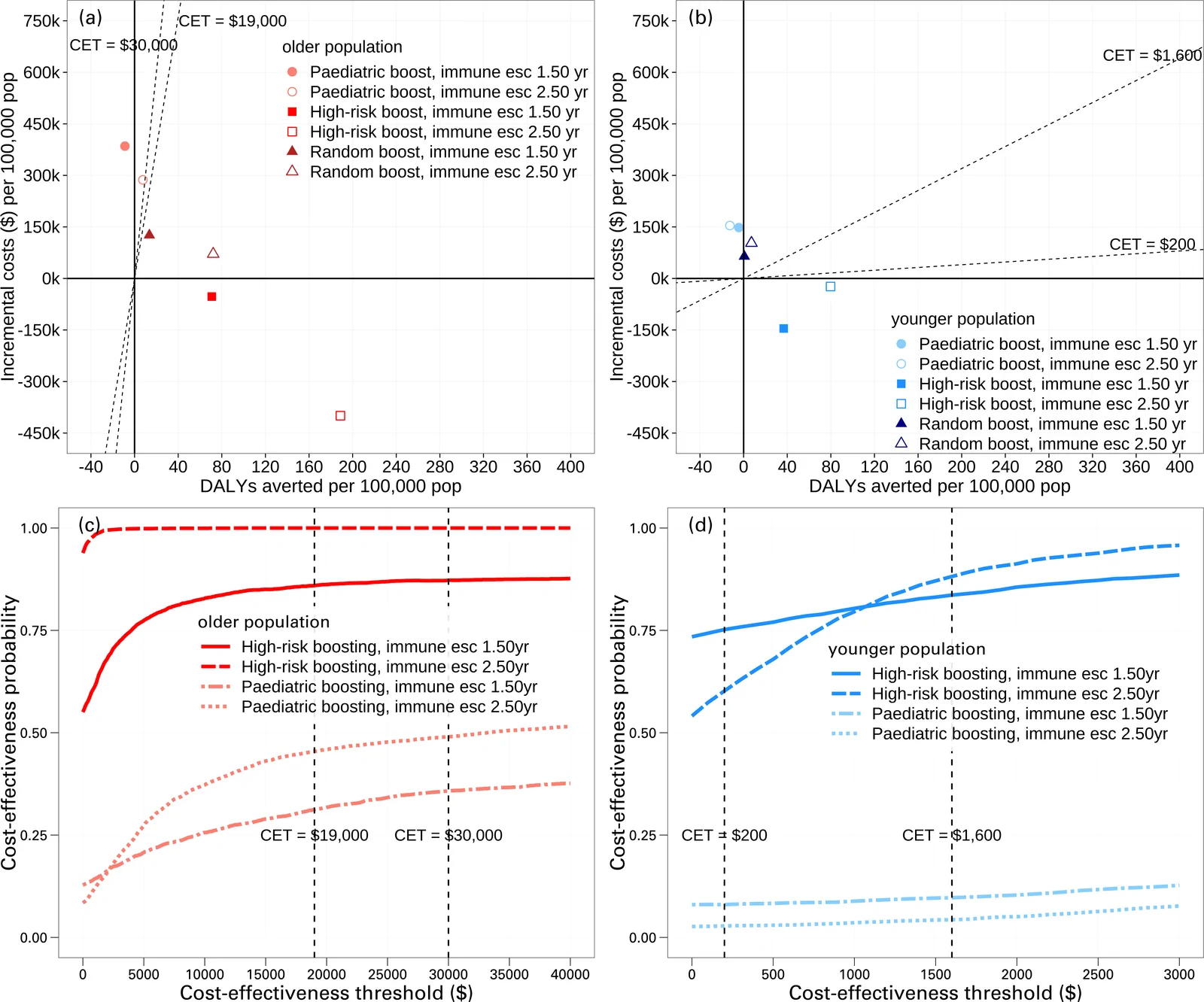
Cost-effectiveness results for different vaccination strategies in high transmission high vaccination coverage settings, for older (group A) and younger (group B) demographics with early (1.5 year) and late (2.5 year) seeding of an immune escape variant. Top figures represent cost-effectiveness planes for (a) older population and (b) younger population. Bottom figures show cost-effectiveness acceptability curves considering stochastic uncertainty and economic parameter uncertainty for the most cost-effective high-risk strategies for (c) older population and (d) younger population. The boosting strategies considered here are: paediatric boosting (ages 5–15), high-risk boosting (65+ in the older population, 55+ in the younger population) and random boosting at 2 years. Results are for a population of 100 000 individuals. High-risk boosting is the most cost-effective strategy and dominates paediatric and random boosting strategies. CET, cost-effectiveness threshold; DALYs, disability-adjusted life years.

In figure 3a,c, we find that in older populations, boosting is more cost-effective when it occurs prior to immune escape. High-risk boosting is likely to be highly cost-effective or cost-saving, while paediatric boosting does not appear to be cost-effective. Random boosting does worse than high-risk boosting. Cost-effectiveness of high-risk boosting is driven primarily by vaccine programme (delivery and dose) costs, followed by disease management costs in general ward (online supplemental material figure E5).

High-risk boosting *may* be cost-effective or even cost-saving in younger middle-income country (MIC) populations, depending on country level willingness to pay (WTP) per DALY averted threshold, or CET, and other key model inputs, which we see in figure 3b,d. These results are driven primarily by home-based care cost inputs and vaccine delivery costs, which remain highly uncertain in these settings (online supplemental material figure E6). In sensitivity analyses investigating no home-based care costs in these settings, we find elder boosting strategies may remain cost-effective when the vaccine is donated and for countries with higher CETs (online supplemental material figure E7).

The relative benefits of boosting, especially high-risk boosting, hold across all the scenarios considered in figures 2 and 3 and table 2. However, the absolute benefits are greater in the older population.

Note that we assume high transmission potential, which leads to high seroprevalence. The relative impact of vaccination in the low seroprevalence scenario is similar (online supplemental material section E1).

#### Boosting frequency

**Figure 4:**
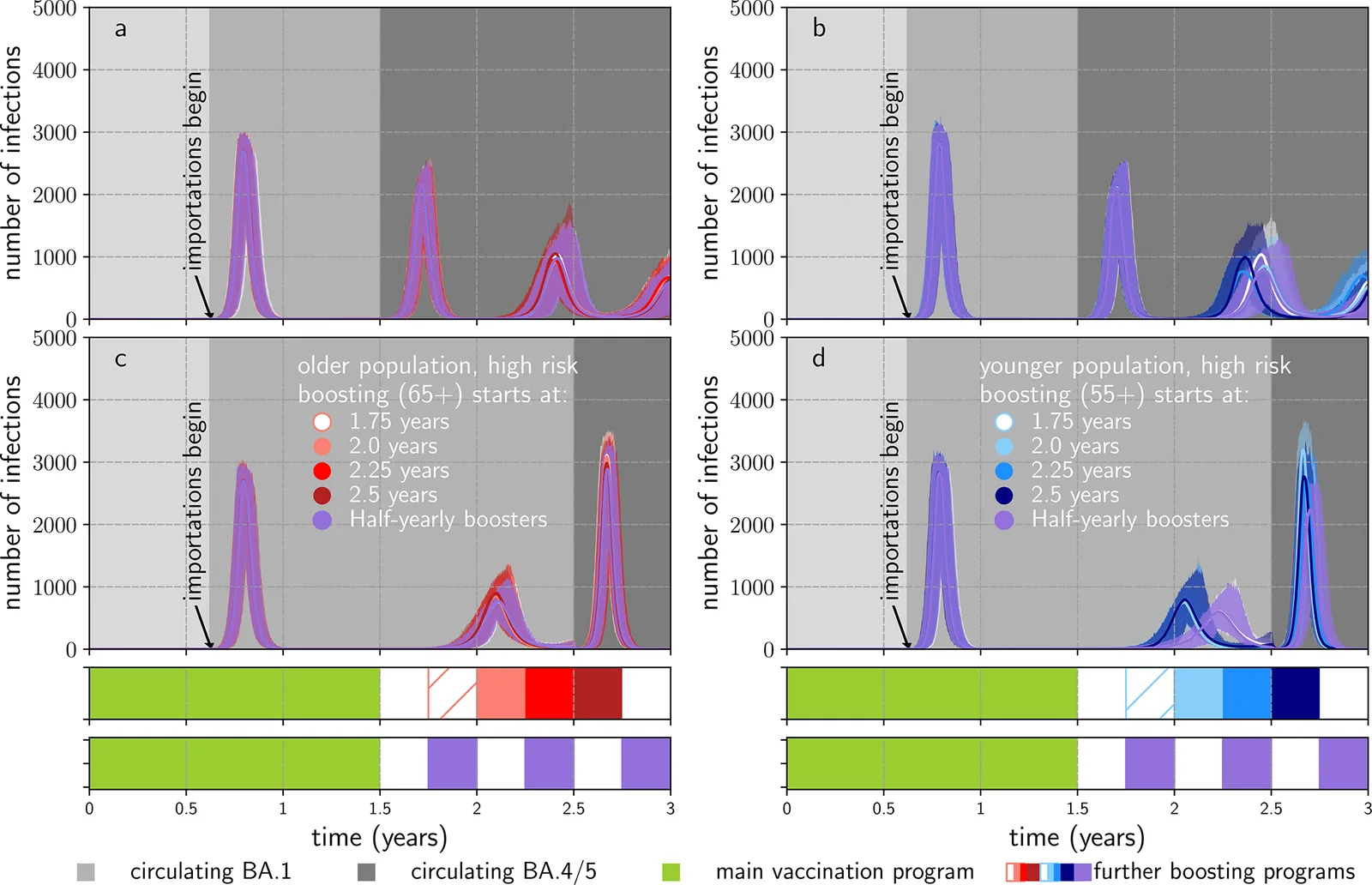
Outbreaks in high transmission settings with high vaccination coverage, for older and younger demographics, comparing boosting once at a range of times and frequent boosting. (a) Older population with early immune escape (1.5 years); (b) younger population with early immune escape (1.5 years). (c) older population with late immune escape (2.5 years). (d) younger population with late immune escape (2.5 years). Scenarios (a–d) are run with high-risk boosting (65+ in the older population, 55+ in the younger population) rolled out at either 1.75 years, 2.0 years, 2.25 years, 2.5 years or half-yearly starting from 1.75 years. Scenarios are presented with lines representing pointwise medians from 1000 simulations and shaded regions representing the minimum and maximum from the simulations. Results are for a population of 100 000 individuals. The timing and frequency of boosting, relative to emergence of the immune escape variant, influences the impact of vaccination on the subsequent outbreaks.

The timing and frequency of boosting, relative to emergence of the immune escape variant, influenced the impact of vaccination on the number of infections (figure 4). Half-yearly boosting consistently achieves fewer severe outcomes when compared with boosting once (see online supplemental material table E4).

The cost-effectiveness of high-risk boosting varies according to immune escape timing, but generally is very cost effective or cost-saving in the older (HIC) and younger (MIC) populations. Half-yearly boosting remains highly cost-effective in older populations, but more expensive than boosting only once (online supplemental material figure E8).

However, half-yearly boosting is unlikely to be cost-effective for younger (MIC) countries with high vaccine coverage (∼80%) unless vaccines are donated (online supplemental material figure E8). These results were driven by home-based care costs (lower costs indicate half-yearly boosting is unlikely to be cost-effective) and vaccine programme costs (lower costs, e.g., donated vaccines, would mean half-yearly boosting may be very cost-effective or cost-saving) (online supplemental material figure E9).

#### Age cut-off for cost-effective boosting

**Figure 5:**
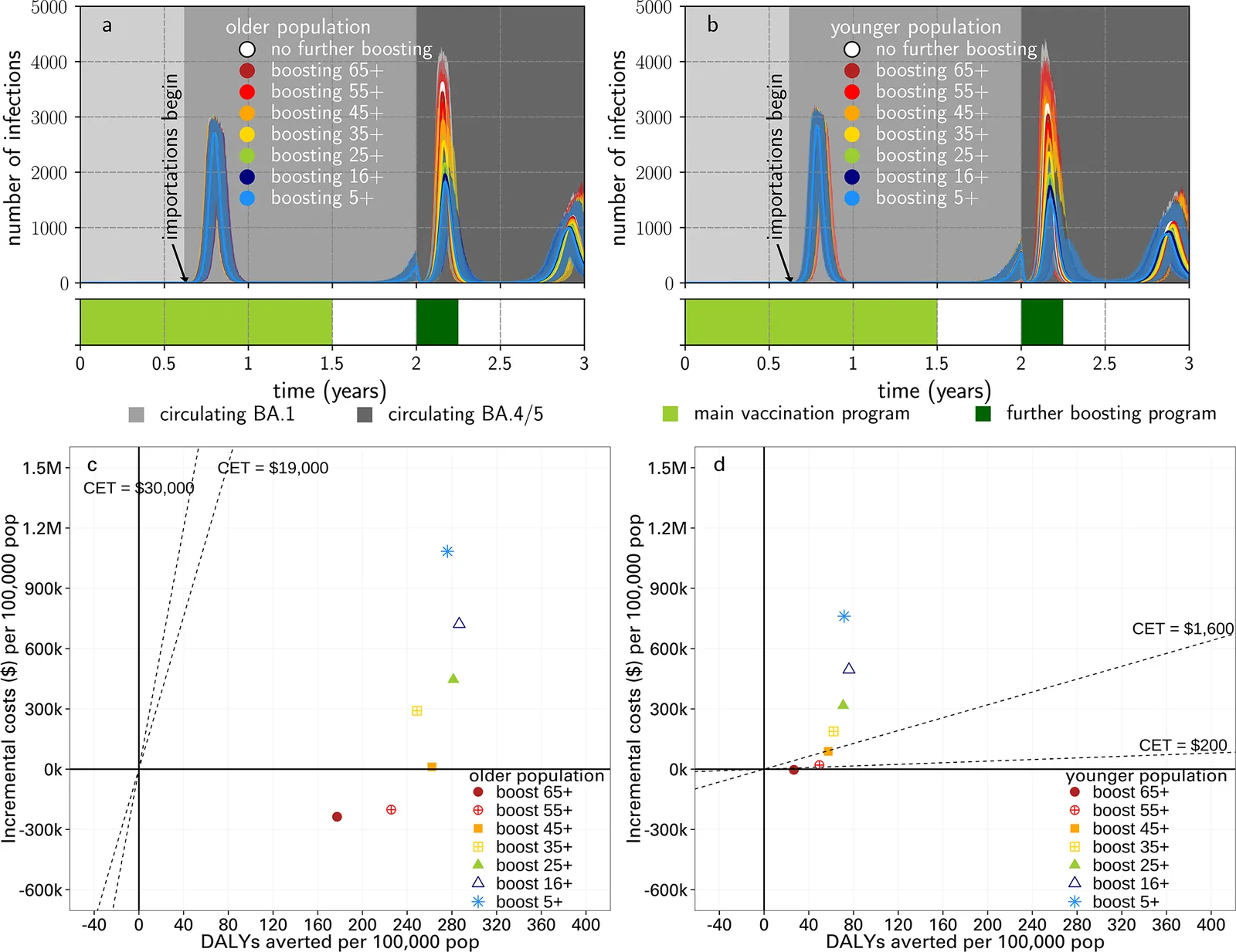
Outbreaks and cost-effectiveness analyses in the high transmission high vaccination coverage setting, for older and younger demographics, comparing the impact of lowering the age cut-off for high-risk boosting. (a) Epidemic waves in the older population; (b) epidemic waves in the younger population; (c) cost-effectiveness analysis in the older population; (d) cost-effectiveness analysis in the younger population. All scenarios here had an immune escape variant seeded at 2 years, with boosting at 2 years. The solid lines in (a) and (b) represent the pointwise median infections from 1000 simulations and the shaded regions represent the pointwise maximum and minimum infections. Results are for a population of 100 000 individuals. CET, cost-effectiveness threshold; DALYs, disability-adjusted life years.

We systematically expanded booster eligibility to younger age groups (figure 5). The difference in health outcomes between boosting 45+ and younger age groups is minimal (see online supplemental material table E5), but increasing the age cohorts in the programme leads to higher costs and thus lower cost effectiveness. Boosting 65+ and 55+ is likely highly cost-effective or cost-saving (figure 5). Boosting 45+ is also likely highly cost-effective (online supplemental material figure E11).

### Low-medium vaccination coverage scenarios

#### Comparing primary and booster strategies

**Figure 6:**
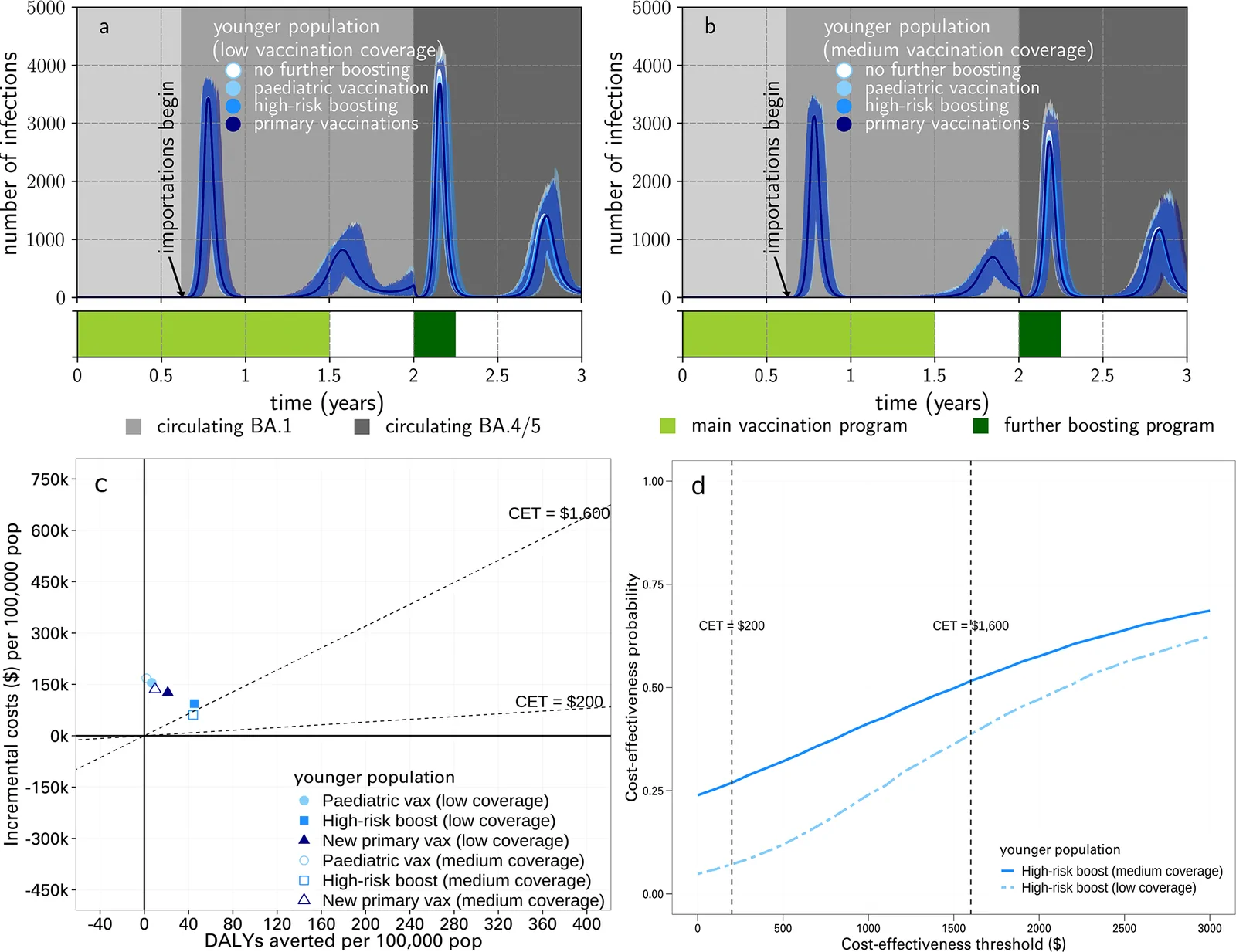
Outbreaks and cost-effectiveness analyses in the high transmission setting, with low and medium vaccination coverage for younger demographics. (a) Epidemic curves for younger population with low vaccination coverage (initial coverage 20%). (b) for medium coverage (initial coverage 50%); (c) cost-effective analyses for low and medium vaccination coverage scenarios; (d) cost-effectiveness acceptability curves considering stochastic uncertainty and economic parameter uncertainty. All scenarios had an immune escape variant seeded at 2 years. These scenarios are run with vaccination strategies of paediatric vaccination (ages 5–15), high-risk boosting (65+ first) and new primary vaccinations (allocated randomly among ages 5+) at 2 years. The solid lines in (a) and (b) represent the pointwise median infections from 1000 simulations and the shaded regions represent the pointwise maximum and minimum infections. Results are for a population of 100 000 individuals. Paediatric and new primary vaccinations are unlikely to be cost-effective. CET, cost-effectiveness threshold; DALYs, disability-adjusted life years.

We consider the trade-off between new primary vaccination and high-risk boosting strategies. We find that high-risk boosting strategies perform slightly better, reducing the height of epidemic peak after the 2-year mark (figure 6a,b). Online supplemental material table E6a,b shows a small reduction in deaths under the high-risk boosting strategy compared with other strategies.

New primary paediatric vaccination and new primary vaccination strategies are unlikely to be cost-effective (figure 6c). High-risk boosting strategies may be cost-effective for younger (MIC) countries with high WTP thresholds or CETs (figure 6d), but this depends on unit cost inputs, which are driven primarily by home-based care and vaccine programme costs (online supplemental material figure E14).

#### Impact of bivalent boosting

We found a modest benefit in bivalent boosters over monovalent boosters on the dynamics of infections, with very minor differences in infection peak height (see online supplemental material figure E15) and also minor differences in the number of deaths between vaccine types (online supplemental material table E7).

Given that the modelled benefits of bivalent boosters are minor compared with monovalent formulations, we anticipate that bivalent vaccines in this context will not be cost-effective unless WTP is high. As such, we have not subjected these scenarios to formal cost-effectiveness analyses.

## Discussion

COVID-19 disease is globally endemic, requiring ongoing management. However, governments and policymakers face competing health priorities that need to be addressed in the context of resource constraints. With populations now having high infection-acquired immunity due to the spread of Omicron, guidance on whether COVID-19 vaccination programmes should continue, who should be prioritised and how frequently vaccination should occur, is needed.

We used modelling to explore the epidemiological and cost impacts of COVID-19 booster dose administration in diverse population settings, based on the demographics and experiences of the Western Pacific Region. We assumed differing levels of prior vaccination delivery and infection experience, linked to income group level characteristics and vaccine programme and healthcare costs. Given the age dependency of severe disease risk, we concluded that elder-targeted strategies are most likely to be cost-effective (or even cost-saving) across a broad range of uncertainties. Notably, we consistently found paediatric programmes (primary series or boosting) are not cost-effective. Absolute harms averted by vaccination are influenced by age and risk profile of the population, prior immune landscape (infection exposure history, vaccination rollout) and timing of emergence of an immune escape variant in relation to booster delivery. Half-yearly ‘high risk’ booster programmes are more expensive but may be cost effective in older, high-income populations. However, this finding is much more uncertain in populations with younger demographics (representing upper- and lower-middle income countries), depending on the costs of home-based care and vaccine implementation.

Our comprehensive analysis was possible due to the flexibility of our modelling approach, which enabled distinct configuration of the various interweaving elements. We used the immunity model to implement assumptions relevant to two exemplar Omicron variants, against which the effectiveness of ancestral and bivalent vaccines was explored. By introducing variants at different time points, we systematically evaluated the importance of epidemic timing in relation to plausible immune escape scenarios. Separate representation of clinical pathways and cost-effectiveness analysis further enabled adaptation to different settings. Health sector costs were exemplified by ‘averages’ based on regional data, with sensitivity analyses highlighting local drivers that will be influential for decision making depending on context.

As with all models, multiple simplifying assumptions were made for the purpose of tractability that do not necessarily reflect reality. We assumed circulation of a single dominant SARS-CoV-2 variant, to interrogate the ‘worst case’ scenario of a step change in vaccine effectiveness at a single point in time, resulting in high numbers of infection and impact. The present state of multiple lineage cocirculation with variable immunological cross-reactivity and breadth likely lessens such impacts.^31^ We could have explored more optimistic values for bivalent vaccine effectiveness, which may lead to greater estimated benefits.^32^ We could have also explored more seroprevalence scenarios by controlling the transmission levels.^5^ Neither social restrictions nor antiviral agents were included, given that neither are presently being widely applied at the population level globally. Long COVID was not included as a potential outcome of infection, as there remains limited quantitative data of this clinical burden. The anticipated costs of therapeutic pathways are yet to be determined pending identification of those which are most likely to be effective for various syndromic presentations.

A more significant limitation of our work in relation to health system costs is the assumption that the modelled adult targeted vaccination coverage is achievable and can be costed across all country settings considered. In reality, most lower-middle-income countries do not have adult immunisation programmes in place, as highlighted by the COVID-19 pandemic. This deficiency is a global public health priority. We also do not consider indirect costs to society, such as productivity losses, associated with illness or death. A societal perspective would increase the cost-effectiveness of elder-targeted vaccination, but the development of adult immunisation programmes would decrease the cost-effectiveness. Our estimated price of COVID-19 vaccines was based on publicly available estimates from WHO from 2022 by income group; however, these estimates may reflect lower pricing available during the pandemic period. Recent (2024) COVID-19 vaccine dose prices available to low-income and middle-income countries through Pan American Health Organization Revolving Fund remain consistent with the maximum vaccine prices we explored in one-way sensitivity analyses.^33^ However, these prices may underestimate current vaccine prices in some middle-income and high-income settings, and thus our modelling may have underestimated the costs associated with ongoing COVID-19 vaccination programmes and overestimated the cost-effectiveness of vaccination in these settings. Furthermore, we assumed a 10% vaccine wastage rate. However, as witnessed during the pandemic, large quantities of vaccine procurement in high-income countries may lead to potential excess and expiration and may reduce the cost-effectiveness of boosting. Our choice was based on COVID-19 Vaccines Global Access, who had considered a vaccine wastage rate of 10%^34^; however, a report by the Australian Department of Health and Aged Care had noted a wastage rate of 18.2% in Australia.^35^

Our study considers a diversity of demographics and hybrid immunity histories, with lessons offered for countries that may fall between the ‘older’ and ‘younger’ exemplar groupings. We note that all our considered populations had some level of background vaccination (22%) by the end of the first Omicron wave; our work complements other studies^36 37^ that have considered the zero past vaccination setting with prior natural immunity in low-income and middle-income countries.

As the world transitions towards COVID-19 endemicity, our study demonstrates the ongoing value of COVID-19 booster doses targeted towards older age groups at risk of severe outcomes in a range of demographics with different hybrid immunity histories. Vaccinating those most at risk is cost-effective or even cost-saving across multiple country settings. However, this assumes that adult immunisation programmes are in place and do not impact on delivery of other services. Paediatric vaccination may be more readily implementable within existing health systems but was not cost-effective in any of the scenarios explored. These results were presented to the Advisory Committee on Immunization and Vaccines-related Implementation Research,^8^ with our work being subsequently cited as part of the WHO updated COVID-19 vaccination guidance for March 2023.^9^

SARS-CoV-2 reporting is being subsumed into broader respiratory pathogen surveillance systems. The WHO continues to encourage vigilance for identification of new variants with heightened transmissibility or pathogenic potential. A downward age shift in disease severity would require revision of current strategies. Modelling can be used to analyse the impact of potential future strategies. Our framework is sufficiently flexible to incorporate emerging evidence of virus and vaccine characteristics and can be configured to specific country settings. More broadly, it provides a template for the use of modelling to evaluate strategies for the control of any emerging or epidemic infectious disease and support decision-making.

## Supplementary material

10.1136/bmjgh-2024-016096 online supplemental file 1

## Data Availability

Data are available in public, open access repositories.
