# Supplementary material for: Health impact and cost-effectiveness of COVID-19 booster vaccination strategies in the early post-Omicron era: a dynamic modelling study
Source: BMJ Glob Health. 2025 Sep 29;10(9):e016096. doi: 10.1136/bmjgh-2024-016096 (PMC12481342; doi:10.1136/bmjgh-2024-016096)
Supplement: online supplemental file 1 [file bmjgh-10-9-s001.pdf]

Health impact and cost-effectiveness of COVID-19 booster vaccination strategies in the early post-Omicron era: a dynamic modelling study
Supplementary Material

Thao P. Le[1,2,3], Eamon Conway[4], Edifofon Akpan[5], Isobel Abell[1,2], Patrick Abraham[5], Christopher M. Baker[1,2,3], Patricia T. Campbell[5,6], Deborah Cromer[7], Michael J. Lydeamore[8], Yasmine McDonough[4], Ivo Mueller[4,9], Gerard Ryan[5,10], Camelia Walker[1,5], Yingying Wang[5], Natalie Carvalho[5,‡], and Jodie McVernon[6,11,‡]

[1]School of Mathematics and Statistics, The University of Melbourne, Melbourne, Victoria, Australia
[2]Melbourne Centre for Data Science, The University of Melbourne, Melbourne, Victoria, Australia
[3]Centre of Excellence for Biosecurity Risk Analysis, The University of Melbourne, Melbourne, Victoria, Australia
[4]Population Health & Immunity Division, Walter and Eliza Hall Institute of Medical Research, Melbourne, Victoria, Australia
[5]Melbourne School of Population and Global Health, The University of Melbourne, Melbourne, Victoria, Australia
[6]Department of Infectious Diseases at the Peter Doherty Institute for Infection and Immunity, The University of Melbourne, Melbourne, Victoria, Australia
[7]Kirby Institute, University of New South Wales, Sydney, New South Wales, Australia
[8]Department of Econometrics and Business Statistics, Monash University, Melbourne, Victoria, Australia
[9]Department of Medical Biology, The University of Melbourne, Melbourne, Victoria, Australia
[10]Telethon Kids Institute, Nedlands, Western Australia, Australia
[11]Victorian Infectious Diseases Reference Laboratory Epidemiology Unit, The Royal Melbourne Hospital at the Peter Doherty Institute for Infection and Immunity, Melbourne, Victoria, Australia
[‡]These authors should be considered as joint senior author.

CONTENTS

A Immunological model **3**
A.1 Neutralising antibody titres . . . 3
A.2 Protection against disease outcomes . . . 3
A.3 Probability of disease outcomes . . . 6

B Population transmission model **8**
B.1 Scenario and input parameters . . . 8
B.1.1 Population types . . . 8
B.1.2 Transmission potential . . . 9
B.1.3 Variants and immune escape . . . 9
B.1.4 Vaccination . . . 10
B.1.5 High coverage boosting strategies . . . 10
B.1.6 Low coverage vaccination and boosting strategies . . . 11
B.1.7 Boosting strategies for the age-cutoff-investigation . . . 11
B.1.8 Bivalent boosting . . . 11

C Clinical pathways **15**

D Cost-effectiveness analysis **16**
D.1 CEA model overview . . . 16
D.2 Defining exemplar country contexts for cost-effectiveness analysis . . . 16
D.3 Resource use and costs . . . 18
D.3.1 COVID-19 vaccine dose cost . . . 18
D.3.2 COVID-19 vaccine delivery cost . . . 19
D.3.3 COVID-19 treatment cost . . . 21
D.4 Health Outcomes . . . 23
D.5 Cost-effectiveness analysis . . . 24
D.5.1 Cost-effectiveness thresholds . . . 24
D.5.2 Cost-effectiveness results and interpretation . . . 25
D.5.3 Limitations . . . 26

E Supplementary results **27**
E.1 High vaccination coverage scenarios: comparing target use groups - extended results . . . 28
E.2 High vaccination coverage scenarios: Boosting frequency - extended results . . . 36
E.3 High vaccination coverage scenarios: age-cutoff for cost-effective boosting - extended results . . . 39
E.4 Low-medium vaccination coverage: comparing primary and booster strategies - extended results . . . 42
E.5 Low-medium vaccination coverage: impact of bivalent boosting - extended results . . . 46

References **48**

A IMMUNOLOGICAL MODEL

Within the model, each individual has their own neutralising antibody titre value. This allows for a distribution of protection in the population, which in turn impacts the epidemic dynamics [1]. Khoury and colleagues [2, 3] developed a model of correlates of protection relating an individual's neutralising antibody titre to their protection against symptomatic and severe disease outcomes. Here we use Golding and colleagues' [4] implementation of that model which expands the model to other outcomes in a Bayesian framework. Using data on efficacy and time since vaccination, we can estimate the relationship between neutralising antibody titres and protection against clinical outcomes: infection, symptomatic disease, onward transmission given breakthrough infection, hospitalisation and death.

In particular, Golding and colleagues [4] used neutralising antibody level data and vaccine efficacy estimates to estimate neutralising antibody levels and efficacy against the *Delta* variant for various exposure scenarios through time. An additional 'escape' parameter was estimated to determine efficiacies for the Omicron variant relative to the Delta variant. Golding and colleagues also modelled waning of neutralising antibodies and efficacy for the Delta and Omicron variants [4].

For more details about the immunological model, see [4] and [5]. In the following, we briefly describe the neutralising antibody titres, the protection again disease outcome calculations, and the subsequent probabilities of disease. Table A1 lists the parameter values used.

A.1 Neutralising antibody titres

An individual's neutralising antibody titre value can be increased by different 'exposures'. In the model, this can occur due to: (i) a first, second or booster dose of a vaccine or (ii) an infection. We assume that the neutralising antibody titre is the same for a given combination vaccination doses and infection, regardless of the order of infection and vaccination.

Let μ_j^x describe the log of the mean neutralising antibody titre against strain x (Delta, Omicron BA1-like, or Omicron BA4/BA5-like) after exposure process j (vaccinations, boosting and infections). All mean neutralising antibody titres (in $\log_{10}$) used can be found in Table A1. Note our values are relative to the Delta strain ($x = 0$). To convert to antibody titres against the Omicron strains, we use:

$$\mu_j^{\text{Omicron}} = \mu_j^0 + \log_{10}(f_{\text{Omicron}}), \quad (1)$$

$$\mu_j^{\text{Omicron-escape}} = \mu_j^0 + \log_{10}(f_{\text{Omicron-escape}}), \quad (2)$$

where f_{Omicron} and $f_{\text{Omicron-escape}}$ describe the relative change in antibody titre to the Delta strain.

Using the values of μ_j^x (Table A1), we sample the neutralising antibody titre for individual i, a_i^0, after some exposure j with:

$$\log_{10}(a_i^0) \sim \mathcal{N}(\mu_j^x, \sigma^2), \quad (3)$$

where $\mathcal{N}$ denotes the normal distribution, μ_j^x is $\log_{10}$ of the mean neutralising antibody titre after exposure j to strain x, and σ^2 is the variance of neutralising antibodies. We assume that the antibody titre $a_i(t)$ will exponentially decay through time from the titre after exposure a_i^0 as:

$$\log_{10}(a_i(t)) = \log_{10}(a_i^0) - \frac{k_a}{\log(10{\cdot}0)} t, \quad (4)$$

where k_a is the decay rate and t is the time since the most recent exposure (vaccination or infection).

A.2 Protection against disease outcomes

Using the neutralising antibody titres, we calculate protection ρ_α against disease outcome α:

$$\rho_\alpha = \frac{1}{1 + \exp(-k(\log_{10}(a_i) - c_\alpha))}, \quad (5)$$

where k relates to the steepness of the logistic curve, c_α is the midpoint of the logistic (Table A1), and a_i is the considered individual i's current antibody titre.

The realised conversions of neutralising antibody titre to vaccine protection against disease and severe outcomes are shown in Figure 4 of Hao et al. [6] (which we have reproduced here in Figure A1), which presents the estimated immune protection against Omicron over time after peak immunity for different vaccines and doses.

Table A1: Estimated and derived parameter values from the immunological model used in the simulations. Note that the age brackets for baseline probabilities (symptomatic infection, relative infectiousness, susceptibility, hospitalisation, ICU admission, and death) correspond to [0, 5, 10, 15, 20, 25, 30, 35,40, 45, 50, 55, 60, 65, 70, 75, 80]. Parameter value sources: [4, 5, 7]. Note that the case-fatality ratio for Omicron is known to be much lower compared to pre-Omicron case-fatality ratios [8]. See Figure 4 by Hao et al. [6] (Figure A1) for the estimated immune protection against Omicron over time after peak vaccine immunity.

Parameter: description	Value(s)
μ_U^0: $\log_{10}$ of the mean neutralising antibody titre after infection whilst unvaccinated	$0{\cdot}0$
μ_{AZ1}^0: $\log_{10}$ of the mean neutralising antibody titre after the first dose of AstraZeneca (no infection)	$-0{\cdot}529\,952\,209\,575\,501\,3$
μ_{AZ2}^0: $\log_{10}$ of the mean neutralising antibody titre after the second dose of AstraZeneca (no infection)	$-0{\cdot}120\,317\,133\,121\,800\,76$
μ_{P1}^0: $\log_{10}$ of the mean neutralising antibody titre after the first dose of Pfizer (no infection)	$-0{\cdot}231\,541\,325\,455\,433\,96$
μ_{P2}^0: $\log_{10}$ of the mean neutralising antibody titre after the second dose of Pfizer (no infection); also the mean titre after one AZ dose and one infection	$0{\cdot}154\,016\,690\,200\,059\,7$
μ_{B}^0: $\log_{10}$ of the mean neutralising antibody titre after the first mRNA booster dose (without infection); also the mean titre after two AZ doses and one infection	$0{\cdot}322\,553\,889\,906\,838\,3$
μ_{BB}^0: $\log_{10}$ of the mean neutralising antibody titre after the first mRNA bivalent booster dose (without infection)	$0{\cdot}529\,379\,765\,93$
$\log_{10}(f_{\text{Delta}})$: $\log_{10}$ of the fold change in neutralising antibody titre between Delta and the baseline, Delta	0.0
$\log_{10}(f_{\text{Omicron}})$: $\log_{10}$ of the fold change in neutralising antibody titre between Delta and Omicron (BA1-like)	$-0{\cdot}692\,380\,817\,438\,403\,1$
$\log_{10}(f_{\text{Omicron-escape}})$: $\log_{10}$ of the fold change in neutralising antibody titre between the Delta and the BA4/BA5-like immune escape variant	$-1{\cdot}180\,497\,456\,46$
σ: standard deviation of the $\log_{10}$ of neutralising antibodies across the population	$0{\cdot}464\,709\,2$
$\log(k)$: governs the logistic curve steepness relating antibodies to protection against disease outcome	$1{\cdot}686\,059\,432\,639\,791$
k_a: decay rate of neutralising antibodies	$0{\cdot}008\,235\,096\,361\,537\,353$
c_h: midpoint of the logistic function for protection against hospitalisation	$-1{\cdot}216\,178\,672\,514\,781\,4$
c_d: midpoint of the logistic function for protection against death	$-1{\cdot}175\,315\,140\,567\,729\,3$
c_ξ: midpoint of the logistic function for protection against acquisition	$-0{\cdot}471\,959\,626\,519\,071\,75$
c_τ: midpoint of the logistic function for protection against transmission	$0{\cdot}029\,536\,834\,493\,436\,93$
c_q: midpoint of the logistic function for protection against symptoms	$-0{\cdot}644\,202\,077\,390\,790\,3$
q_i^0: baseline probability of symptomatic infection across age groups	[0·29, 0·29, 0·21, 0·21, 0·27, 0·27, 0·33, 0·33, 0·4, 0·4, 0·49, 0·49, 0·63, 0·63, 0·69, 0·69, 0·69]
β_i: baseline relative infectiousness across age groups	[0·799 15, 0·687 631, 0·675 465, 0·756 167, 0·918 134, 0·965 157, 0·947 093,0·932 175, 0·933 528, 0·939 668, 0·953 521, 0·981 757, 1·0, 0·998 492, 0·989 752, 0·973 762, 0·943 804]
ξ_i^0: baseline susceptibility across age groups	[0·301 016, 0·367 215, 0·432 601, 0·527 461, 0·764 291, 0·923 87, 0·982 666, 0·974 271, 0·931 88, 0·914 635, 0·928 871, 0·962 179, 1·0, 0·972 011, 0·882 005, 0·823 837, 0·802 185]
$p_{H\|E}^0$: baseline probability of hospitalisation given infection across age groups, for Omicron	[0·0011, 0·000 03, 0·0001, 0·0002, 0·0007, 0·0011, 0·0014, 0·0015, 0·0020, 0·0030, 0·0067, 0·0094, 0·0144, 0·0224, 0·0337, 0·0460, 0·0421]
$p_{ICU\|E}^0$: baseline probability of requiring the ICU given infection, for Omicron	[0·000 036, 0·000 001, 0·000 005, 0·000 009, 0·000 040, 0·000 070, 0·000 106, 0·000 129, 0·000 201, 0·000 340, 0·000 841, 0·001 249, 0·001 905, 0·002 728, 0·003 397, 0·003 327, 0·000 841]

Parameter: description	Value(s)
$p^0_{H_D\|E}$: baseline probability of death on ward (without visiting ICU) given infection, for Omicron	[0·000 005 4, 0·000 000 1, 0·000 000 5, 0·000 000 8, 0·000 002 9, 0·000 004 8, 0·000 007 2, 0·000 009 2, 0·000 016 2, 0·000 033 6, 0·000 109 3, 0·000 233 2, 0·000 553 6, 0·001 331 0, 0·003 017 1, 0·005 613 2, 0·006 194 8]
$p^0_{ICU_D\|E}$: baseline probability of dying in the ICU given infection, for Omicron	[0·000 003 5, 0·000 000 1, 0·000 000 6, 0·000 001 0, 0·000 004 3, 0·000 008 0, 0·000 013 1, 0·000 017 7, 0·000 031 5, 0·000 062 3, 0·000 181 3, 0·000 316 9, 0·000 560 0, 0·000 908 6, 0·001 234 1, 0·001 243 7, 0·000 274 5]
$p^0_{W_D\|E}$: baseline probability of dying in the ward after returning from the ICU given infection, for Omicron	[0·000 000 48, 0·000 000 01, 0·000 000 06, 0·000 000 10, 0·000 000 43, 0·000 000 76, 0·000 001 18, 0·000 001 51, 0·000 002 41, 0·000 004 17, 0·000 010 99, 0·000 018 52, 0·000 035 87, 0·000 071 77, 0·000 127 61, 0·000 167 66, 0·000 060 57]
R_0 ratio between the original Omicron variant (BA1-like) and the BA4/BA5-like immune escape variant	1·3

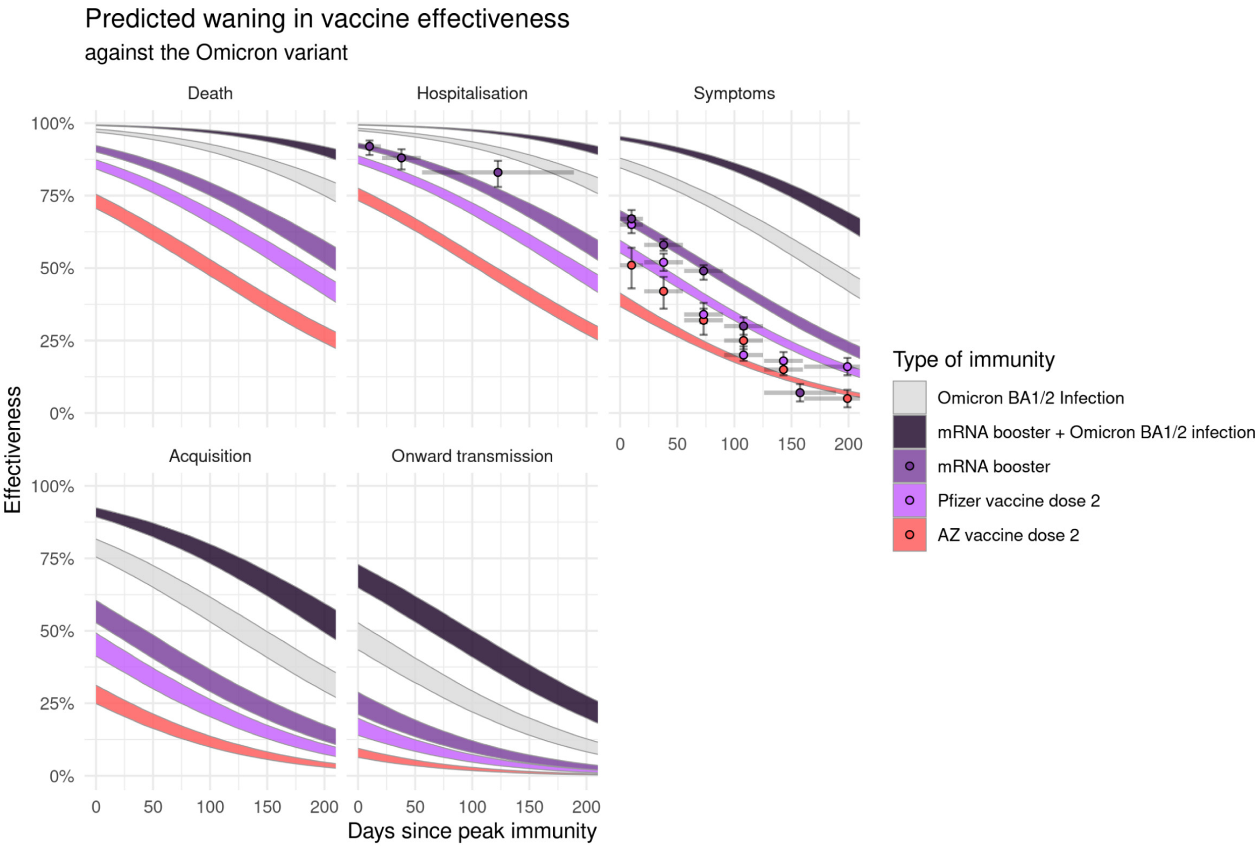

Figure A1: Predicted waning in vaccine effectiveness against the Omicron variant – Figure 4 from Hao et al. [6], reproduced under a Creative Commons license. Estimates of immune protection following peak immunity after a second dose of either AstraZeneca or Pfizer vaccines, after boosting with an mRNA vaccine, and/or Omicron infection. The dots indicate estimates of vaccine effectiveness from observation studies (with vertical 95% vaccine effectiveness confidence intervals and horizontal bars for the range of days since the immune event for individuals in those studies).

A.3 Probability of disease outcomes

We use protection-against-outcome values to calculate susceptibility to infection, probability of symptomatic disease, and probability of onward transmission given infection, probability of hospitalisation, probability of ICU admission, and probability of death. These are detailed in Table A2.

Table A2: Probability of disease outcomes parameter descriptions and equations. For further details about these equations and how they are derived, see [5].

Parameter definition	Notes
ξ_j, the susceptibility to infection of individual i: $$\xi_j = (1-\rho_\xi)\xi_i^0 \quad (6)$$	ρ_ξ is the protection against infection [Eq. (5)]; ξ_i^0 is the susceptibility of the ith individual if they were completely COVID naive (age-dependent, Tab. A1).
q_i, the probability that individual i becomes symptomatic: $$q_i = \frac{1-\rho_q}{1-\rho_\xi} q_i^0 \quad (7)$$	ρ_q is the protection against symptomatic infection [Eq. (5)]; ρ_ξ is the protection against infection [Eq. (5)]; q_i^0 is the probability of symptomatic infection for individual i if they were completely COVID naive (age-dependent, Tab. A1).
τ_i, the adjusted protection against onward transmission: $$\tau_i = \frac{s(1-\rho_\tau)(1+q_i^0)}{1+q_i}\beta_i, \quad (8)$$	$s = 0{\cdot}5$ for asymptomatic infection and $s = 1$ for symptomatic infection, ρ_τ is the unadjusted protection against onward transmission (calculated from Eq. (5)), β_i is the baseline infectiousness of the infector (age-dependent, Table A1), q_i^0 is the probability of symptomatic infection for individual i if they were completely COVID naive (age-dependent, Table A1), and q_i is probability of symptomatic disease defined later in Eq. (7).
$OR(p,r)$, an odds ratio, used to calculate an adjusted probability: $$OR(p,r) = \frac{\frac{rp}{1-p}}{1+\frac{rp}{1-p}} \quad (9)$$	r is the protection against disease outcome; p is a baseline probability of disease outcome (without protection).
$p^i_{H\|I}$, the probability of hospitalisation given infection for symptomatic individual i: $$p^i_{H\|I} = \frac{OR(p^0_{H\|E}, \rho_h(\alpha_i))}{q_i} \quad (10)$$	$p^0_{H\|E}$ is the baseline probability of hospitalisation given infection [Tab. A1]; $\rho_h(\alpha_i)$ is the protection against hospitalisation [Eq. (5)] that is dependent on α_i (the individual's neutralising antibody titre at the time of infection [Eq. (4)]); q_i is the probability of becoming symptomatic [Eq. (7)].
$p^i_{ICU\|H}$, the probability of requiring ICU if hospitalised: $$p^i_{ICU\|H} = \frac{OR(p^0_{ICU\|E}, \rho_h(\alpha_i))}{p^i_{H\|I} q_i} \quad (11)$$	$p^0_{ICU\|E}$ is the baseline probability of requiring the ICU given infection [Tab. A1]; ρ_h is the protection against hospitalisation [Eq. (5)] dependent on α_i (neutralising antibody titre at the time of infection [Eq. (4)]); $p^i_{H\|I}$ is probability of hospitalisation given infection for symptomatic individual [Eq. (10)]; q_i is the probability of becoming symptomatic [Eq. (7)].
$p^i_{H_D\|ICU^c}$, the probability of dying in the ward (and *not* having been admitted to ICU): $$p^i_{H_D\|ICU^c} = \frac{OR(p^0_{H_D\|E}, \rho_D(\alpha_i))}{(1-p^i_{ICU\|H})p^i_{H\|I} q_i} \quad (12)$$	$p^0_{H_D\|E}$ is the probability of death on ward (without visiting ICU) given infection [Tab. A1]; $\rho_D(\alpha_i)$ is the protection against death given infection [Eq. (5)] dependent on α_i (neutralising antibody titre at the time of infection [Eq. (4)]); $p^i_{ICU\|H}$ is the probability of requiring ICU [Eq. (11)]; $p^i_{H\|I}$ is the probability of being hospitalised [Eq. (10)]; q_i is the probability of becoming symptomatic [Eq. (7)].
$p^i_{ICU_D\|ICU}$, the probability of death in the ICU if in the ICU: $$p^i_{ICU_D\|ICU} = \frac{OR(p^0_{ICU_D\|E}, \rho_D(\alpha_i))}{p^i_{ICU\|H} p^i_{H\|I} q_i} \quad (13)$$	$p^0_{ICU_D\|E}$ is the baseline probability of dying in the ICU [Tab. A1]; $\rho_D(\alpha_i)$ is the protection against death given infection [Eq. (5)] dependent on α_i (neutralising antibody titre at the time of infection [Eq. (4)]); $p^i_{ICU\|H}$ is the probability of requiring ICU [Eq. (11)]; $p^i_{H\|I}$ is the probability of being hospitalised [Eq. (10)]; q_i is the probability of becoming symptomatic [Eq. (7)].

Parameter definition	Notes
$p^i_{W_D \mid ICU^c_D}$, the probability of death on the ward *after* leaving ICU without dying: $$p^i_{W_D \mid ICU^c_D} = \frac{OR(p^0_{W_D \mid E}, \rho_D(\alpha_i))}{(1 - p^i_{ICU_D \mid ICU}) p^i_{ICU \mid H} p^i_{H \mid I} q_i} \quad (14)$$	$p^0_{W_D \mid E}$ is the baseline probability of dying in the ward after returning from the ICU; $\rho_D(\alpha_i)$ is the protection against death given infection [Eq. (5)] dependent on α_i (neutralising antibody titre at the time of infection [Eq. (4)]); $p^i_{ICU_D \mid ICU}$ is the probability of death in the ICU if in the ICU [Eq.(13)]; $p^i_{ICU \mid H}$ is the probability of requiring ICU [Eq. (11)]; $p^i_{H \mid I}$ is the probability of being hospitalised [Eq. (10)]; q_i is the probability of becoming symptomatic [Eq. (7)].

B POPULATION TRANSMISSION MODEL

We use an agent-based model to model population transmission of SARS-CoV-2 (full model details can be found here: [5] and [7]). Within the model each agent in the simulation has an age, a corresponding age bracket (to determine their contacts using the contact matrix), a vaccination history and an infection history. At the start of the simulation, each person has zero neutralising antibodies. Then, during the simulation, agents will be vaccinated and can become sick and spread disease.

Infection spreads due to contact between infectious and susceptible individuals. The number of contacts made by an infectious individual is determined by supplying an age-stratified contact matrix (see Fig. B3). If contact j is susceptible, then the probability of infection is $\tau_i \xi_j$, where τ_i is the infectiousness of the infectious individual i [Eq. (8)], and ξ_j is the susceptibility of the contact j [Eq. (6)].

Once infected, an individual has probability q_i of being symptomatic (dependent on age and neutralising antibody titre, Eq. (7)). We also sample the time to becoming infectious, the time for symptom onset and the recovery time [9].

Each infection is logged with the individual's age, time of symptom onset, neutralising antibody titre at exposure, their vaccine status at exposure, the number of times that they had been infected, symptomatic/asymptomatic status, the number of individuals they infected, and the time they were isolated from the community. The resultant line list of infections is fed into the clinical pathways model to model clinical outcomes (Section C).

B.1 Scenario and input parameters

The transmission simulation takes in several scenario and input parameters. In our scenarios, we vary population type, immune escape, transmission potential (TP), vaccine coverage and boosting strategy. The broad timeline of the scenarios/simulations is shown in Figure B1.

Each scenario was run 1000 times, as we found that results comfortably converged by that point.

B.1.1 Population types

We considered "older" and "younger" populations, separating them based on *old-age dependency ratio*, or $OADR$, which is defined as

$$OADR = \frac{\text{population aged 65 and over}}{\text{population aged 20–64}} \times 100.$$

So, our "older" populations have an $OADR \geq 15$, while our "younger" populations have an $OADR \leq 12$. In practice, this means:

- **"Older" population** is representative of high-income countries (HIC) in the WPR. The "older" population distribution is averaged from China, Hong Kong SAR, Macao SAR, Japan, Republic of Korea, Singapore, Australia, New Zealand, New Caledonia, Guam, and French Polynesia;
- **"Younger" population** is representative of mostly lower-middle income and some upper-middle income countries (MIC) in the WPR. The "younger" population distribution is averaged from Mongolia, Brunei Darussalam, Cambodia, Lao People's Democratic Republic, Philippines, Fiji, Papua New Guinea, Solomon Islands, Vanuatu, Kiribati, Micronesia (Fed. States of), Samoa, and Tonga.

We acquired 2021 population data from the United Nations population database [10].

The older and younger population distributions are displayed in Figures B2(a) and (b) respectively. The older population demographic has more of the population aged around 20–64 years, while the younger population demographic skews downward and peaks at the 5–11 age group. For the simulations, we generated 100,000-person populations based on these distributions.

The older and younger populations in our model also have a different contact matrix describing social mixing (and thus infection spread) in the model (Figure B3).

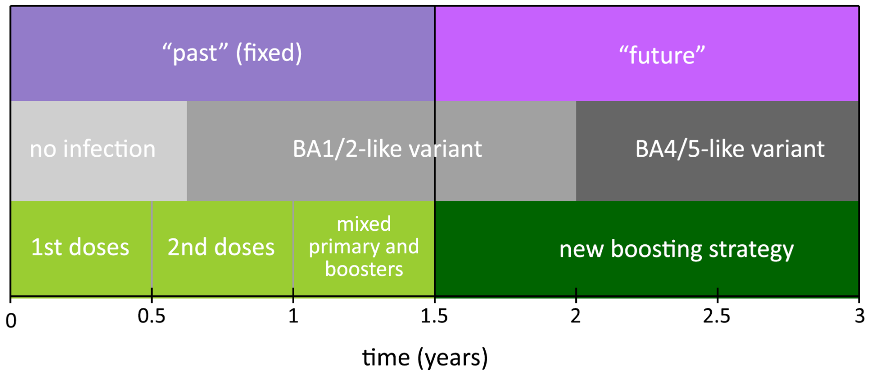

Figure B1: Broad timeline of the vaccination schedule and circulating variant. We consider the first 1·5 years to be the "past", and we are focused on multiple different strategies and scenarios in the "future" between 1·5–3 years. Note that we vary the precise timing of the new boosting strategy, and the time the BA4/5-like variant emerges in different scenarios.

To derive some exemplar contact matrices, we used aggregated contact matrices from "older" ($OADR \geq 15$) and "younger" ($OADR \leq 12$) countries that could be found on http://www.socialcontactdata.org/ [11, 12, 13, 14, 15, 16, 17, 18, 19, 20, 21]: for "older" countries we included Belgium, Finland, France, Germany, Hong Kong, Italy, Luxembourg, Poland; and "younger" included Vietnam and Zimbabwe. These countries fell into the appropriate *OADR* values and had contact matrix values for all the age-groups we required.

B.1.2 Transmission potential

Transmission potential (TP) reflects different populations' intrinsic transmission, which is dependent on a variety of factors such as demographics, weather/climate, housing, population density etc. In general, populations with high TP have a high past attack rate and populations with low TP have a low past attack rate.

We consider scenarios with:

- **High TP** (with mean attack rates around 80%–100% in the first 1·5 years depending on vaccination coverage); and
- **Low TP** (with mean attack rates around 15%–45% in the first 1·5 years depending on vaccination coverage).

B.1.3 Variants and immune escape

All scenarios start with a BA1/2-like variant, with an introduced immune escape variant (BA4/5-like) at a later time. When an immune escape variant emerges, all prior neutralising antibodies are suppressed relative to the new variant, representing the immune escape. The new circulating disease also has a higher transmission potential than the prior variant.

We consider immune escape variant introduction at either:

- **1.5 years**;
- **2.0 years**; or
- **2.5 years**

after the start of the main vaccination program.

B.1.4 Vaccination

Vaccination coverage

We consider three initial vaccination coverage levels, which are also depicted in Figure B2(c), (d) and (e) (for the younger population):

- **High coverage**: 80% primary vaccine coverage after the first year (88% primary vaccine coverage by 1·5 years) in both "older" and "younger" populations (equally 88002 vaccinated individuals in the older population, and 88000 vaccinated individuals in the younger population, with two primary doses);
- **Medium coverage**: 50% primary vaccine coverage (55% primary vaccine coverage by 1·5 years) in the "younger" population only (55000 vaccinated individuals with two primary doses); and
- **Low coverage**: 20% primary vaccine coverage (22% primary vaccine coverage by 1·5 years) in the "younger" population only (22001 vaccinated individuals with two primary doses).

Note that low vaccine coverage applies to a subset of "younger" populations where this scenario is relevant (such as Papua New Guinea and the Solomon Islands) — this is because countries with "older" populations tend to have high vaccination coverages, and exemplar low-vaccination coverage populations have younger population distributions.

Vaccination schedule in 0–1.5 years

The broad schedule in the first 1·5 years of our simulation is shown in Figure B1. Either 20%, 50%, or 80% total vaccination coverage (complete primary doses) is achieved by the end of the first year, with first doses given out in the first 6 months, and second doses given out in the second 6 months. During time 1–1·5 years, 80% of already vaccinated individuals are given boosters, while the remaining number of doses is given as primary course vaccinations.

The vaccine allocation broadly follows WHO guidelines, which recommends prioritising the vaccination of older and higher-risk groups. At the lower vaccination rate of 20%, we first allocate doses such that 80% of the 60+ age group are fully vaccinated by the end of the first two stages. At the higher vaccination rates of 50% and 80%, we allocate initial doses such that 95% of the 60+ age group are fully vaccinated by the end of the first two stages. The remaining available doses in the first two stages are then equally (proportionately) allocated to the 5–59 age groups.

Vaccination schedule in 1.5–3 years

Exploring different vaccination timings and targeting in the 1·5–3 year period is the main investigation of this paper, so we defer closer examination of these schedules to sections B.1.5, B.1.6, B.1.7.

Vaccine type

We assume that all primary doses are monovalent ChAdOx1 nCoV-19 (AstraZeneca) and all booster doses are monovalent BNT162b2 (Pfizer/BioNTech), unless otherwise noted (i.e. in the section comparing bivalent vaccination with monovalent vaccination).

B.1.5 High coverage boosting strategies

For the high coverage vaccination scenarios, we primarily consider three boosting strategies:

- **Pediatric boosting**;
- **High risk boosting**; and
- **Random boosting**.

We fix the number of vaccine doses (11, 000) in these scenarios, so that we can focus on the impact of vaccine allocation. 11, 000 doses is enough doses to boost approximately 80% of the 65+ age group in the older population (55+ age group in the younger population), or to boost approximately 80% of individuals aged 5–15 in the older population. Figure B4 shows the vaccine allocation across the populations for each boosting strategy.

Alongside the age-based boosting strategies, we also consider how timing affects the results. We consider two options, with boosters starting at either a fixed point (e.g. at 2 years) or delivered routinely (e.g. every 6 months). Each instance of a booster program takes approximately 3 months to administer.

B.1.6 Low coverage vaccination and boosting strategies

Here we consider scenarios for younger populations with low or medium vaccination coverage. We have three different vaccination/boosting strategies targeting different groups:

- New pediatric primary vaccination,
- High risk boosting (older first),
- New general (random) primary vaccinations.

In the low and medium vaccination coverage scenarios, there is typically *insufficient* numbers of vaccinated individuals to be given a booster dose (all 11,000 of them). Because there is a large number of individuals still unvaccinated, we consider two new primary vaccination programs, one to children, and one to the general population. There are still 11,000 total doses, meaning that 5,500 individuals will be fully vaccinated in these new vaccination programs.

B.1.7 Boosting strategies for the age-cutoff-investigation

In the previous strategies, we typically defined "high risk boosting" as boosting to the 65+ age groups within the older population, and to the 55+ age groups within the younger population. Here we consider what would happen if we systematically reduce this age threshold (i.e., increasing booster eligibility to increasingly younger age groups), to test the limits of cost effectiveness of coverage. In order to have high booster coverage, we need high primary vaccination coverage. As such, we only consider the high-coverage (80%) scenarios, in both older and younger population settings.

We considered seven boosting strategies:

- **65+ years boosting**,
- **55+ years boosting**,
- **45+ years boosting**,
- **35+ years boosting**,
- **25+ years boosting**,
- **16+ years boosting**,
- **5+ boosting**.

There is a fixed percentage of booster uptake: 80% of eligible individuals (who have had a primary series vaccination) receive a booster. This means that the number of booster doses given out increases as the eligibility age decreases. Since the younger population and older population have different age distributions, the number of doses changing per 'step' will be different between the two.

Note that the 65+ years scenario that we test here is not the same as the prior "further boosting high risk" scenarios in the rest of the report, where a fixed number of doses (11,000) were administered. Here, we administer sufficient doses to achieve 80% booster uptake, which is not exactly equal to 11,000.

B.1.8 Bivalent boosting

Bivalent boosting is expected to have a greater impact in populations with low levels of vaccination [22]. Hence we only explored low- and medium-coverage vaccination settings in younger populations. Khoury et al [22] found that bivalent vaccines, on average, produced 1·61-fold higher titers than monovalent vaccines. We implement bivalent boosters within the model by using this average multiplier (1·61) on top of the Pfizer booster neutralisation values for any boosters given out during the 1·5–3 year stage as part of high-risk boosting.

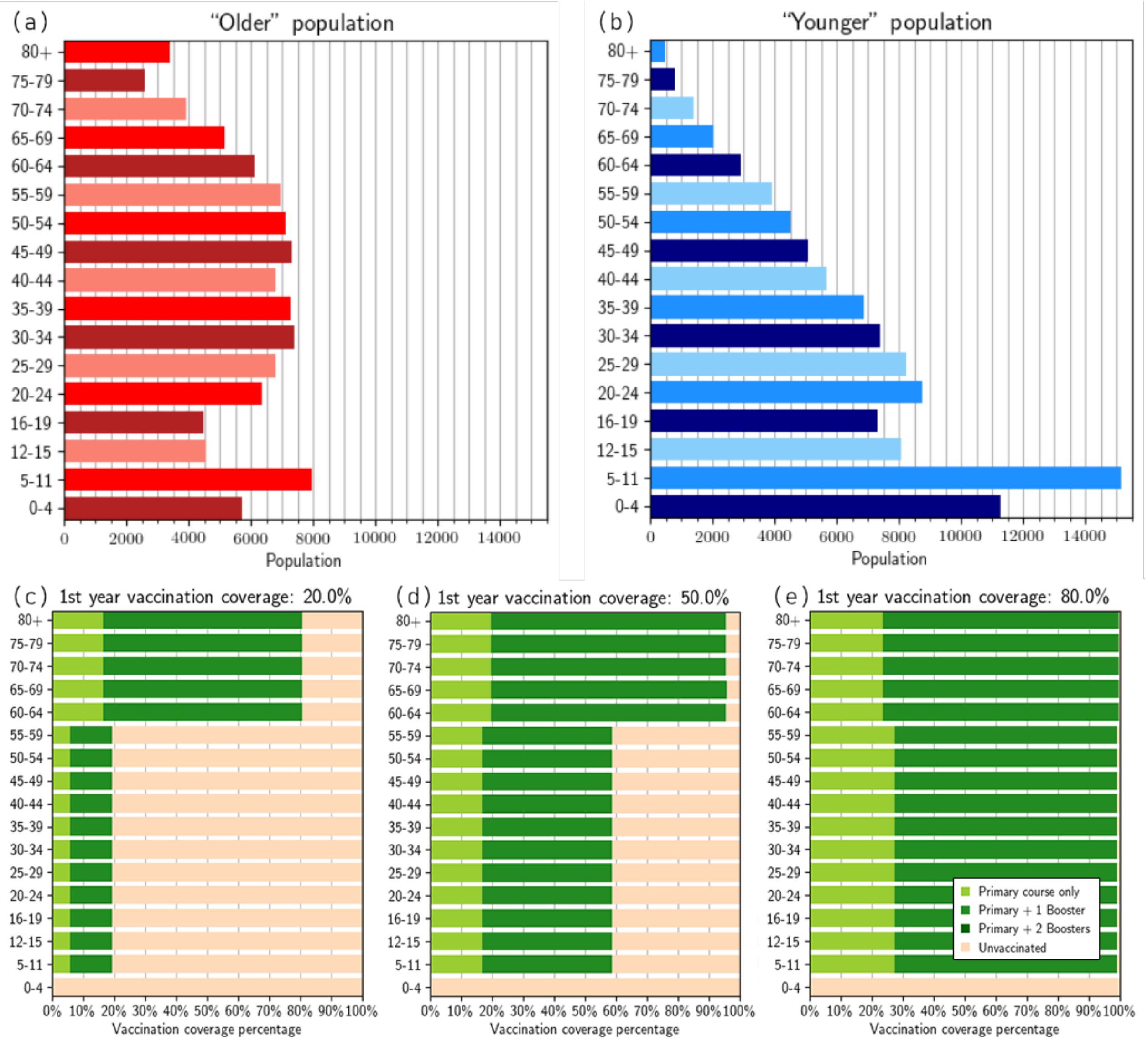

Figure B2: Population demographics (100,000 individuals) and initial vaccination coverages. (a) “older” population distribution; (b) “younger” population distribution; (c) low initial vaccination coverage* after 1·5 years, (d) medium initial vaccination coverage* after 1·5 years, (e) high initial vaccination coverage* after 1·5 years. *Note: proportions are for the younger population, but it is very similar for the older population.

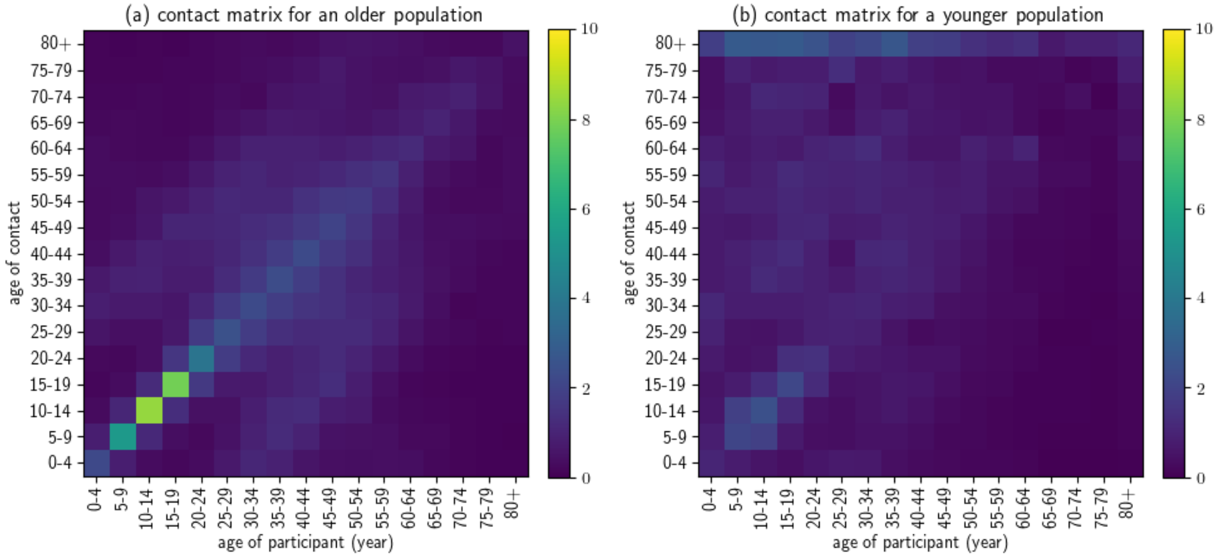

Figure B3: Contact matrices used for: (a) "older" population, (b) "younger" population.

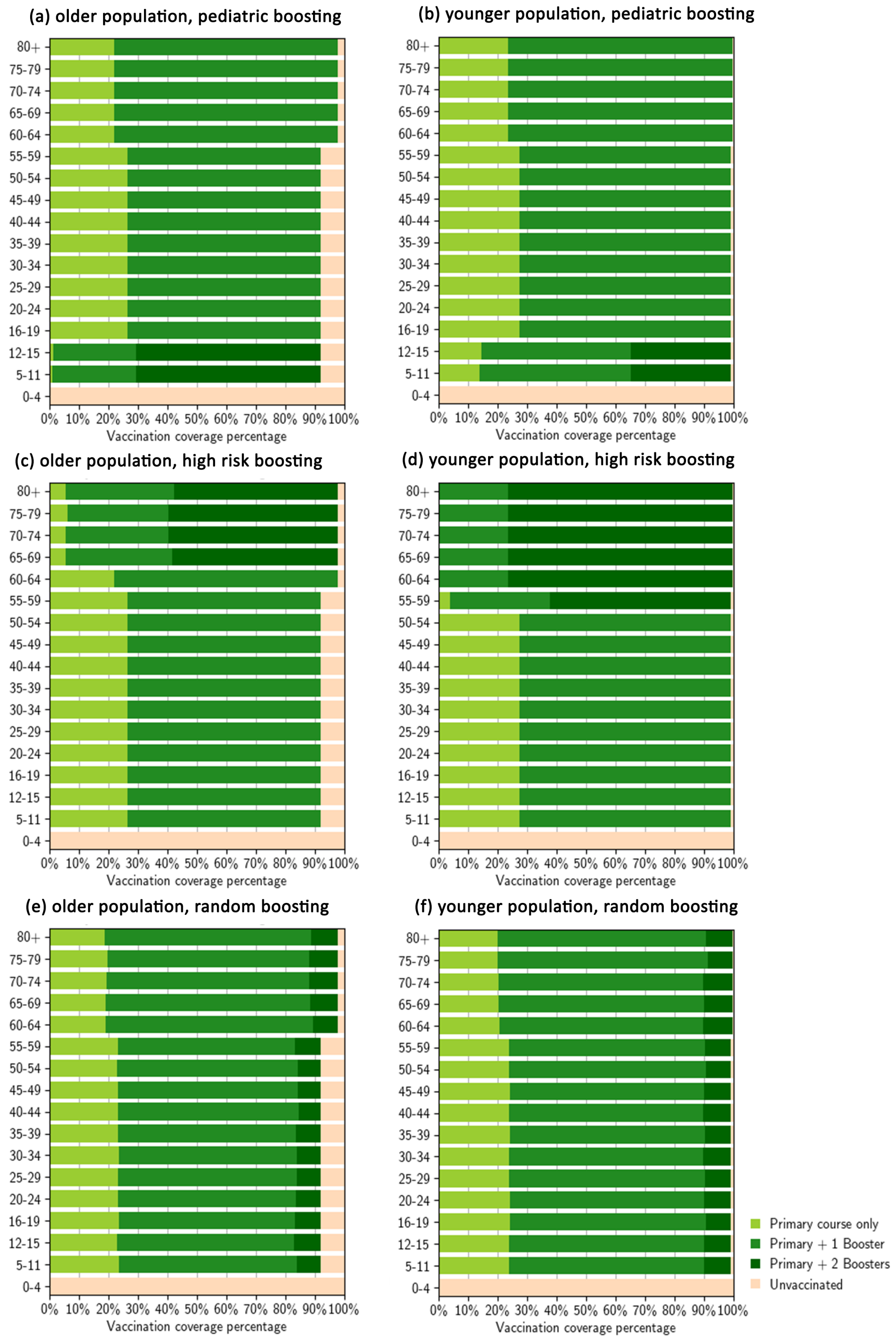

Figure B4: Vaccination coverage distribution after different high-coverage boosting allocations in the older and younger populations.

C CLINICAL PATHWAYS

In the clinical pathways model, we generate a clinical timeline for every symptomatic individual; the neutralising antibodies of each individual affects the pathway taken. The model is from Conway et al. [5], which in turn is based on the model by Knock et al. [23] and is an extension of the work done in [7, 24]. See Figure C1 for a schematic of the clinical pathways model. See Section A for all the definitions of the transition probabilities for the clinical pathways. See [5] for further details.

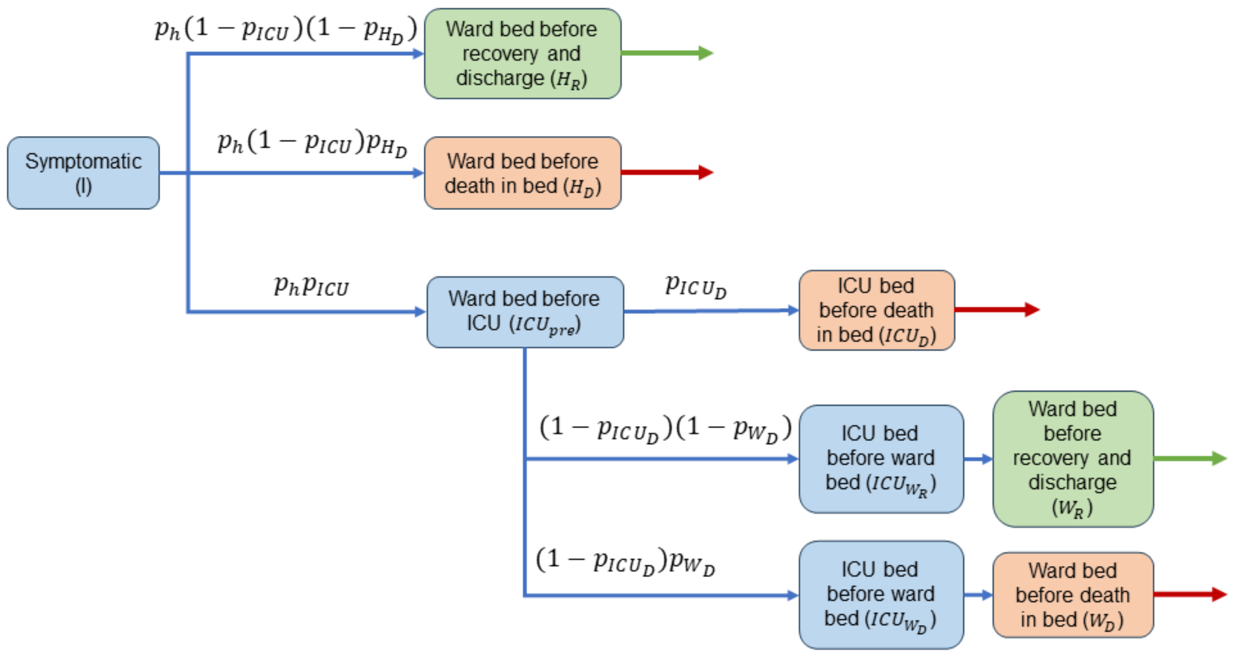

Figure C1: Depiction of the compartmental structure of the clinical pathways model. Note that probabilities are implicitly conditional. Adapted from [5, 7].

Each symptomatic individual has probability $p^i_{H|I}$ of being hospitalised given symptomatic infection [Eq. (10)].

Once hospitalised, an individual can (1) recover and be discharged from a ward bed (H_R), (2) die in a ward bed (H_D), or (3) move to an ICU bed (ICU_{pre}), with the following probabilities:

$$H_R : (1 - p^i_{ICU|H})(1 - p^i_{H_D|ICU^c}), \quad H_D : (1 - p^i_{ICU|H})p^i_{H_D|ICU^c}, \quad ICU_{pre} : p^i_{ICU|H}, \tag{15}$$

where $p^i_{ICU|H}$ is the probability of being admitted to ICU given hospitalisation, $p^i_{H_D|\text{ICU}^c}$ is the probability that individual dies on ward given that they are in hospitalised and are not going to ICU (see Table A2).

If individual is admitted to ICU, then they can: (1) die in the ICU (ICU_D), (2) die in a ward bed after leaving the ICU (ICU_{WD}), or (3) recover and be discharged from a ward bed after leaving the ICU (ICU_{WR}), with the following probabilities:

$$ICU_D : p^i_{ICU_D|ICU}, \quad ICU_{WD} : (1 - p^i_{ICU_D|ICU} p^i_{W_D|ICU^c_D}), \quad ICU_{WR} : (1 - p^i_{ICU_D|ICU})(1 - p^i_{W_D|ICU^c_D}), \tag{16}$$

where $p^i_{ICU_D|ICU}$ is the probability that individual i dies in the ICU given ICU admittance, and $p^i_{WD|ICU^c_D}$ is the probability that individual of dies in a ward bed after leaving ICU without dying (see Table A2). If an individual transitions into ICU_{WR} or ICU_{WD}, they will then move into W_R or W_D, where they will either recover or die respectively.

Note that the length of stay for each individual in each compartment is informed by the Australian Delta wave [25] (we assume it does not depend on neutralising antibody titre levels).

D COST-EFFECTIVENESS ANALYSIS

D.1 CEA model overview

The cost-effectiveness model uses as inputs the outputs from the clinical pathways model linked to the mechanical agent-based model (ABM) that in turn, is linked to the infection transmission/dynamics model. A population of 100,000 is run through the models, over a three-year period. The first 1·5 years represents the main vaccination program (also referred to as prior or primary vaccination). The second 1·5 years represents the different boosting programs that are considered for the cost-effectiveness analysis, representing different emergence times, transmissibility, boosting times, and boosting frequency. The ABM and clinical pathways models provide scenario-specific mean estimates of vaccination doses delivered per 100,000 people, COVID-19 infections (all, symptomatic, hospital admissions and total hospital bed days occupied, intensive care unit (ICU) admissions and total ICU beds occupied), and COVID-19 related deaths by 10-year age groups.

The cost-effectiveness analysis has been conducted from the healthcare system perspective, including direct medical costs only. The main categories of costs included are (1) programmatic costs related to the vaccination intervention, including vaccine dose costs, wastage, and delivery costs; and (2) disease management costs at home, in outpatient and inpatient settings for symptomatic COVID-19 related illness. COVID-19 testing costs were not included. While these costs have been estimated to be substantial [26], they remain highly uncertain. In any resurgence, we estimate a much broader use of rapid antigen testing than polymerase chain reaction (PCR) for case ascertainment; thus, the historical use of testing strategies cannot inform future testing use. Furthermore, PCR capacity varies dramatically by country, and the use of different types of tests will likely vary by case numbers.

D.2 Defining exemplar country contexts for cost-effectiveness analysis

All countries in the WPR started COVID-19 vaccination programs in 2021. While countries had different vaccination strategies, in general first doses were assigned to frontline workers, at risk adults and the elderly, followed by the remaining adult population. Programs were expanded to include children aged 12 and above starting in early 2022. Most countries further expanded their vaccine policy to include children 5 years and older in mid-2022.

According to WHO data, 2-dose vaccine coverage varies significantly throughout the Western Pacific (Tables D1 and D2). High income, 'older' demographic countries tend to have higher vaccination coverage, ranging from 64·5% in New Caledonia to 87·4% in Singapore with a median of 84·5% in New Zealand (as a proportion of total population as of 22/12/22). Lower-middle and upper-middle income countries with younger demographics displayed a much wider range of vaccination coverage ranging from 3·6% in Papua New Guinea (PNG) to 101·9% in Brunei with a median coverage 67·4% in The Philippines. Booster coverage displayed a similar pattern.

In alignment with the two ABM populations, representing differing demographics within the WPR, we consider three key groupings of 'exemplar' countries in terms of: (1) demography (typical 'older' versus 'younger' population demographics); (2) health systems capacity and prior primary COVID-19 vaccine coverage rates (strong health systems and high prior primary vaccine coverage versus relatively weaker health systems and lower prior primary vaccine coverage); (3) income group level (high income versus upper-middle and lower-middle income); and (4) vaccination delivery unit costs and disease management costs.

The representative 'exemplar' countries groupings are as follows, with full details provided below (Table D1):

- **Group A**: High income country (HIC), 'older' population with strong health systems capacity and high (∼80%) prior primary vaccine coverage. High unit costs for vaccine delivery and disease management. (Countries in this group include Japan, Australia, Republic of Korea, and Hong Kong, and are representative of other high income countries in the WPR such as New Zealand)
- **Group B**: Upper- and lower- middle income country (MIC), 'younger' population with varying levels health systems capacity and prior primary vaccine coverage (∼80% and ∼50%). Low-to-high unit costs for vaccine delivery (depending on geography and population size) and low-to-middle unit costs for disease management. (Countries in this group include Fiji, Samoa, Tonga, Mongolia, Cambodia, Philippines, Lao, Vanuatu, Kiribati, Micronesia, PNG, and Solomon Islands)
- **Group C**: Lower-middle income country, younger population with weaker health systems capacity and low

(∼20%) prior primary vaccine coverage. Low unit costs for vaccine delivery and disease management. (Countries in this group, a subgroup of Group B, include PNG and Solomon Islands)

Some WPR countries are not included in these representative 'exemplar' country groupings (for example, those with demographics that classify as neither 'older' nor 'younger', or those with demographics that match to 'older' or 'younger' categorisation, but per-capita income level does not). The implications for these countries would need to be considered in light of the findings for **Groups A** and **B**.

Table D1: Countries and areas in WHO Western Pacific Region (WPR) and characteristics.

Country[1]	Income classification[2]	Younger/ Older	Population size[3]	2 dose vaccination coverage[4]	Booster coverage[4]
American Samoa	Upper middle	-	45,035	75·05%	43·77%
Australia	High	Older	25,688,079	84·92%	56·06%
Brunei Darussalam	High	Younger	445,373	101·93%	77·49%
Cambodia	Lower middle	Younger	16,589,023	87·33%	62·21%
China	Upper middle	Older	1,412,360,000	86·82%	54·7%
Cook Islands	-	-	17,604*	83·56%	30·38%
Fiji	Upper middle	Younger	924,610	71·33%	18·78%
French Polynesia	High	Older	304,032	66·23%	39·95%
Guam (USA)	High	Older	170,534	83·55%	42·74%
Hong Kong	High	Older	7,413,100	-	-
Japan	High	Older	125,681,593	81·43%	66·67%
Kiribati	Lower middle	Younger	128,874	61·86%	19·61%
Lao PDR	Lower middle	Younger	7,425,057	74·54%	27·86%
Macao SAR	High	Older	686,607		
Malaysia	Upper middle	-	33,573,874	85·06%	50·28%
Marshall Islands	Upper middle	-	42,050	61·89%	36·41%
Micronesia, Federates States of	Lower middle	Younger	113,131	57·53%	26·55%
Mongolia	Lower middle	Younger	3,347,782	66·64%	32·25%
Nauru	High	-	12,511	79·24%	46·75%
New Caledonia	High	Older	271,030	64·49%	32·96%
New Zealand	High	Older	5,122,600	84·87%	56·33%
Niue	-	-	1,653*	100·99%	75·65%
Northern Mariana Islands Commonwealth of the USA	High	-	49,481	78·2%	42·11%
Palau	Upper middle	-	18,024	101·07%	71·11%
Papua New Guinea	Lower middle	Younger	9,949,437	3·46%	0·36%
Philippines	Lower middle	Younger	113,880,328	67·42%	19·38%
Pitcairn Island	-	-	50*	74%	46%
Republic of Korea	High	Older	51,744,876	87·17%	65·63%
Samoa	Lower middle	Younger	218,764	89·54%	39·85%
Singapore	High	Older	5,453,566	87·4%	77·34%
Solomon Islands	Lower middle	Younger	707,851	31·68%	2·54%
Tokelau	-	-	1,399*	163·19%	71·7%
Tonga	Upper middle	Younger	106,017	72·72%	36·24%
Tuvalu	Upper middle	-	11,204	79·05%	46·88%
Vanuatu	Lower middle	Younger	319,137	42·75%	5·39%
Vietnam	Lower middle	-	97,468,029	87·89%	59%
Wallis and Futuna	-	-	10,749*	62·15%	28·46%

[1] Abbreviations: PDR, People's Democratic Republic; SAR, Special Administrative Region; USA, United States of America
[2] Income Classification data sourced from World Bank 22/12/22
[3] Population data from Our World in Data 22/12/22
[4] Data retrieved from WHO Coronavirus (COVID-19) Dashboard 22/12/22. Data on 2-dose coverage and booster coverage are estimated based on number of doses administered and total population size
[*] Data not available from Our World in Data sourced from Worldometer

In Table D1, Group A is marked in green, representing older populations with high income, strong health systems capacity and high prior primary coverage. Meanwhile, Group B, marked in yellow, represent younger populations with upper- and lower-middle incomes, middle-to-strong health systems capacity and middle-to-high prior primary vaccine coverage. Finally, Group C are marked in blue, represent younger populations with lower-middle income, weak health

systems capacity and lower prior primary vaccine coverage. There are several countries that are not highlighted—these were not included in these representative 'exemplar' country groupings. The implications for these countries would need to be considered in light of the findings for Groups A and B, as they would likely sit somewhere in between these groupings.

Table D2: Median vaccination coverage by age demographic in the Western Pacific Region.

Age Demographic	Median 2-dose coverage*	IQR	Range
Younger	67·42%	57·53%–74·54%	3·46%–101·93%
Older	84·87%	81·43%–86·82%	64·49%–87·4%

* as % of total population, as of 22/12/22

D.3 Resource use and costs

Inputs and data sources for estimating costs of COVID-19 vaccination and disease management are presented in Table D4.

D.3.1 COVID-19 vaccine dose cost

COVID-19 vaccine price data were retrieved from the WHO COVID-19 vaccine price report [27]. This report summarizes vaccine dose price data based on the WHO MI4A COVID-19 Vaccine Purchase Database [28], which includes vaccine purchase data from public sources and data reported by countries through the WHO/UNICEF Joint Reporting Form (eJRF). Countries' names are not available in the dataset; however, the WHO region and income level are provided. Few countries in the Western Pacific Region (WPRO) had available price data, so our study has used global pricing data, by income group. Though AstraZeneca is no longer a preferred vaccine, we have included it in the economic model as it was used widely in the Western Pacific region in 2021 and has the lowest price per dose across all vaccines.

The vaccine dose price used in the base case was the average price per dose for all vaccines: Pfizer BioNTech (Comirnaty), Moderna (mRNA-1273), Janssen (Ad26.COV 2-S), and AstraZeneca (Vaxzevria) by income group. For groups A and C, the ranges were the minimum and maximum prices for high- and lower-middle-income countries respectively. Group B comprises a mix of lower- and upper-middle income countries, so the range was obtained from the minimum and maximum vaccine prices of lower- and upper-middle countries combined (Table D4 and Table D3).

Table D3: Average price per vaccine (per dose, US$), by income group from WHO vaccine price report. Data from public sources and as reported by countries to WHO, up to March 2022.

Income group	All vaccines	Pfizer	Moderna	Janssen	AstraZeneca
High income	$14·2	$20·7	$25·2	$9·7	$3·9
Upper-middle income	$10·3	$12·5	$10·0	$7·5	$4·0
Lower-middle income	$7·8	$10·0	$10·0	$7·5	$4·1

Table D4: Inputs for estimating COVID-19 vaccination and disease management costs.

Parameter	Base case (range)	Source of data and rationale
Cost per dose of vaccine ($)		
Group A (all high income)	14·2 (3·9–25·2)	Data from WHO COVID-19 vaccine price report [27]. Base case is mean by income group across vaccines; range is the minimum and maximum by vaccine type.
Group B (all middle income)	7·8 (4·0–12·5)	As for Group A
Group C (middle income, low cov)	7·8 (3·9–10·0)	As for Group A
Delivery cost per dose ($)		
Group A (80% coverage)	23·1 (11·1–33·6)	Data from government reports [29, 30] or studies [31, 32]. Base case is average across Group A countries with cost data; range is minimum and maximum of costs.
Group B (50 or 80% coverage)	9·8 (0·7–19·1)	Data from UNICEF reports [33, 34]. Base case assumes the cost at 70% coverage; range is minimum and maximum of costs
Group C (20% coverage)	7·7 (2·5–10·5)	Data from UNICEF reports [33, 34]. Base case assumes double the cost at 70% coverage; range is minimum and maximum of costs
Proportion of doses wasted		
Group A (all high income)	10% (0–20)	Data from UNICEF reports [33, 34]. Assumes range from no dose wasted to double the proportion of doses wasted.
Group B (all middle income)	10% (0–20)	As for Group A
Group C (middle income, low cov)	10% (0–20)	As for Group A
Cost for non-hospitalized case ($)[1]		
Group A (all high income)	75 (53–115)	Data from medical fee schedules [35, 36, 37, 38], studies [39], or WHO-CHOICE [40]. Base case is average of available cost data; usual range estimation above.
Group B (all middle income)	52 (21–131)	Data from Torres-Rueda, et al. [26]
Group C (middle income, low cov)	31 (28–35)	Data from Torres-Rueda, et al. [26]
Cost for hospitalization without ICU, per day($)		
Group A (all high income)	351 (209–657)	Data from medical fee schedules [35, 36, 37, 38], studies [39], or WHO-CHOICE [40]. Base case is average of available cost data; usual range estimation above.
Group B (all middle income)	52 (34–99)	Data from Torres-Rueda, et al. [26]
Group C (middle income, low cov)	41 (40–42)	Data from Torres-Rueda, et al. [26]
Cost for hospitalization needing ICU, per day($)[2]		
Group A (all high income)	2594 (825–4284)	Data from medical fee schedules [35, 36, 37, 38], studies [39], or WHO-CHOICE [40]. Base case is average of available cost data; usual range estimation above.
Group B (all middle income)	543 (295–1273)	Data from Torres-Rueda, et al. [26]
Group C (middle income, low cov)	347 (341–353)	Data from Torres-Rueda, et al. [26]
Cost per COVID-related death ($)		
Body bag	65 (fixed)	Data from Torres-Rueda, et al. [26]

[1] Cost for non-hospitalized case includes two visits to a clinic.
[2] Costs of hospitalized critical case from original report included both ICU and non-ICU bed days and thus have been inflated by 20% to represent the cost of an ICU bed day alone.

D.3.2 COVID-19 vaccine delivery cost

The cost of COVID-19 vaccine delivery remains uncertain. Delivery costs will vary by vaccine type (including cold chain requirements), country health systems capacity, delivery mechanism and target population and coverage level. In most scenarios explored, a set number of doses (11,000) are delivered to a population of 100,000, equivalent to a coverage of 11% of the total population. While the different scenarios explored (high risk versus random versus paediatric boosting) likely have different delivery costs associated with them, given the underlying uncertainty, we assume the same delivery costs across all scenarios. Delivery costs are generally assumed to be U-shaped, decreasing as coverage increases due to shared fixed costs across a larger population being vaccinated, and increasing at very high coverage levels due to difficulties in vaccinating hard-to-reach populations. For Groups A and B countries, given high

to moderate prior vaccination coverage, we assume booster doses would be delivered at the same unit cost per dose as the primary doses. We assume across all scenarios that delivery of booster doses even at low coverage levels, would incur the same cost of vaccine delivery as the primary doses.

Group A ('older' population, high income countries)

There are no consistent estimates of vaccine delivery costs for high-income countries. We sought to estimate or find COVID-19 vaccination delivery costs for a select number of countries in Group A where data were available, to use as inputs for the modelling. Delivery costs per dose for Hong Kong and Korea were taken from previous publications with assumed COVID-19 vaccination coverage rates of 72% and 80%, respectively [31, 32]. Delivery costs per dose for Japan were taken from the Japanese Government's National Treasury's burden for the vaccination measures against the COVID-19 report, at an unspecified coverage rate [29]. Delivery costs for Australia were calculated by dividing the Australian government's reported funding for COVID-19 vaccine distribution and administration in 2020–2022 by the total doses administrated up to mid-2022 (about 80% coverage) [30]. These unit delivery costs were used for the 80% and 50% coverage scenarios and multiplied by two to estimate the delivery costs at 20% coverage. We use the average cost across all estimates obtained for the base case delivery cost, and the minimum and maximum delivery cost estimates as upper and lower-bound ranges.

Group B and C ('younger' population, middle-income countries)

The COVID-19 vaccine delivery cost estimates used in modelling the 'younger' demographic populations were based on two recent UNICEF reports that provided estimates for low- and middle-income countries (LMICs) [33, 34]. These delivery costs refer to the costs associated with delivering vaccines to target populations exclusive of vaccine purchase costs. The costs estimated in both reports are financial costs, including (1) variable costs (e.g., cold chain equipment, per diem for outreach, personal protective equipment, vaccine transport, and management, etc.) and (2) fixed costs (i.e., handwash station, training, planning and coordination, social mobilization, pharmacovigilance, behavioural and social data collection). For this study, we assumed the economic costs required for a cost-effectiveness analysis, would be similar to the financial costs, and therefore used these estimates in the base case.

In the latest UNICEF report, COVID-19 vaccine delivery costs were estimated for countries achieving a 70% of total population coverage (equivalent to 92% coverage rate in population ≥12 years of age) in four different scenarios (leveraging fixed delivery sites, balancing human resource protection, protecting human resources partially, protecting human resources fully) [34]. In the earlier report, which focussed on achieving 20% coverage, the estimation was based on the leveraging fixed delivery sites scenario only. For consistency, we have chosen the delivery costs under this scenario as the base case, which assumed 10% of the available workforce allocated to delivery, 85% fixed site delivery, and 15% outreach delivery. The fixed-outreach proportion was close to the data for the Western Pacific region in 85 National Deployment and Vaccination Plans (86%–14%) [34]. In the leveraging scenario from the earlier UNICEF report, the average cost per dose delivery at 20% coverage was approximately double that of achieving 70% coverage, as fewer people shared fixed costs. Due to the lack of country-specific estimates at 20% coverage, we also assumed that the delivery costs at 20% coverage were double those at 70% coverage. We also assumed that the 50% and 85% coverage scenarios had the same unit delivery costs as the 70% coverage scenario. Delivery cost estimates used in the model are provided in Table D5.

Table D5: Vaccination delivery cost estimates (2020 USD) by country and initial primary vaccination coverage.

Country	Group	20% Coverage	50% Coverage[1]	70% Coverage
Australia	A	-	-	$22·30[2]
Japan	A	-	-	$33·60
Korea, Rep.	A	-	-	$11·10
Hong Kong	A	-	-	$25·30
Fiji	B	-	*	$10·50
Samoa	B	-	*	$17·80
Tonga	B	-	*	$19·10
Mongolia	B	-	*	$13·40
Cambodia	B	-	*	Not available[3]
Lao PDR	B	-	*	$1·30
Philippines	B	-	*	$0·70
Vanuatu	B	-	*	$5·70
Kiribati	B	-	*	$29·30[4]
Micronesia, Fed. Sts.	B	-	*	$23·10[4]
Papua New Guinea	B & C	$5·00[5]	-	$2·50
Solomon Islands	B & C	$10·50[5]	-	$5·20

[1] Entries marked with * are assumed to be the same as the 70% coverage.
[2] Australia 2020–2022 vaccine program budget (including vaccine program implementation, administration and distribution) divided by total administrated doses up to mid-2022 = 1876·7 million /57·92 million $\sim$ 32·4 AUD $\sim$ 22·3 USD. Budget data from Minister for Finance of the Commonwealth of Australia; doses administration data from Our World in data 01/07/2022.
[3] Cambodia delivery costs were not available in UNICEF report.
[4] Costs of delivery for Micronesia and Kiribati were excluded from the mean delivery cost estimate for the Group B, younger, populations, due to their small population size ($\sim$125,000) and high delivery costs.
[5] Assumed to be 2× delivery cost estimates at 70% coverage, based on UNICEF report 2021–2022 in which average delivery cost across LMICs at 20% coverage was double the cost at 70% coverage in leveraging fixed delivery sites scenario.

D.3.3 COVID-19 treatment cost

Group A ('older' population, high income countries)

Detailed cost estimates for management of SARS-CoV-2 infections were estimated for a small number of countries in Group A, where these were readily available. Based on costing methods by Torres-Rueda et al. [26] as described below and used for middle-income countries (Groups B and C), we used the Australian medical fee schedules and publicly available government data to calculate the three types of case management costs [36, 38]. We used the same method to estimate the case management costs in Japan by applying the Japanese medical fee schedule [35]. Of note, in the home-based cases in HICs, we have excluded the home-based bed-day cost due to lack of detailed costing method in the reference article. Also, in hospital-based critical cases, we dropped the general ward bed-day input and changed the number of units per input for ICU bed-day from 0.66 to 1. Malaria testing was included in all LMICs hospital-based cases, but we removed it from the costs for HICs, given that HICs are predominantly low-prevalence malaria regions where this testing may not be a routine admission test. The costs of inpatient cases in Hong Kong were taken from a cost-effectiveness study of the COVID-19 vaccine in Hong Kong, with the source being the public charges for non-eligible persons [32, 37]. The costs of hospitalisation cases in Korea were obtained from a COVID-19 cost-effectiveness analysis in Korea, which employed the cost estimations by Korea Disease Control and Prevention Agency [39]. The outpatient costs for home-based care in Korea were based on WHO CHOICE unit costs [40], which we adjusted for inflation and currency conversion.The cost per COVID-19 related death only includes the cost of a body bag based on the study by Torres-Rueda et al. [26], and thus is likely to be underestimated.

Group B and C ('younger' population, middle-income countries)

All disease costs for LMICs were available directly from a model-based cost estimations study [26]. The study used data from three LMICs (Ethiopia, Pakistan, and South Africa) as the model references to extrapolate the case management costs for home-based care, hospitalisation for severe care, and critical care across all LMICs. The original costs

reported in the study were inflated to 2020 USD.

Home-based care costs are defined as the cost per mild-to-severe case requiring home-based care, including (1) the cost of home-based care bed-day; (2) the cost of community-based care via a clinician's visit. The number of bed-day and clinician visit was set at 5 and 2, respectively.

Hospitalised severe care costs were calculated per case and per day, including (1) general ward bed-day; (2) diagnostics. Hospitalised critical care costs were also presented per case and per day. Compared with severe cases, the additional costs per case per day were: (1) ICU bed-day; (2) additional resourcing per COVID-related complication. However, as the modelled epidemiological data is presented by ICU admission (rather than combining a patient who has received ICU and general ward care) the cost shown in this report likely underestimates the actual cost per day of a patient treated in an ICU. As general ward costs were considered representative of one-third of the bed day costs, we conservatively inflated the bed day cost by 20% when applying these costs in the economic model. Further clarification is presented in Tables D6 and D7.

Table D6: Disease management unit costs (2020 USD) by country.

Country	Group	Non-hospitalised, per case	Hospitalised without ICU, per day	Hospitalised with ICU, per day	Death, per case
Australia	A	$53·50	$271·60	$4284·30	$ 64·50
Japan	A	$54·00	$208·70	$2120·50	$64·50
Korea, Rep.	A	$76·80	$267·00	$825·00	$64·50
Hong Kong	A	$114·70	$657·20	$3144·30	$64·50
Fiji	B	$131·10	$99·00	$1272·70	$64·50
Philippines	B	$47·00	$48·80	$410·70	$64·50
Samoa	B	$82·10	$73·10	$1,005.90	$64·50
Tonga	B	$79·00	$70·20	$964·30	$64·50
Mongolia	B	$59·80	$53·00	$441·80	$64·50
Cambodia	B	$22·90	$35·90	$308·20	$64·50
Lao PDR	B	$37·30	$42·80	$362·80	$64·50
Vanuatu	B	$37·90	$43·90	$370·80	$64·50
Kiribati	B	$20·80	$34·30	$295·10	$64·50
Micronesia, Fed. Sts.	B	$42·60	$46·10	$388·30	$64·50
Papua New Guinea	C	$34·80	$41·70	$353·50	$64·50
Solomon Islands	C	$28·00	$39·90	$340·90	$64·50

Table D7: Unit inputs and unit costs (2020 USD) for Japan and Australia.

	Number of units per input	Japan	Australia
Home-based care	**per case**		
Community-based care via clinical visit	2	$27·00	$26·70
Total		$54·00	$53·50
Hospital-based (severe)	**per case/day**		
Inpatient ward bed-day (severe)	1	$196·70	$264·10
Chest X-ray	0·125	$19·70	$24·80
Full blood count (including haemoglobin test)	0·125	$27·70	$11·70
Blood urea and electrolyte test (including C-reactive protein test)	0·125	$22·20	$12·20
HIV test	0·125	$26·20	$10·80
Total		$208·70	$271·60
Hospital-based (critical)	**per case/day**		
ICU bed-day	1	$1359·10	$3733·80
Chest X-ray	10	$19·70	$24·80
Full blood count (including haemoglobin test)	10	$27·70	11·70
Blood urea and electrolyte test (including C-reactive protein test)	10	$22·20	$12·20
Venous blood gas test	10	$4·20	$4·20
HIV test	0·1	$26·20	$10·80
Acute respiratory distress syndrome	0·47	$22·50	$22·50
Acute kidney injury days	0·04	$10·60	$10·60

Acute cardiac injury days	0·06	$46·30	$46·30
Liver dysfunction days	0·06	$89·30	$89·30
Pneumothorax days	0·01	$7·00	$7·00
Hospital-acquired pneumonia days	0·05	$18·90	$18·90
Bacteraemia days	0·01	$32·60	$32·60
Urinary tract infection days	0·01	$9·00	$9·00
Septic shock days	0·05	$0·80	$0·80
Total		$2120·50	$4284·30

D.4 Health Outcomes

Health outcomes were presented as disability-adjusted life-years (DALYs) for each modelled scenario. DALYs were calculated as the sum of years of life lost (YLLs) and years lived with disability (YLDs).

Years of life lost (YLL)

YLLs following a premature death due to COVID-19 were calculated as the sum of the number of deaths (N) multiplied by life expectancy (L) for the age at death. We obtained the number of deaths and age at death (in ten-year age groups, up to 80 years plus) from the epidemiological model for each scenario. These were multiplied by a reference life expectancy for each exemplar country groupings from WHO lifetables for each 10-year age band. For Group A, we use the Japan life table given 'older' high-income countries in WPR have higher life expectancies than the global high-income country lifetable. For Groups B and C, we use the global lower-middle income lifetable, given 'younger' countries in WPR have a lower life expectancy than global upper-middle income and the WPR life table. In the base case, we discounted future YLLs at 3% annually according to the following formula:

$$YLL = \frac{N(1 - e^{-0.03L})}{0.03}. \tag{17}$$

Years lived with disability (YLD)

The YLD component was calculated for the acute phase of the disease and post-acute consequences following severe disease. We do not include long-COVID due to a lack of available data to specify this condition. We classified cases into four following mutually exclusive categories: asymptomatic, symptomatic non-hospitalized, hospitalized without ICU stay, and hospitalized with ICU stay. We specified an illness severity pathway for each category consisting of four health states (mild/moderate, severe, critical, and post-acute). The post-acute phase refers to the recovery period following hospitalisation, and has been expressed in other cost-effectiveness models [41]. Based on the illness severity pathway for these categories (Table D8), we calculated YLDs by summing up the product of time spent in each health state, the disability weight for that state, and the number of incident cases. YLDs have been calculated using the following formula:

$$YLD = \sum_i I_i \times L_i \times DW_i, \tag{18}$$

where i is an index for health state, I_i is the number of incident cases for each health state, L_i is the duration of disability in years, and DW_i is the disability weight. The duration of illness for each state was based on the average length of hospital and ICU stay from the literature (Table D8).

Table D8: Health states, duration of illness, and disability weights for calculating years lived with disability.

COVID-19 patient category	Health state	Days in state Base case (range)	Disability weight Base case (range)	Notes and sources
A. asymptomatic cases	Not applicable	Not applicable	Not applicable	Zero disability for asymptomatic cases.
B. symptomatic non-hospitalized	Mild or moderate	7·0 (2·0–9·5)	0·051 (0·032–0·074)	GBD 2019 disability weight for moderate lower respiratory infections [42]. Days in mild/moderate state from literature [41, 43]. Assume no post-acute phase.
C. hospitalized without ICU stay	Mild or moderate	7·0 (2·0–9·5)	0·051 (0·032–0·074)	Disability weights from GBD 2019 [42] for moderate lower respiratory infections severe lower respiratory infections and post-acute consequences (fatigue, emotional lability, insomnia) for infectious disease. Using 7 days from symptom onset to hospital, same as category B patients and a Belgian study [44]. Using same number of hospital days (5 days) used in category D patients [45]. Assume 1-week post-acute phase +/- 50% [41].
	Severe	5·0 (3·0–9·0)	0·133 (0·088–0·190)	
	Post-acute	7·0 (3·5–10·5)	0·219 (0·148–0·308)	
D. hospitalized with ICU stay	Mild or moderate	7·0 (2·0–9·5)	0·051 (0·032–0·074)	Disability weights from Nomura 2021 (critical) [46] and GBD 2019 (others) [42]. Symptom onset to ICU discharge of 18 days [43]. Out of this number, assume 7 days from symptom to hospital [44] 5 days in hospital [45] and 7 days in ICU [45]. Assume 2 weeks post-acute phase +/- 50% [41].
	Severe	5·0 (3·0–9·0)	0·133 (0·088–0·190)	
	Critical	7·0 (4·0–11·0)	0·675 (0·506–0·822)	
	Post-acute	7·0 (3·5–10·5)	0·219 (0·148–0·308)	

D.5 Cost-effectiveness analysis

D.5.1 Cost-effectiveness thresholds

Cost-effectiveness thresholds (CETs) for the country groups were based on estimates by Woods et al [47]. This study estimated these thresholds for several countries using the opportunity cost of additional costs incurred from interventions, the relationship between country gross domestic product (GDP) per capita, and the value of a statistical life. These CETs were calculated in 2013 US dollar values. The study reports the lower and upper bounds (limits) of the CETs (per quality-adjusted life-year gained) as percentage of GDP per capita in 2013. Ochalek et al. [48] estimated CETs per DALYs averted, but only three countries in our study were included (Cambodia, Mongolia, and Philippines).

To obtain CETs for use in this study, we multiplied the CETs as percentage of GDP per capita with GDP per capita in 2020 from the World Bank [49], rounded to the nearest hundred. The estimates are presented in Table D9. Thereafter, we calculated the average threshold (separately for the lower and upper bounds) for each group. The CET was between \$19,000 and \$30,000 for Group A, between \$200 and \$1600 for Group B, and between \$100 and \$1000 for Group C countries. The thresholds for each country within the groups are presented in Table D10.

Table D9: Estimated willingness to pay thresholds.

Age demographics / prior primary vaccine coverage	Representative countries / Areas	Threshold range (2020 USD)
Group A: Older population (all countries, high vaccination coverage)	Japan, Australia, Republic of Korea, Hong Kong, Brunei Darussalam, New Zealand, and Singapore	\$19,000–\$30,000
Group B: Younger population (all countries, varying vaccine coverage)	Fiji, Samoa, Tonga, Mongolia, Cambodia, Philippines, Lao, Vanuatu, Kiribati, Micronesia, PNG, and Solomon Islands	\$200–\$1,600
Group C: Younger population (low vaccine coverage)	PNG and Solomon Islands	\$100–\$1,000

Table D10: Willingness To Pay Thresholds for the Western Pacific Region.

Country	GDP per capita in 2020 (A)[1]	% of GDP per capita Woods lower bound (B1)[2]	% of GDP per capita Woods upper bound (B2)[2]	Cost-effectiveness threshold, Woods lower bound (A×B1)[3]	Cost-effectiveness threshold, Woods upper bound (A×B2)[3]
Japan	$40,193	48·30%	48·50%	$19,428	$19,512
Australia	$51,693	48·10%	61·20%	$24,855	$31,651
Korea, Rep.	$31,631	45·00%	50·50%	$14,228	$15,968
Hong Kong	$46,324	45·30%	75·00%	$20,999	$34,741
Fiji	$5,058	10·50%	47·70%	$530	$2,410
Samoa	$4,068	6·60%	47·00%	$267	$1,911
Tonga	$4,625	7·50%	51·40%	$348	$2,376
Mongolia	$4,061	12·40%	47·80%	$505	$1,939
Cambodia	$1,544	4·30%	51·10%	$67	$789
Philippines	$3,299	8·90%	49·50%	$294	$1,633
Lao PDR	$2,630	6·20%	46·50%	$162	$1,223
Vanuatu	$2,870	4·70%	57·00%	$135	$1,637
Papua New Guinea	$2,757	2·70%	39·30%	$76	$1,084
Solomon Islands	$2,251	2·50%	44·60%	$57	$1,005
Kiribati	$1,654	2·50%	49·40%	$41	$817
Micronesia, Fed. Sts.	$3,565	5·40%	55·00%	$193	$1,960
China	$10,435	16·40%	64·80%	$1,711	$6,763
Guam	$34,624	-	-	-	-
Brunei Darussalam	$27,443	35·90%	87·80%	$9,854	$24,102
New Zealand	$41,441	47·80%	50·30%	$19,821	$20,847
Singapore	$59,798	39·20%	108·30%	$23,452	$64,767

[1] Sourced from World Bank.
[2] CETs were estimated based empirical estimates collected using marginal costs invested and marginal health outcomes across different NHS jurisdictions (k) assumed VSL = value of a life year = income elasticity for QALY, If similar elasticity for v and k exists than estimates were created based on differing GDP income elasticities.
[3] Estimate based on GDP per capita in 2020.

D.5.2 Cost-effectiveness results and interpretation

We present the results as incremental cost-effectiveness ratios (ICERs) for the modelled vaccination options compared to a counterfactual of no further vaccination. These ICERs are presented on a cost-effectiveness plane, which shows the DALYs averted and additional costs for the vaccination scenarios (described above) compared to the cost-effectiveness thresholds for the three scenarios. Results that fall below these thresholds indicate a vaccination strategy that is likely to be cost-effective. We have expressed these costs and outcomes per 100,000 people.

We have also performed one-way sensitivity analyses to determine the impact of various cost (vaccine prices and delivery costs) and epidemiological variables on the ICER. We have presented the results of these analyses on tornado diagrams, which indicates the change in ICER when varying parameters (see Figures E5, E6, E9), thus accounting for parameter uncertainty. For Groups B and C countries, we also explore a vaccine price of $0 per dose in a scenario analysis, to explore the cost-effectiveness from a government perspective of having donated vaccines available to some middle-income countries. Given the uncertainty of home-based care cost, we explore the cost-effectiveness if there is no home-based care cost in the scenario analysis.

Additionally, as we are already varying several key epidemiological and demographic parameters (for example, R_0 through high/low transmission scenarios and younger vs older populations), we can compare these parameters' influence on CE results to the costing parameters. This process has demonstrated apparent differences in CEA by allocation strategy across that broad variation, so it is unlikely that the conclusions of a more detailed uncertainty analysis would vary substantially.

D.5.3 Limitations

There are several limitations of this analysis that warrant mentioning.

1. We do not include testing costs, as previously explained. Testing costs can represent a substantial proportion of total costs related to COVID-19 in some countries, however these remain highly uncertain particularly across modelled future scenarios. It is unclear what impact the exclusion of testing costs would have on findings.
2. While indirect costs due to COVID-19, such as productivity losses, also make up a large proportion of total costs related to COVID-19, these costs have not been included in the current analysis. Accounting for indirect costs would make additional boosting vaccination programs appear more cost-effective than our findings indicate. In future work, a societal perspective may be considered.
3. We have not accounted for vaccine-related side effects, including both the costs and health impacts. These are unlikely to impact on cost-effectiveness findings.
4. We are currently not accounting for the costs or health impacts of long-COVID, due to data limitations.

E SUPPLEMENTARY RESULTS

In this section, we have extended results with further details on scenarios from the main paper and additional scenarios. All scenarios and their associated figures and tables are noted in Table E1.

Table E1: Scenario summary table.

Population type	Transmission potential	Vaccine coverage	Immune escape	Boosting strategy	Figures/Tables
older	high TP	high	1·5 yrs	at 2 yrs, pediatric/high-risk/random boosting	Figs. 2(a), 3(a), 3(c), E1(a), E1(c), E5(a), Tabs. 2(a), E2
younger	high TP	high	1·5 yrs	at 2 yrs, pediatric/high-risk/random boosting	Figs. 2(b), 3(b), 3(d), E1(b), E1(d),Tab. 2(b)
older	high TP	high	2·5 yrs	at 2 yrs, pediatric/high-risk/random boosting	Figs. 2(c), 3(a), 3(c), E2(a), E2(c), Tab. 2(c)
younger	high TP	high	2·5 yrs	at 2 yrs, pediatric/high-risk/random boosting	Figs. 2(d), 3(b), 3(d), E2(b), E2(d), E6, Tab. 2(d)
younger	high TP	high	1·5/2·5 yrs	high-risk boosting at 2 yrs, different costings	Fig. E7(a)
younger	low TP	high	1·5/2·5 yrs	high-risk boosting at 2 yrs, different costings	Fig. E7(b)
older	low TP	high	1·5 yrs	at 2 yrs, pediatric/high-risk/random boosting	Figs. E3(a), E4, E5(b), Tab. E3(a)
older	low TP	high	2·5 yrs	at 2 yrs, pediatric/high-risk/random boosting	Figs. E3(b), E4, Tab. E3(b)
older	high TP	high	1·5 yrs	at 1·75/2/2·25/2·5 yrs or half-yearly	Figs. 4(a), E8(a), Tab. E4(a)
younger	high TP	high	1·5 yrs	at 1·75/2/2·25/2·5 yrs or half-yearly	Figs. 4(b), E8(b), E9(a), Tab. E4(b)
older	high TP	high	2·5 yrs	at 1·75/2/2·25/2·5 yrs or half-yearly	Figs. 4(c), E8(c), Tab. E4(c)
younger	high TP	high	2·5 yrs	at 1·75/2/2·25/2·5 yrs or half-yearly	Figs. 4(d), E8(d), E9(b), Tab. E4(d)
older	high TP	high	2 yrs	at 2 yrs, 65+/55+/45+/35+/25+/16+/5+ yrs boosting	Figs. 5(a), 5(c), Tab. E5(a)
younger	high TP	high	2 yrs	at 2 yrs, 65+/55+/45+/35+/25+/16+/5+ yrs boosting	Figs. 5(b), 5(d), Tab. E5(b)
older	low TP	high	2 yrs	at 2 yrs, 65+/55+/45+/35+/25+/16+/5+ yrs boosting	Figs. E10(a), E11(a) Tab. E5(c)
younger	low TP	high	2 yrs	at 2 yrs, 65+/55+/45+/35+/25+/16+/5+ yrs boosting	Figs. E10(b), E11(b), Tab. E5(d)
younger	high TP	low	2 yrs	at 2 yrs, pediatric vaccination/general vaccination/high-risk boosting	Figs. 6(a), 6(c), 6(d), E14, Tab. E6(a)
younger	high TP	medium	2 yrs	at 2 yrs, pediatric vaccination/general vaccination/high-risk boosting	Figs. 6(b), 6(c), 6(d), Tab. E6(b)
younger	low TP	low	2 yrs	at 2 yrs, pediatric vaccination/general vaccination/high-risk boosting	Figs. E12(a), E13(a), E14, Tab. E6(c)
younger	low TP	medium	2 yrs	at 2 yrs, pediatric vaccination/general vaccination/high-risk boosting	Figs. E12(b), E13(b), Tab. E6(d)
younger	high TP	low	2 yrs	at 2 yrs, high-risk monovalent/bivalent boosting	Fig. E15(a), Tab. E7(a)
younger	high TP	medium	2 yrs	at 2 yrs, high-risk monovalent/bivalent boosting	Fig. E15(b), Tab. E7(b)
younger	low TP	low	2 yrs	at 2 yrs, high-risk monovalent/bivalent boosting	Fig. E16(a), Tab. E7(c)
younger	low TP	medium	2 yrs	at 2 yrs, high-risk monovalent/bivalent boosting	Fig. E16(b), Tab. E7(d)

E.1 High vaccination coverage scenarios: comparing target use groups - extended results

This set of scenarios (Section 3.1.1 in the main paper) considered allocating additional boosting to different target use groups in either pediatric, high-risk, or random boosting strategies. Figures E1 and E2 show the probability sensitivity analysis for selected scenarios from the main text, showing how high-risk boosting is largely cost-effective, while pediatric boosting is largely not cost-effective when considering economic uncertainty.

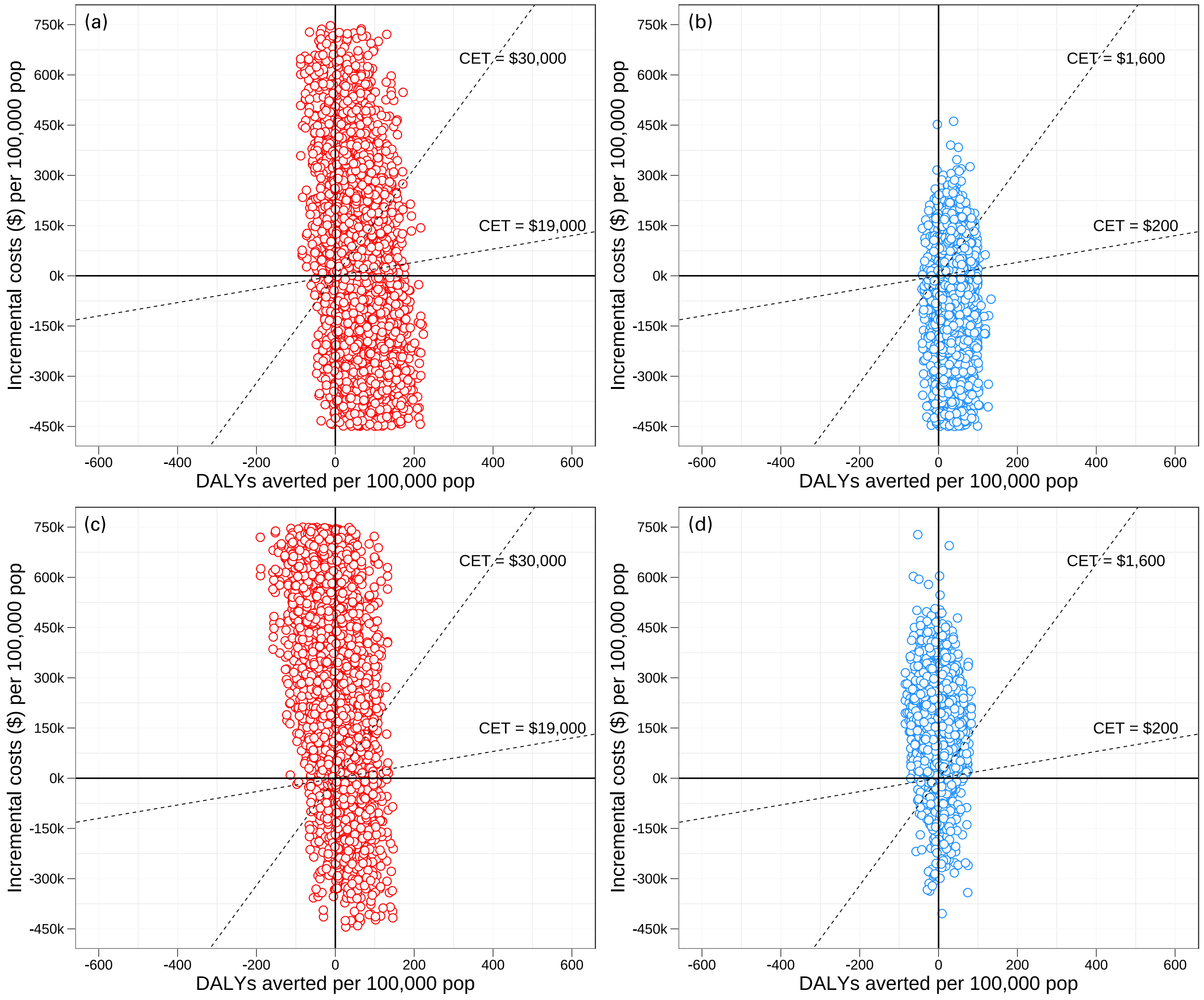

Figure E1: Probability sensitivity analysis of cost-effectiveness in the high transmission, high vaccination coverage setting, for older and younger demographics. Scenarios are run with boosting strategies at 2 years, with immune escape at 1·5 years, for a population of 100,000 individuals. The dotted lines represent cost effective thresholds. **(a)** high risk boosting in the older population; **(b)** high risk boosting in the younger population; **(c)** pediatric boosting in the older population; **(d)** pediatric boosting in the younger population.

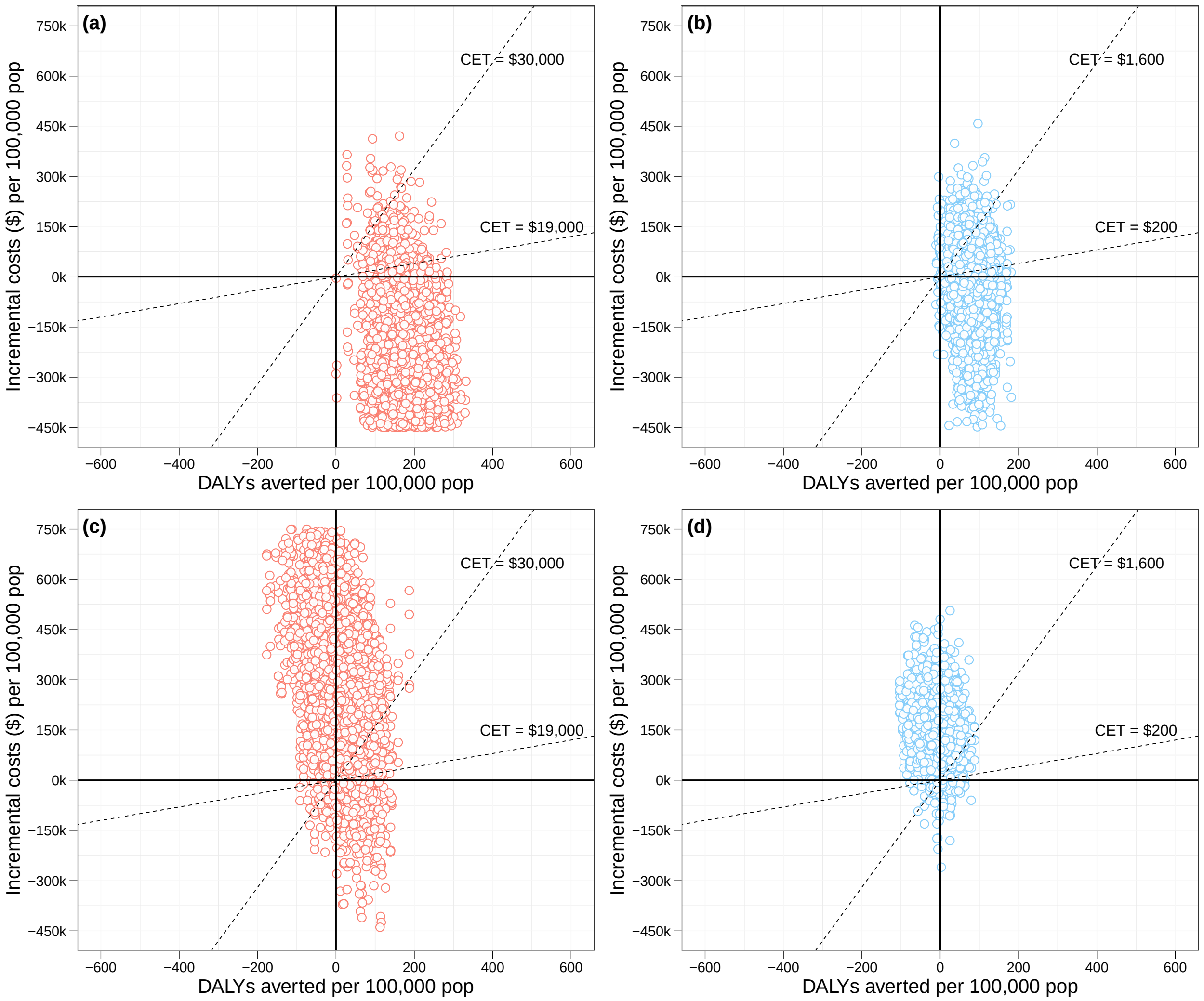

Figure E2: Probability sensitivity analysis of cost-effectiveness in the high transmission, high vaccination coverage setting, for older and younger demographics. Scenarios are run with boosting strategies at 2 years, with immune escape at 2·5 years, for a population of 100,000 individuals. The dotted lines represent cost effective thresholds. **(a)** high risk boosting in the older population; **(b)** high risk boosting in the younger population; **(c)** pediatric boosting in the older population; **(d)** pediatric boosting in the younger population.

Table E2 shows the percentage of infections by vaccination status of the infected, during the time period of 1.5–3 years. Note that individuals may be infected multiple times during the time-frame, and aside from the youngest age group (0-4 years), all other age groups had over 100% infections. These numbers correspond to asymptomatic and symptomatic infections in 80%–153% of the vaccinated population in one year, or an average of 0.8–1.5 infections per person (meanwhile, the number of symptomatic infections corresponds to 23%–101% of the vaccinated population for 1.5 years, or 0.15–0.67 infections per person per year). By proportion, we find the unvaccinated groups had more infections than the vaccinated groups.

This is higher than the estimate available for Austria, with estimated infections amounting to 35% of the Austrian population between May 2023 and May 2024 [50]. Adjusting the transmission parameter is one major lever in changing the number of simulated infections to adapt the model to a specific population [51] – though note that calibration based on reported cases alone are not sufficient, due to known low case reporting, especially for infections with fewer symptoms [52].

Table E2: Median infections by vaccination status (with 0.025 and 0.975 quantiles) between 1.5–3 years for older demographics, by age group, in the high-transmission scenario with early immune escape (at 1·5 years). The different boosting strategies run at 2 years. Note that a total of 88,002 individuals have some level of vaccination (includes both individuals who eventually receive a booster during 1.5–3 years and those who do not) and 11,998 individuals are unvaccinated. The number of median (and quantile) infections (including asymptomatic infection) are presented as a percentage of vaccinated or unvaccinated individuals, noting that individuals may be infected multiple times during the time-frame.

	Boosting strategy	Age group	Vaccinated individuals	Unvaccinated individuals	Median infections (vaccinated)	Median infections (unvaccinated)
(a)	no further boosting	0-4	0	5738	NA	96·7% (93·5%, 100·1%)
		5-11	7319	631	154·4% (150·9%, 158·7%)	197·5% (189·7%, 206·5%)
		12-15	4187	361	179·7%, (175·7%, 184·3%)	239·1%, (228·0%, 250·2%)
		16-19	4117	355	173·6% (169·9%, 178·4%)	228·7% (217·7%, 240·6%)
		20-24	5851	504	207·0% (203·0%, 211·8%)	286·3% (276·6%, 296·2%)
		25-29	6273	540	224·1% (220·0%, 228·7%)	317·2% (307·6%, 327·4%)
		30-34	6803	587	226·8% (223·0%, 231·4%)	322·0% (312·3%, 332·0%)
		35-39	6703	578	229·0% (225·2%, 233·6%)	326·5% (316·3%, 336·3%)
		40-44	6277	541	227·8% (223·8%, 232·7%)	323·7% (313·3%, 333·3%)
		45-49	6742	581	211·5% (207·7%, 216·0%)	294·5% (285·9%, 304·1%)
		50-54	6573	566	201·2% (197·2%, 205·8%)	276·1% (266·4%, 285·0%)
		55-59	6400	551	184·4% (180·6%, 188·8%)	246·8% (238·1%, 256·3%)
		60-64	5977	134	169·5% (165·5%, 173·9%)	220·1% (204·5%, 238·1%)
		65-69	5056	113	164·2% (160·4%, 168·8%)	210·6% (192·9%, 228·3%)
		70-74	3851	86	148·9% (144·8%, 153·0%)	186·0% (165·1%, 205·8%)
		75-79	2542	57	177·2% (172·9%, 182·5%)	233·3% (205·3%, 257·9%)
		80+	3331	75	154·1% (150·1%, 158·5%)	194·7% (172·0%, 217·3%)
(b)	pediatric boosting (ages 5–15)	0-4	0	5738	NA	95·5% (92·6%, 98·7%)
		5-11	7319	631	122·3% (118·6%, 127·4%)	190·0% (181·5%, 198·9%)
		12-15	4187	361	141·1% (136·6%, 147·1%)	231·6% (220·2%, 242·9%)
		16-19	4117	355	171·9% (167·9%, 176·7%)	225·9% (215·5%, 236·9%)
		20-24	5851	504	206·5% (202·5%, 211·6%)	285·7% (275·2%, 295·6%)
		25-29	6273	540	223·4% (219·3%, 228·0%)	316·1% (306·3%, 326·3%)
		30-34	6803	587	226·0% (222·4%, 231·1%)	320·6% (310·7%, 330·5%)
		35-39	6703	578	228·4% (224·7%, 233·1%)	324·6% (314·7%, 334·8%)
		40-44	6277	541	227·0% (222·8%, 231·8%)	322·4% (312·2%, 332·2%)
		45-49	6742	581	211·1% (207·3%, 215·6%)	293·6% (284·2%, 303·3%)
		50-54	6573	566	200·9% (197·3%, 205·7%)	275·6% (266·4%, 285·9%)
		55-59	6400	551	184·2% (180·6%, 189·1%)	246·8% (237·7%, 256·1%)
		60-64	5977	134	169·2% (165·1%, 173·4%)	220·9% (204·5%, 238·1%)
		65-69	5056	113	164·0% (160·2%, 168·4%)	209·7% (192·0%, 228·3%)
		70-74	3851	86	148·6% (144·8%, 153·1%)	186·0% (166·3%, 204·7%)
		75-79	2542	57	177·3% (172·6%, 182·2%)	233·3% (205·3%, 259·7%)
		80+	3331	75	154·1% (149·8%, 158·3%)	194·7% (172·0%, 214·7%)
(c)	high-risk boosting (65+)	0-4	0	5738	NA	95·2% (92·2%, 98·3%)
		5-11	7319	631	153·8% (150·1%, 157·9%)	196·4% (188·3%, 204·8%)
		12-15	4187	361	179·3% (175·0%, 184·0%)	238·4% (227·4%, 249·6%)
		16-19	4117	355	173·0% (168·9%, 177·9%)	227·9% (216·9%, 238·6%)
		20-24	5851	504	206·6% (202·4%, 211·5%)	285·5% (275·4%, 296·0%)
		25-29	6273	540	223·7% (219·6%, 228·6%)	316·3% (305·7%, 327·0%)
		30-34	6803	587	226·3% (222·5%, 231·3%)	321·1% (311·1%, 331·7%)
		35-39	6703	578	228·6% (224·7%, 233·6%)	324·7% (314·2%, 335·3%)
		40-44	6277	541	226·9% (222·9%, 231·7%)	322·0% (312·2%, 332·7%)
		45-49	6742	581	210·9% (207·0%, 215·8%)	292·9% (283·5%, 302·1%)
		50-54	6573	566	200·2% (196·5%, 205·2%)	274·0% (264·3%, 283·8%)
		55-59	6400	551	182·8% (178·8%, 187·4%)	244·1% (235·4%, 253·2%)
		60-64	5977	134	166·8% (163·1%, 171·5%)	217·2% (200·0%, 235·1%)
		65-69	5056	113	137·0% (133·1%, 142·1%)	204·4% (186·7%, 223·0%)
		70-74	3851	86	124·4% (120·6%, 129·4%)	180·2% (158·1%, 201·2%)
		75-79	2542	57	147·7% (143·3%, 154·1%)	228·1% (201·8%, 254·4%)
		80+	3331	75	128·7% (124·5%, 134·2%)	189·3% (168·0%, 210·7%)
(d)	random boosting	0-4	0	5738	NA	94·9% (91·9%, 98·2%)
		5-11	7319	631	149·5% (145·8%, 153·8%)	194·8% (185·4%, 204·1%)
		12-15	4187	361	173·8% (169·5%, 178·9%)	236·6% (226·0%, 248·2%)
		16-19	4117	355	168·5% (164·3%, 173·2%)	226·2% (214·9%, 238·3%)
		20-24	5851	504	200·9% (196·9%, 206·2%)	284·3% (274·6%, 294·4%)
		25-29	6273	540	217·8% (213·4%, 222·9%)	315·2% (305·6%, 324·8%)
		30-34	6803	587	220·5% (216·4%, 225·6%)	320·3% (310·6%, 330·5%)
		35-39	6703	578	222·5% (218·4%, 227·5%)	324·2% (314·2%, 333·7%)
		40-44	6277	541	221·4% (217·1%, 226·6%)	321·6% (311·3%, 332·2%)
		45-49	6742	581	205·2% (201·1%, 210·2%)	292·6% (283·0%, 301·4%)
		50-54	6573	566	195·3% (191·5%, 200·5%)	273·9% (264·1%, 283·4%)
		55-59	6400	551	178·7% (174·7%, 183·6%)	244·3% (235·8%, 254·3%)
		60-64	5977	134	163·6% (159·8%, 168·4%)	218·7% (200·0%, 235·8%)
		65-69	5056	113	158·9% (154·9%, 163·8%)	208·0% (189·4%, 225·7%)
		70-74	3851	86	144·0% (140·2%, 148·7%)	183·7% (165·1%, 203·5%)
		75-79	2542	57	171·7% (167·4%, 176·8%)	229·8% (207·0%, 256·1%)
		80+	3331	75	149·0% (145·1%, 153·6%)	192·0% (172·0%, 213·4%)

In the low transmission setting, Figure E3 shows the epidemic infection curves, Figure E4 shows the cost-effectiveness analysis, while Table E3 shows the median deaths. We find that high-risk boosting is likely to be cost-effective in the low-transmission setting too.

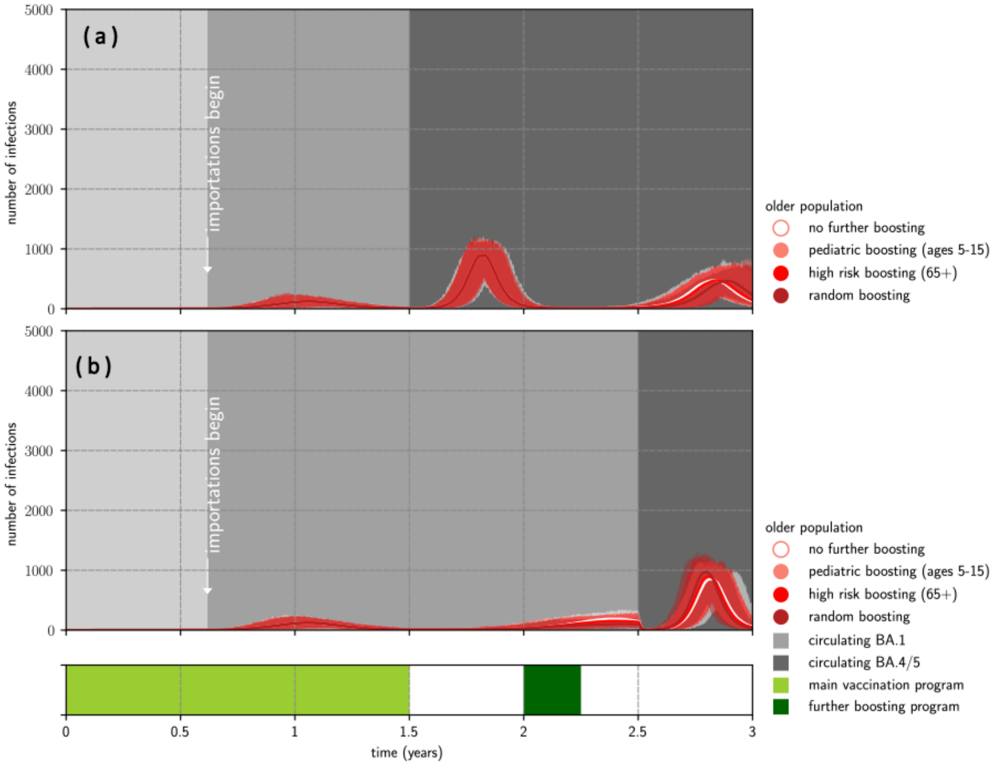

Figure E3: Outbreaks in the low transmission, high vaccination coverage setting, for older demographics. **(a)** epidemic waves given early immune escape (1·5 years); **(b)** epidemic waves given late immune escape (2·5 years). Scenarios are run with no further boosting, pediatric, high-risk and random boosting strategies at 2 years. The solid lines represent the pointwise median infections from 1000 simulations and the shaded regions represent the pointwise maximum and minimum infections. Results are for a population of 100,000 individuals.

Figure E5 shows the one-way sensitivity analysis of a high-risk boosting strategy in the high and low transmission settings, with immune escape at 1·5 years. We find that vaccine delivery cost and unit cost per dose are the most influential costing parameters for older population scenarios.

Figure E6 shows the one-way sensitivity analysis for high-risk boosting, this time in the high-transmission high-vaccination setting with a younger demographic, with immune escape at 2·5 years. Here, we find that home-based care cost, vaccine delivery cost, and vaccine cost per dose are the most influential costing parameters.

Figure E7 compares the cost-effectiveness of high-risk boosting in younger population, in four different scenarios (base, vaccine donated, no home care cost, vaccine donated and no home care cost). When vaccines are donated (i.e. vaccine dose costs are zero), high-risk boosting may be more cost-effective than non-donated scenarios. If home-based care costs are zero, then high-risk boosting is likely to be *not* cost-effective. If both vaccine dose costs and home care costs are zero, high-risk boosting may be cost-effective when immune escape starts 2·5 years.

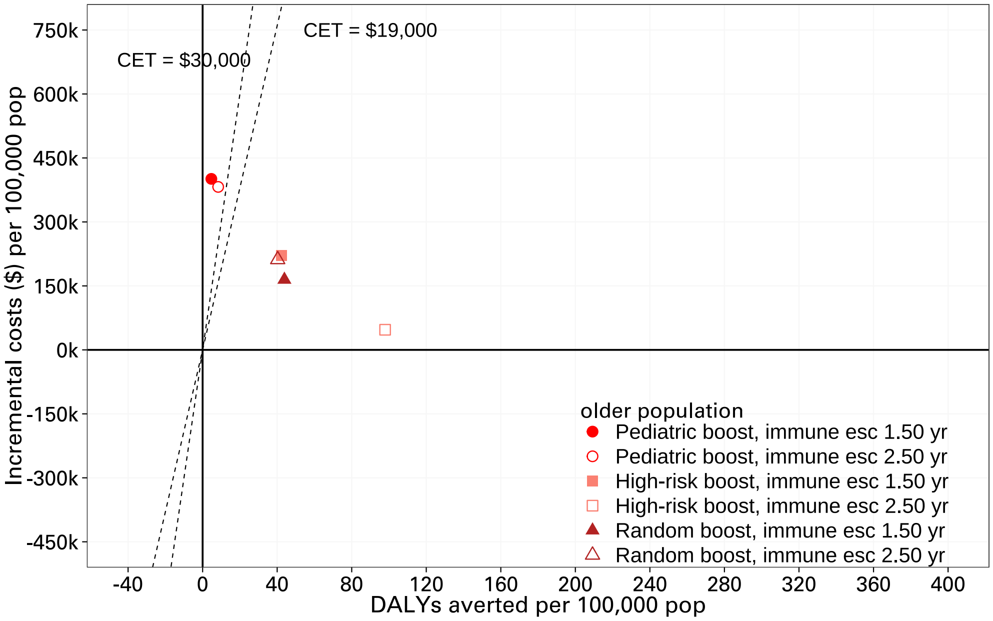

Figure E4: Cost-effectiveness analysis in the low transmission, high vaccination coverage setting, for older demographics. Scenarios are run with no further boosting, pediatric, high-risk and random boosting strategies at 2 years. The dotted lines represent cost effective thresholds. Results are for a population of 100,000 individuals.

Table E3: Median deaths (with 0.025 and 0.975 quantiles) between 1.5–3 years in the low-transmission high vaccination coverage setting, for older demographics, comparing target use groups. Early immune escape occurs at 1·5 years; late immune escape occurs at 2·5 years. The different boosting strategies run at 2 years. Results are for a population of 100,000 individuals.

	**Transmission potential**	**Immune escape**	**Boosting strategy**	**Median deaths**
(a)	low TP	early	no further boosting	39·0 (28·0, 51·0)
	low TP	early	pediatric boosting (ages 5–15)	39·0 (28·0, 51·0)
	low TP	early	high-risk boosting (65+)	36·0 (25·0, 48·0)
	low TP	early	random boosting	36·0 (25·0, 48·0)
(b)	low TP	late	no further boosting	31·0 (20·0, 43·0)
	low TP	late	pediatric boosting (ages 5–15)	30·0 (19·0, 42·02)
	low TP	late	high-risk boosting (65+)	23·0 (14·0, 34·0)
	low TP	late	random boosting	28·0 (18·0, 40·0)

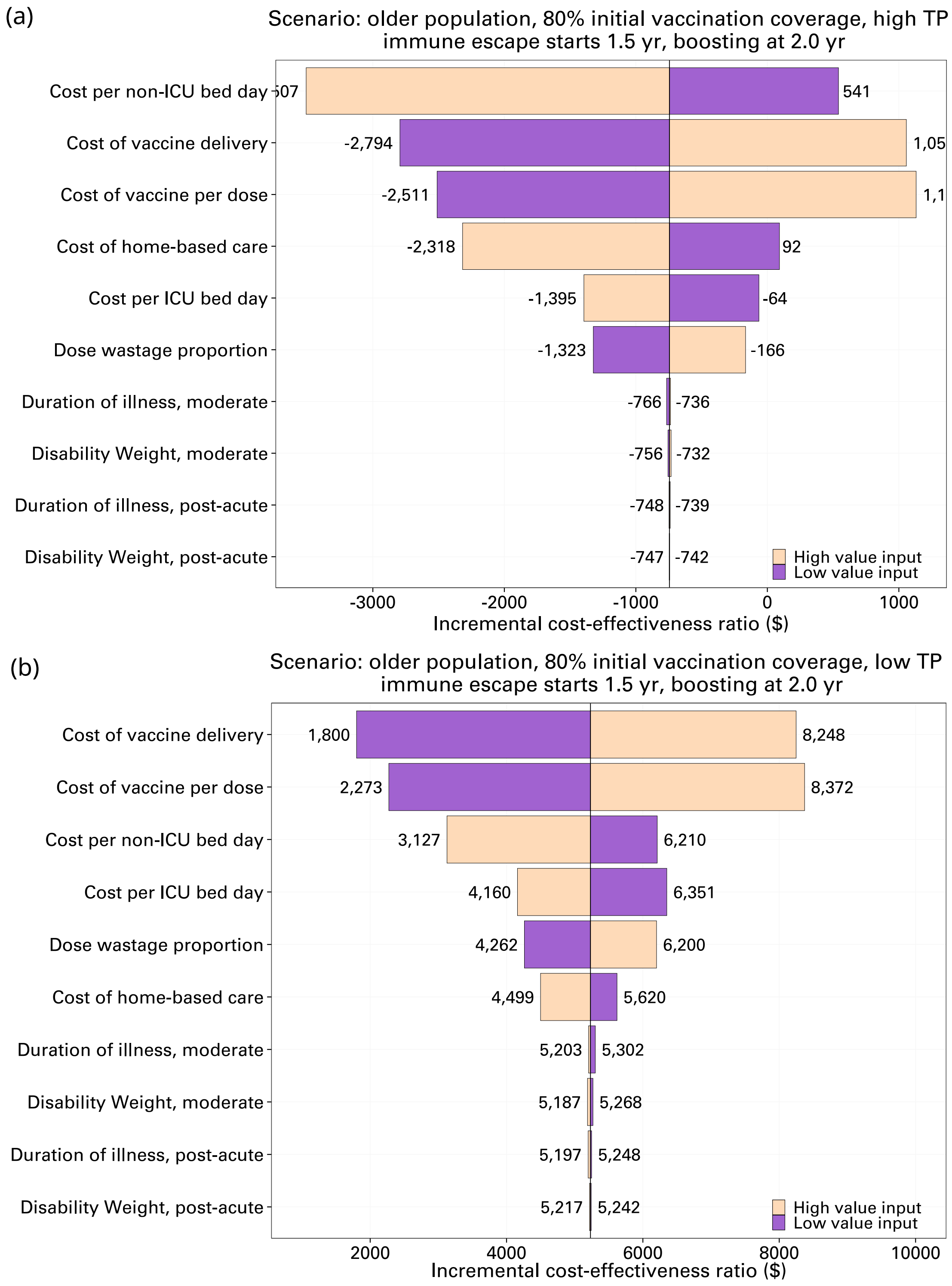

Figure E5: One-way sensitivity analysis for high-risk boosting at 2·0 years in an older population with high vaccination coverage. Here, immune escape occurs at 1·5 years. **(a)** in the high transmission potential scenario; **(b)** in the low transmission potential scenario. See Table D4 for the parameter ranges. Results are for a population of 100,000 individuals.

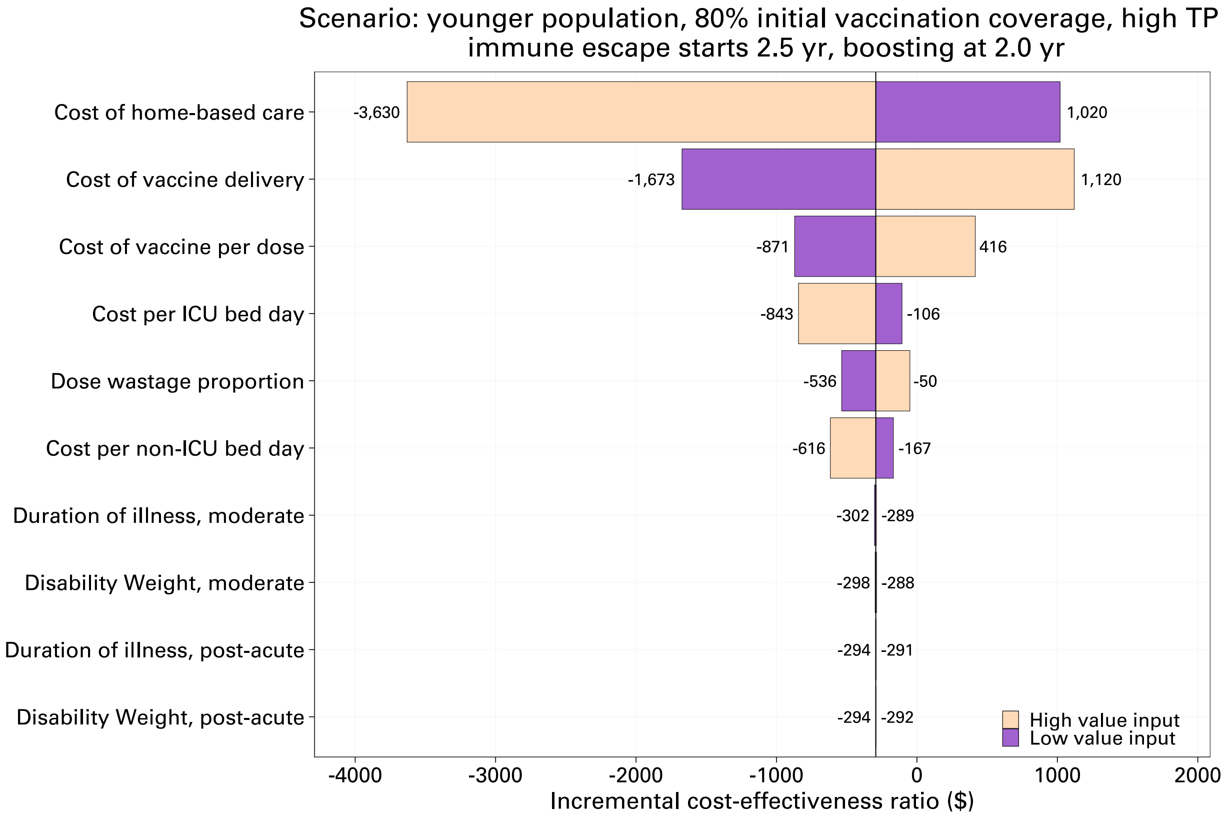

Figure E6: One-way sensitivity analysis for high-risk boosting at 2·0 years in a younger population with high transmission potential and high vaccination coverage. Here, immune escape occurs at 2·5 years. See Table D4 for the parameter ranges. Results are for a population of 100,000 individuals.

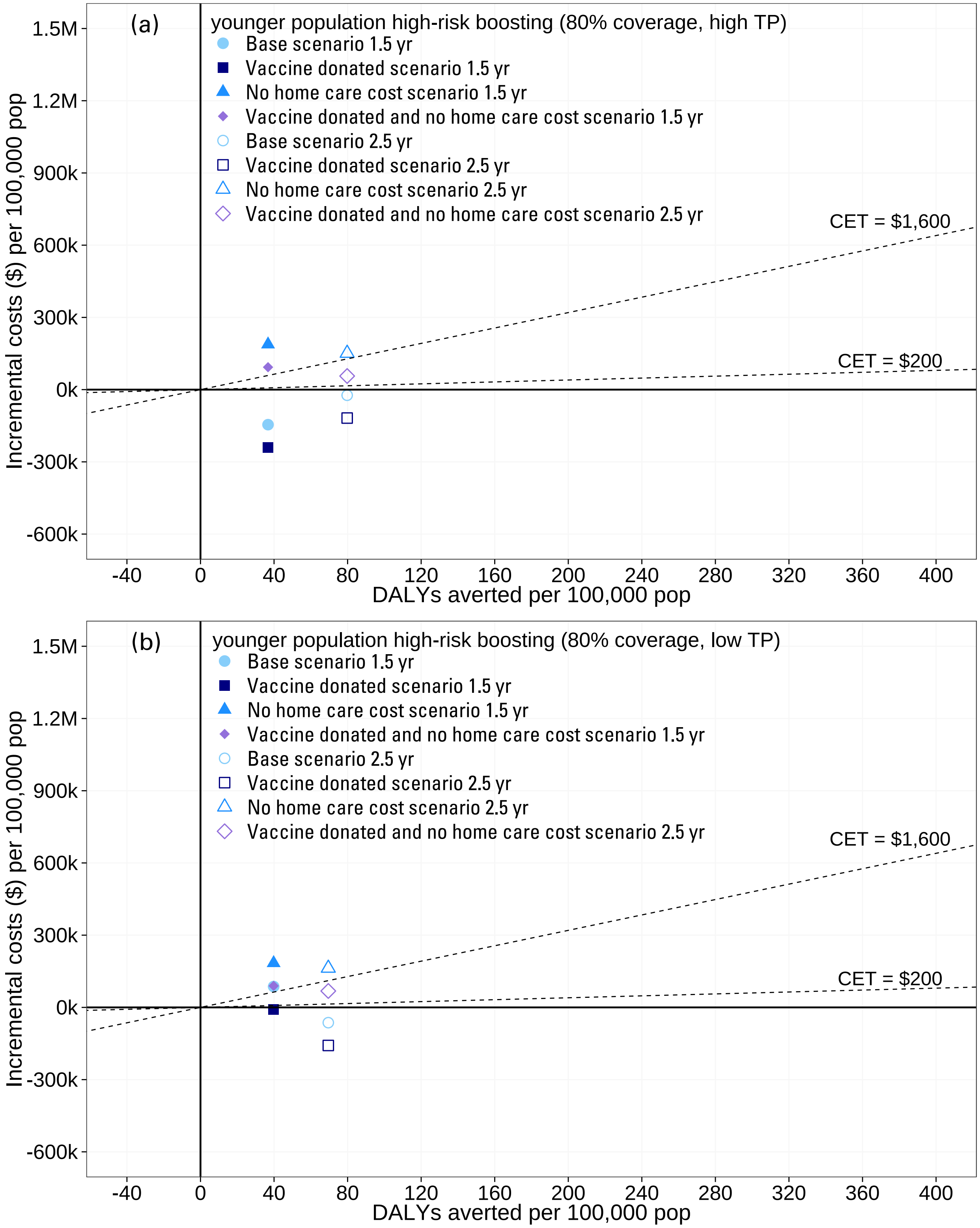

Figure E7: Cost-effectiveness analysis of high-risk boosting in the high vaccination coverage setting, for younger demographics, given different cost settings. **(a)** High transmission setting; **(b)** low transmission setting. Scenarios are run with boosting at 2 years. The dotted lines represent cost effective thresholds. Results are for a population of 100,000 individuals.

E.2 High vaccination coverage scenarios: Boosting frequency - extended results

This set of scenarios (Section 3.1.2 in the main paper) considered timing of boosting, and compared a once-off boosting with a half-yearly boosting schedule. Table E4 shows the median deaths (clinical outcomes), where we see a clear reduction in deaths under a half-yearly boosting schedule as compared to other boosting strategies. Figure E8 shows the cost-effectiveness analysis: high-risk half-yearly boosting is cost-effective in older populations, though it is more expensive than boosting only once. Meanwhile, Figure E9 shows one-way sensitivity analyses for the half-yearly boosting scenarios, showing that home-based care cost is the most influential costing parameter in younger populations.

Table E4: Median deaths (with 0.025 and 0.975 quantiles) between 1.5–3 years in high transmission settings with high vaccination coverage, comparing the boosting timing with half-yearly boosting. Early immune escape occurs at 1·5 years; late immune escape occurs at 2·5 years. Results are for a population of 100,000 individuals.

	Population	Immune escape	Boosting timing	Median deaths
(a)	older	early escape	1·75 years	35·0 (24·0, 47·0)
	older	early escape	2·0 years	34·0 (23·0, 46·0)
	older	early escape	2·25 years	36·0 (25·0, 48·0)
	older	early escape	2·5 years	40·0 (28·0, 52·0)
	older	early escape	half-yearly	32·0 (21·0, 42·0)
(b)	younger	early escape	1·75 years	9·0 (4·0, 15·0)
	younger	early escape	2·0 years	9·0 (4·0, 15·0)
	younger	early escape	2·25 years	9·0 (4·0, 16·0)
	younger	early escape	2·5 years	10·0 (5·0, 17·0)
	younger	early escape	half-yearly	8·0 (3·0, 14·0)
(c)	older	late escape	1·75 years	35·0 (24·0, 47·0)
	older	late escape	2·0 years	34·0 (23·0, 47·0)
	older	late escape	2·25 years	34·0 (23·0, 47·0)
	older	late escape	2·5 years	36·0 (25·0, 50·0)
	older	late escape	half-yearly	26·0 (17·0, 37·0)
(d)	younger	late escape	1·75 years	8·0 (3·0, 15·0)
	younger	late escape	2·0 years	9·0 (4·0, 16·0)
	younger	late escape	2·25 years	8·0 (3·0, 15·0)
	younger	late escape	2·5 years	9·0 (4·0, 15·0)
	younger	late escape	half-yearly	4·0 (1·0, 9·0)

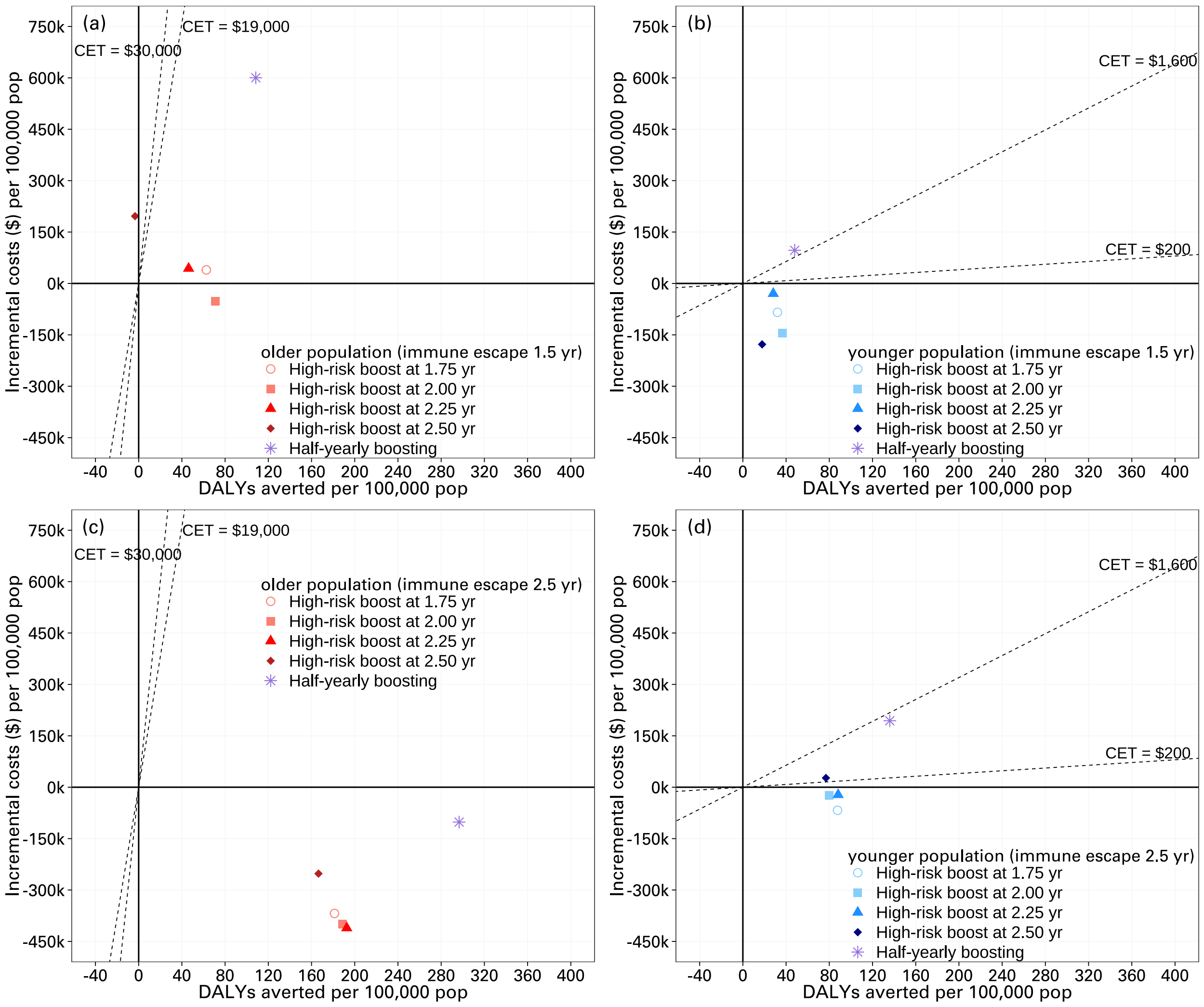

Figure E8: Cost-effectiveness analyses comparing frequent boosting and boosting once at a range of times in the high-transmission high-vaccination coverage settings, for older and younger demographics. (a) older population with early immune escape (1·5 years); **(b)** younger population with early immune escape (1·5 years); **(c)** older population with late immune escape (2·5 years); **(d)** younger population with late immune escape (2·5 years). The high-risk boosting (65+ in the older population, 55+ in the younger population) is rolled out at either 1·75 years, 2·0 years, 2·25 years, 2·5 years, or half-yearly starting from 1·75 years. Results are for a population of 100,000 individuals. High-risk boosting is likely to be cost-effective, though more frequent boosting may not be cost-effective in younger populations.

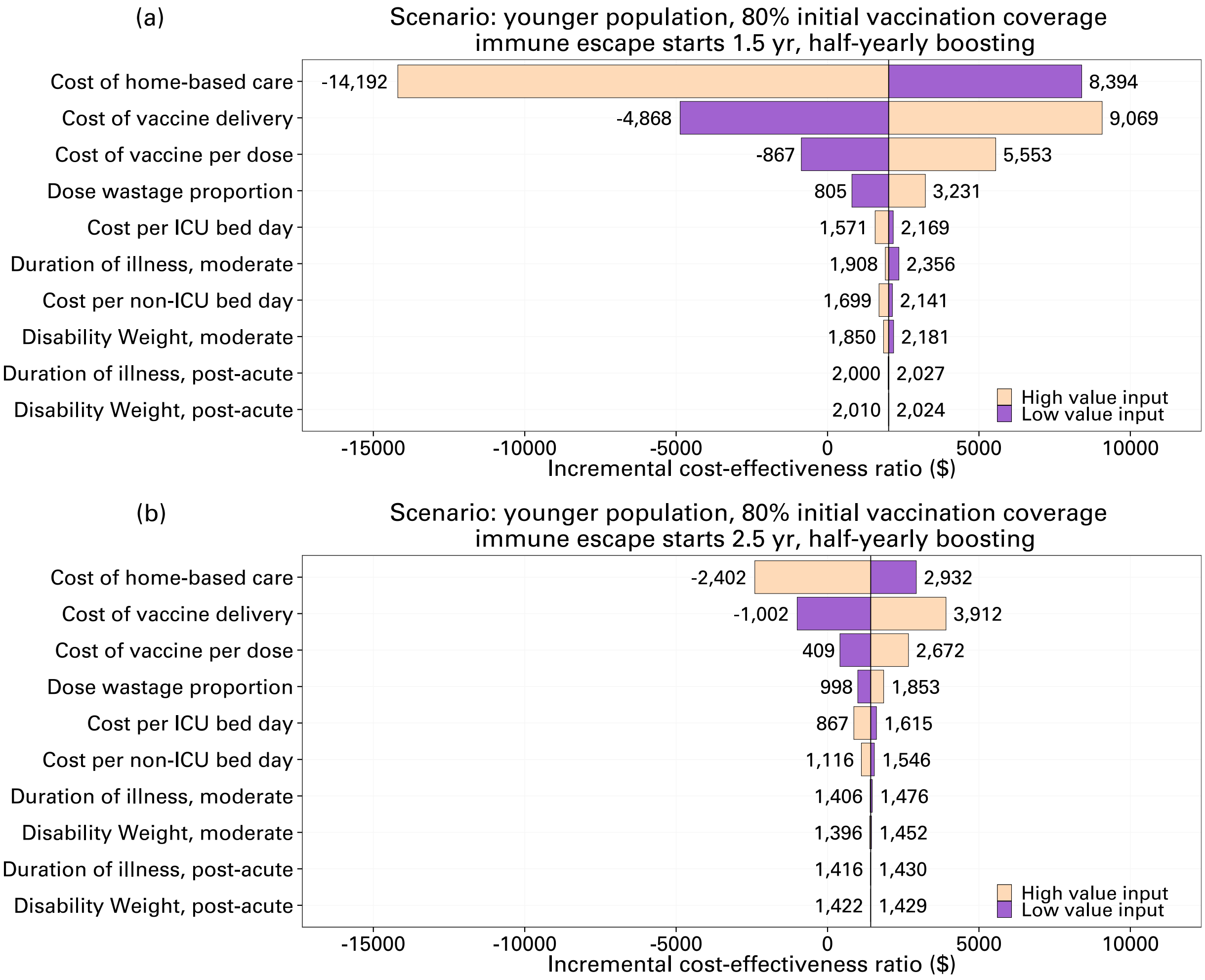

Figure E9: One-way sensitivity analysis for the half-yearly high-risk boosting strategy, in a younger population with high transmission potential and high vaccination coverage. **(a)** With early immune escape at 1·5 years; **(b)** with late immune escape at 2·5 years. See Table D4 for the parameter ranges. Results are for a population of 100,000 individuals.

E.3 High vaccination coverage scenarios: age-cutoff for cost-effective boosting - extended results

This set of scenarios (Section 3.1.3 in the main paper) considered systematically reducing the "high risk boosting" age threshold from 65+ downwards. Figure E10 shows the epidemic infection curves in a low-transmission setting, and Figure E11 shows the cost-effectiveness of different age-thresholds. Overall, the relative results are similar to that of the high-transmission setting, i.e. that boosting 45+ is cost-effective, and there are only minimal gains from decreasing the age threshold down further. Table E5 provides the median deaths in both high transmission and low transmission settings for completeness.

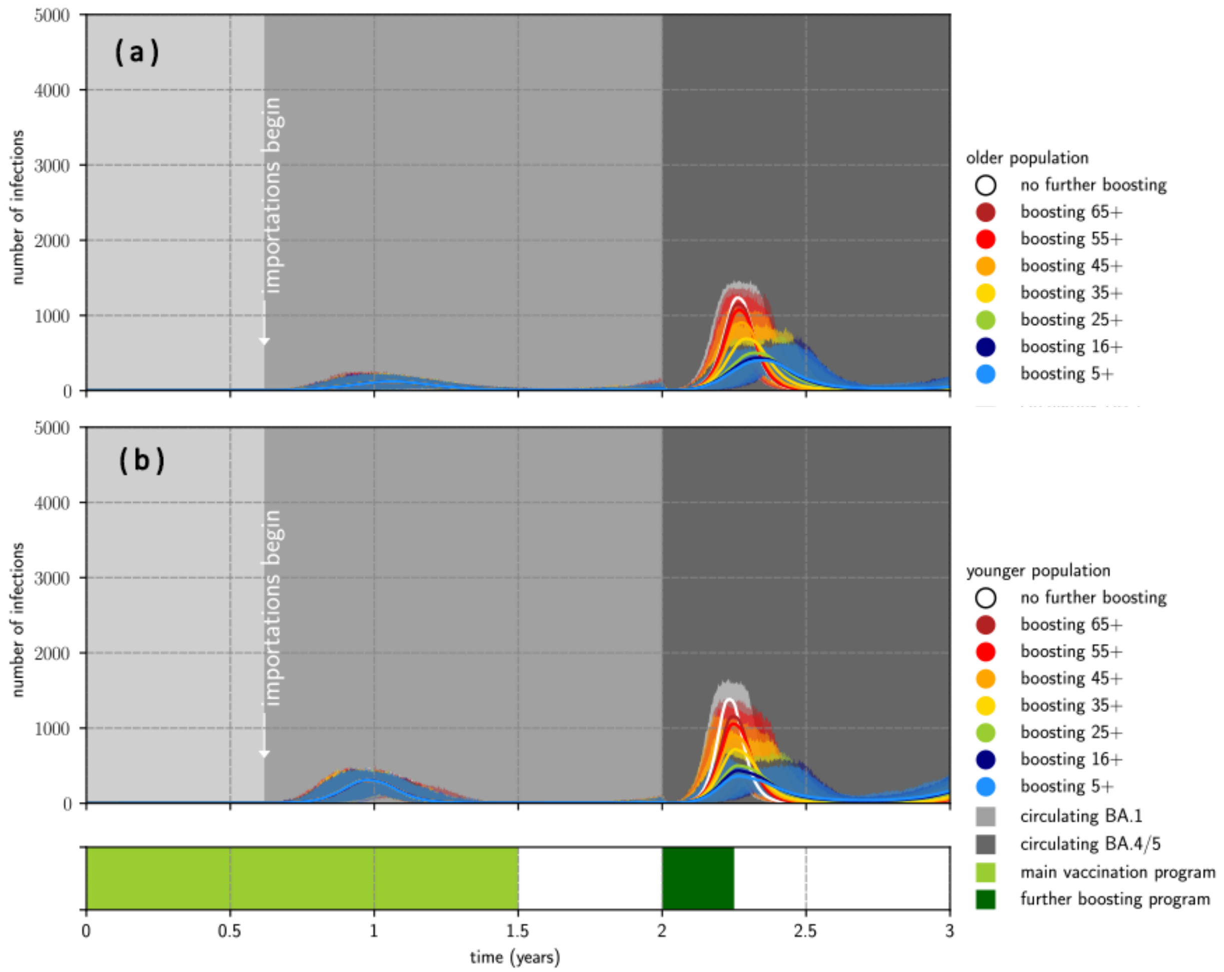

Figure E10: Outbreaks in the low transmission, high vaccination coverage setting, for older and younger demographics, comparing the impact of lowering the age cut-off for high risk boosting. (a) epidemic waves in the older population; **(b)** epidemic waves in the younger population. All scenarios here had an immune escape variant seeded at 2 years, with boosting at 2 years. The solid lines represent the pointwise median infections from 1000 simulations and the shaded regions represent the pointwise maximum and minimum infections. Results are for a population of 100,000 individuals.

Table E5: Median deaths (with 0.025 and 0.975 quantiles) between 1.5–3 years in the high vaccination coverage setting, for older and younger demographics, comparing the impact of lowering the age cut-off for high risk boosting. All scenarios here had an immune escape variant seeded at 2 years, with boosting at 2 years. Results are for a population of 100,000 individuals.

	Population	Transmission potential	Age group	Median deaths
(a)	older	high TP	no further boosting	47·0 (35·0, 60·0)
	older	high TP	boosting 65+	34·0 (24·0, 45·0)
	older	high TP	boosting 55+	31·0 (21·0, 42·0)
	older	high TP	boosting 45+	29·0 (19·0, 41·0)
	older	high TP	boosting 35+	30·0 (20·0, 42·0)
	older	high TP	boosting 25+	28·0 (18·0, 40·0)
	older	high TP	boosting 16+	28·0 (18·0, 38·0)
	older	high TP	boosting 5+	29·0 (19·0, 39·0)
(b)	younger	high TP	no further boosting	15·0 (8·0, 23·0)
	younger	high TP	boosting 65+	12·0 (6·0, 20·0)
	younger	high TP	boosting 55+	11·0 (5·0, 18·0)
	younger	high TP	boosting 45+	10·0 (5·0, 18·0)
	younger	high TP	boosting 35+	10·0 (4·0, 17·02)
	younger	high TP	boosting 25+	10·0 (4·0, 17·0)
	younger	high TP	boosting 16+	10·0 (4·0, 16·0)
	younger	high TP	boosting 5+	10·0 (4·0, 17·0)
(c)	older	low TP	no further boosting	33·0 (22·0, 46·0)
	older	low TP	boosting 65+	21·0 (12·0, 31·0)
	older	low TP	boosting 55+	17·0 (10·0, 26·0)
	older	low TP	boosting 45+	16·0 (8·98, 25·0)
	older	low TP	boosting 35+	15·0 (8·0, 24·0)
	older	low TP	boosting 25+	15·0 (8·0, 23·0)
	older	low TP	boosting 16+	15·0 (8·0, 24·0)
	older	low TP	boosting 5+	15·0 (8·0, 24·0)
(d)	younger	low TP	no further boosting	15·0 (8·0, 23·0)
	younger	low TP	boosting 65+	10·0 (4·0, 17·0)
	younger	low TP	boosting 55+	8·0 (3·0, 15·0)
	younger	low TP	boosting 45+	7·0 (3·0, 13·0)
	younger	low TP	boosting 35+	7·0 (2·0, 13·0)
	younger	low TP	boosting 25+	7·0 (2·0, 13·0)
	younger	low TP	boosting 16+	7·0 (2·0, 13·0)
	younger	low TP	boosting 5+	7·0 (2·0, 13·0)

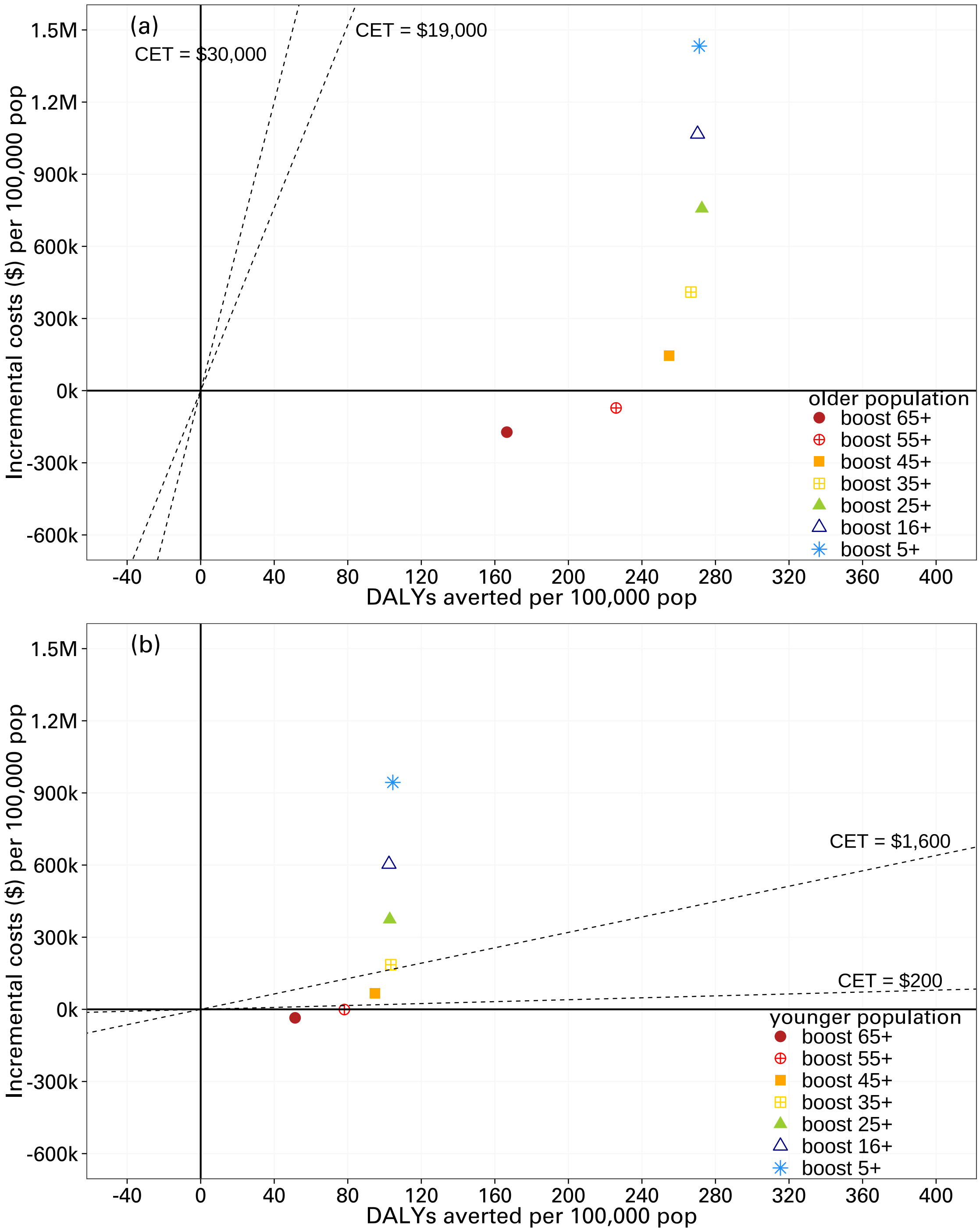

Figure E11: Cost-effectiveness analysis of vaccination in the low transmission, high vaccination coverage setting, for older and younger demographics, comparing the impact of lowering the age cut-off for high risk boosting. (a) older population; **(b)** younger population. All scenarios here had an immune escape variant seeded at 2 years, with boosting at 2 years. Results are for a population of 100,000 individuals. Boosting 65+ and 55+ is likely to be cost-effective or cost-saving.

E.4 Low-medium vaccination coverage: comparing primary and booster strategies - extended results

This set of scenarios (Section 3.2.1 in the main paper) considered the trade-off between new primary vaccination and high risk boosting strategies in younger populations with low or medium vaccination coverage. Figure E12 shows the epidemic infection curves in a low-transmission setting, Figure E13 shows the cost-effectiveness of different strategies for both high and low transmission settings, and Table E6 provides the median deaths in high and low transmission settings. The results show that high-risk boosting may be cost-effective in medium vaccination coverage settings. Finally, Figure E14 shows a cost-effectiveness analysis of high-risk boosting in the low vaccination coverage setting given different cost settings, including the scenario where vaccines are donated, or if there are no home care costs. We find that high-risk boosting would be more cost-effective or even cost-saving if vaccines are donated.

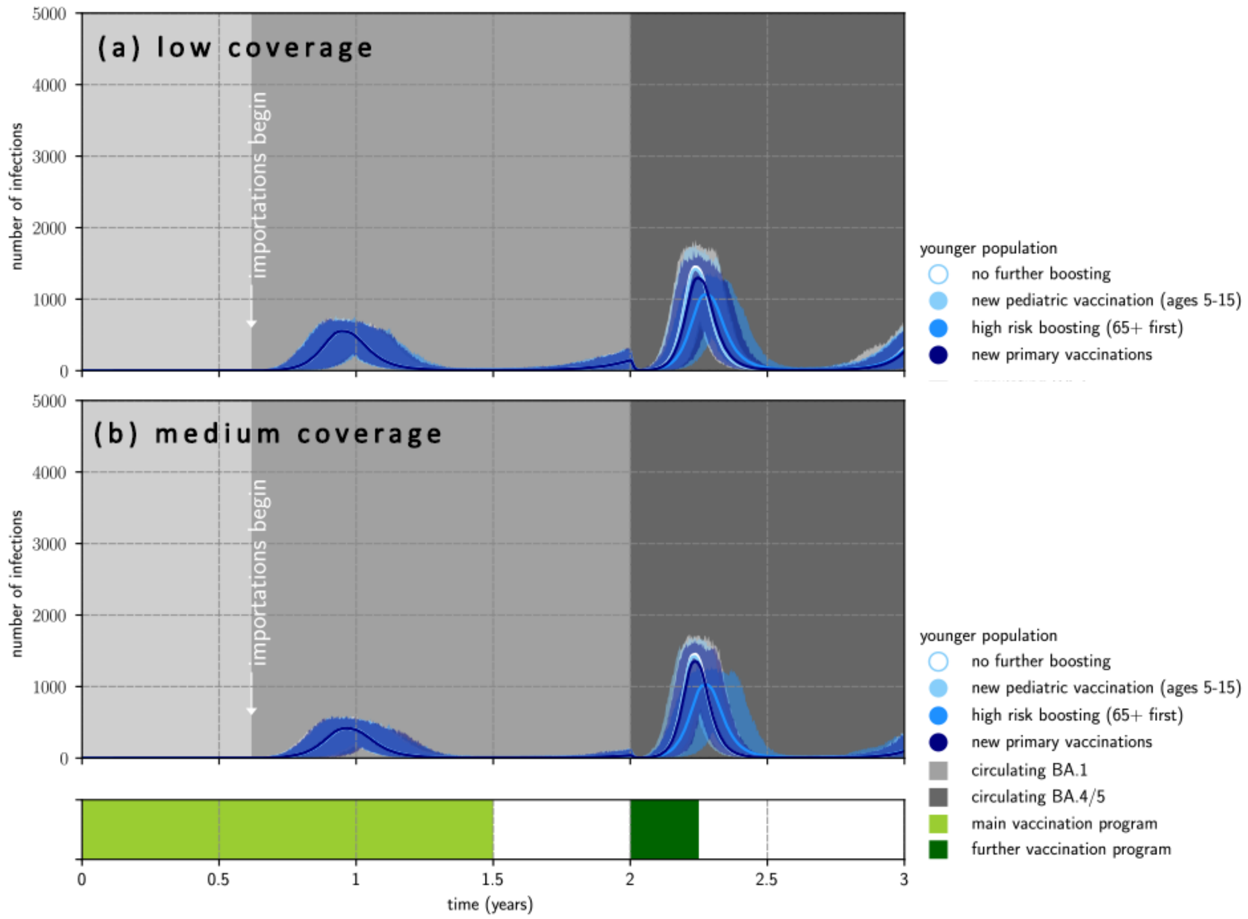

Figure E12: Outbreaks in the low transmission, low- and medium-vaccination coverage settings, for younger demographics. **(a)** epidemic waves in a younger population with low vaccination coverage (initial coverage 20%); **(b)** with medium vaccination coverage (initial coverage 50%). All scenarios had an immune escape variant seeded at 2 years, with additional vaccination or boosting at 2 years. The solid lines represent the pointwise median infections from 1000 simulations and the shaded regions represent the pointwise maximum and minimum infections. Results are for a population of 100,000 individuals.

Table E6: Median deaths (with 0.025 and 0.975 quantiles) between 1.5–3 years in the low-medium vaccination coverage setting, for younger demographics, comparing primary and booster strategies. All scenarios had an immune escape variant seeded at 2 years, with additional vaccination or boosting at 2 years. Results are for a population of 100,000 individuals.

	Transmission potential	Vaccine coverage	Vaccination strategy	Median deaths
(a)	high TP	low	no further boosting	16·0 (9·0, 24·0)
	high TP	low	new pediatric vaccination (ages 5–15)	15·0 (8·0, 24·0)
	high TP	low	high-risk boosting (65+ first)	12·0 (5·0, 19·0)
	high TP	low	new primary vaccinations	14·0 (7·0, 22·0)
(b)	high TP	medium	no further boosting	14·0 (7·0, 21·0)
	high TP	medium	new pediatric vaccination (ages 5–15)	14·0 (7·0, 21·0)
	high TP	medium	high-risk boosting (65+ first)	10·0 (5·0, 16·0)
	high TP	medium	new primary vaccinations	13·0 (7·0, 21·0)
(c)	low TP	low	no further boosting	16·0 (9·0, 24·0)
	low TP	low	new pediatric vaccination (ages 5–15)	16·0 (9·0, 24·0)
	low TP	low	high-risk boosting (65+ first)	11·0 (5·0, 17·0)
	low TP	low	new primary vaccinations	14·0 (8·0, 22·0)
(d)	low TP	medium	no further boosting	16·0 (9·0, 25·0)
	low TP	medium	new pediatric vaccination (ages 5–15)	16·0 (9·0, 24·0)
	low TP	medium	high-risk boosting (65+ first)	8·0 (3·0, 15·0)
	low TP	medium	new primary vaccinations	15·0 (8·0, 24·0)

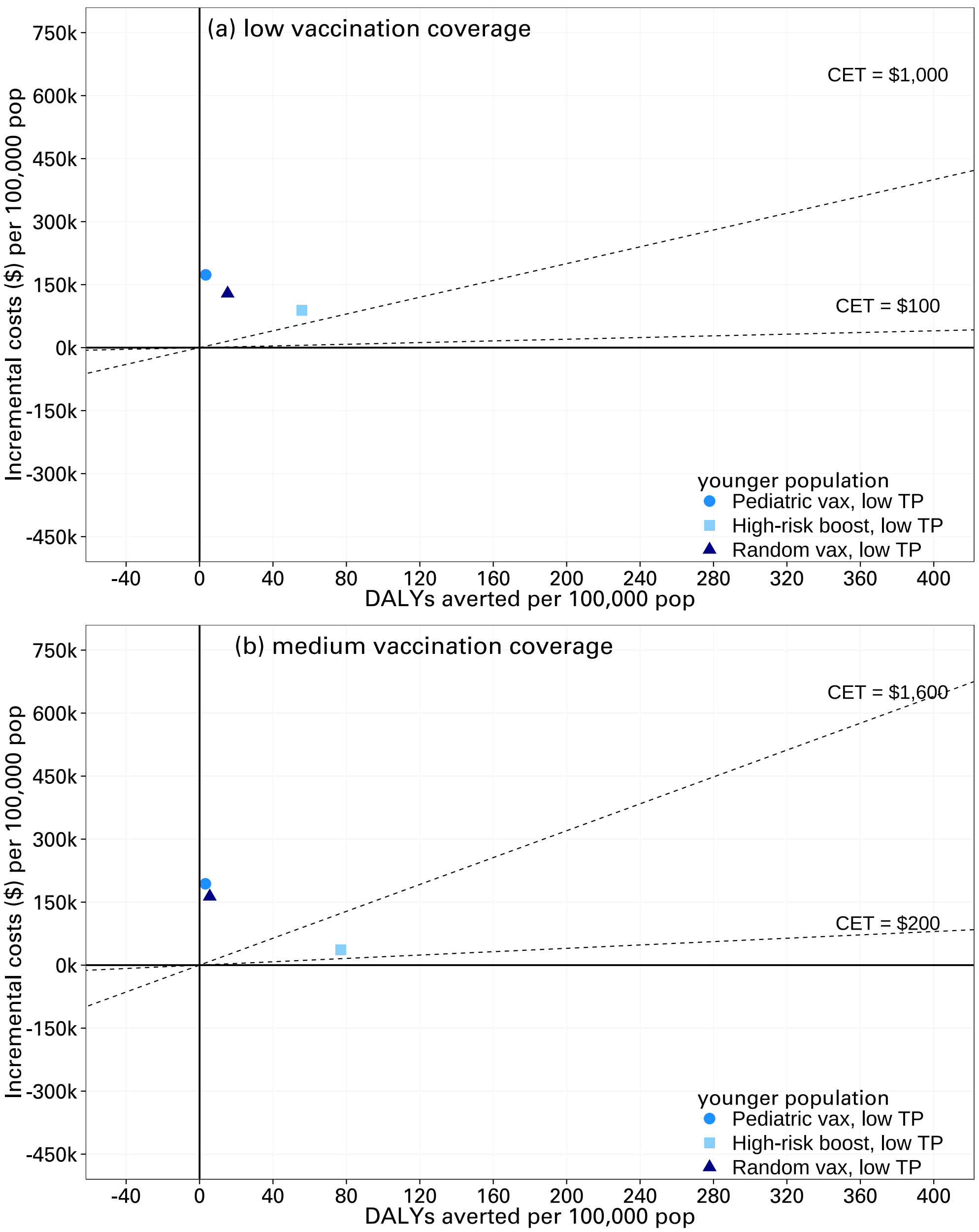

Figure E13: Cost-effectiveness analysis of vaccination in low transmission, low- and medium-vaccination coverage settings, for younger demographics. **(a)** cost-effectiveness analysis in a younger population with low vaccination coverage (initial coverage 20%); **(b)** with medium vaccination coverage (initial coverage 50%). All scenarios had an immune escape variant seeded at 2 years, with additional vaccination or boosting at 2 years. Results are for a population of 100,000 individuals.

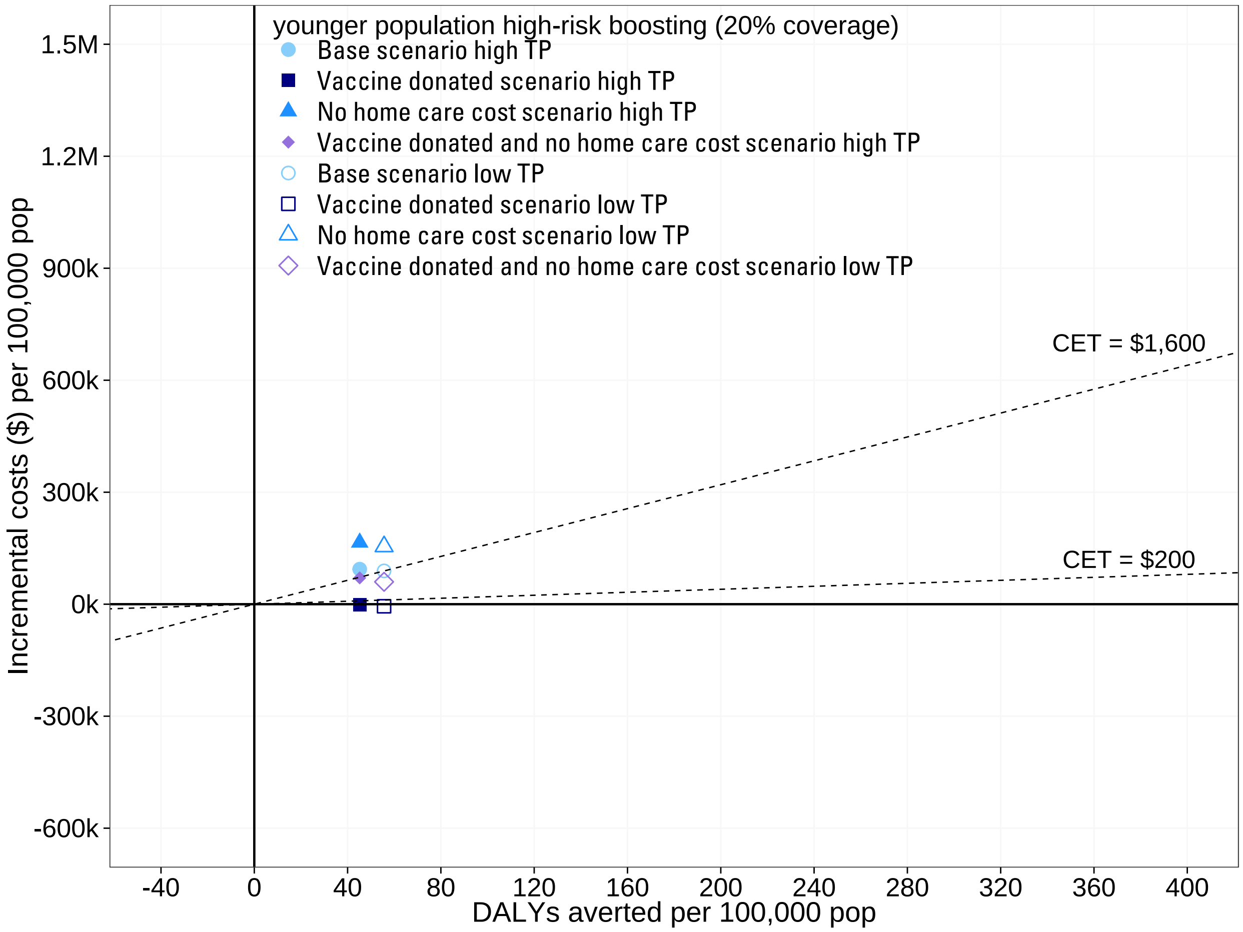

Figure E14: Cost-effectiveness analysis of high-risk boosting in the low vaccination coverage setting, for younger demographics, given different cost settings. Scenarios are run with boosting at 2 years. The dotted lines represent cost effective thresholds. Results are for a population of 100,000 individuals.

E.5 Low-medium vaccination coverage: impact of bivalent boosting - extended results

This set of scenarios (Section 3.2.2 in the main paper) considered the impact of bivalent boosting over monovalent boosting, in younger populations with low or medium vaccination coverage.

Figure E15 shows the epidemic infection curves in the high-transmission setting, while Figure E16 shows the epidemic infection curves in the low-transmission setting. Table E7 shows the median deaths in both transmission settings. We find that there is a slightly larger benefit of bivalent boosters over monovalent boosters in the low transmission potential scenario compared to the high-transmission scenarios.

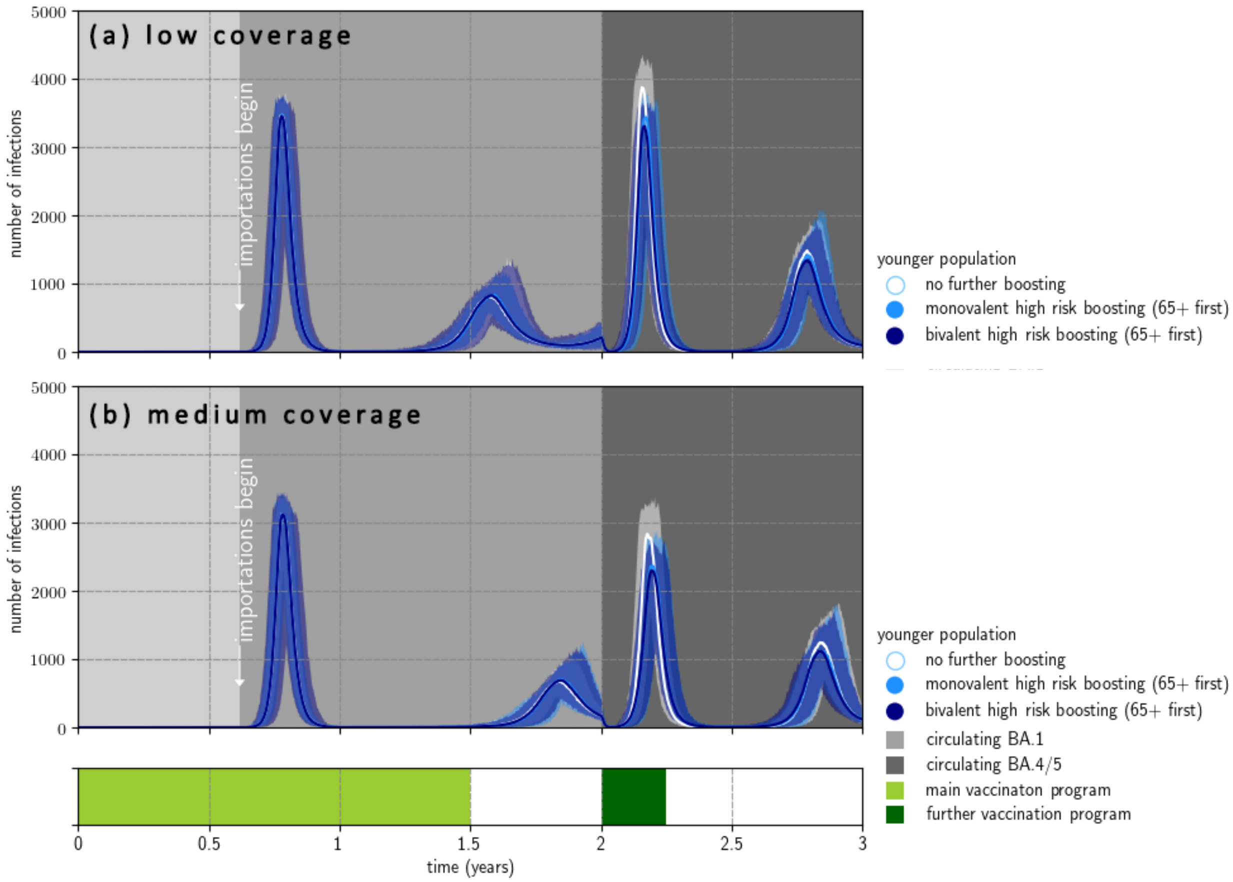

Figure E15: Outbreaks in high transmission settings with low and medium vaccination coverage in younger demographics, comparing monovalent vs bivalent boosting. **(a)** low vaccination coverage (initial coverage 20%); **(b)** medium vaccination coverage (initial coverage 50%). All scenarios had an immune escape variant seeded at 2 years, and had high-risk boosting at 2 years. The solid lines represent the pointwise median infections from 1000 simulations and the shaded regions represent the pointwise maximum and minimum infections. Results are for a population of 100,000 individuals. Bivalent high risk boosting gives a slight decrease in total numbers of infections, independent of vaccination coverage.

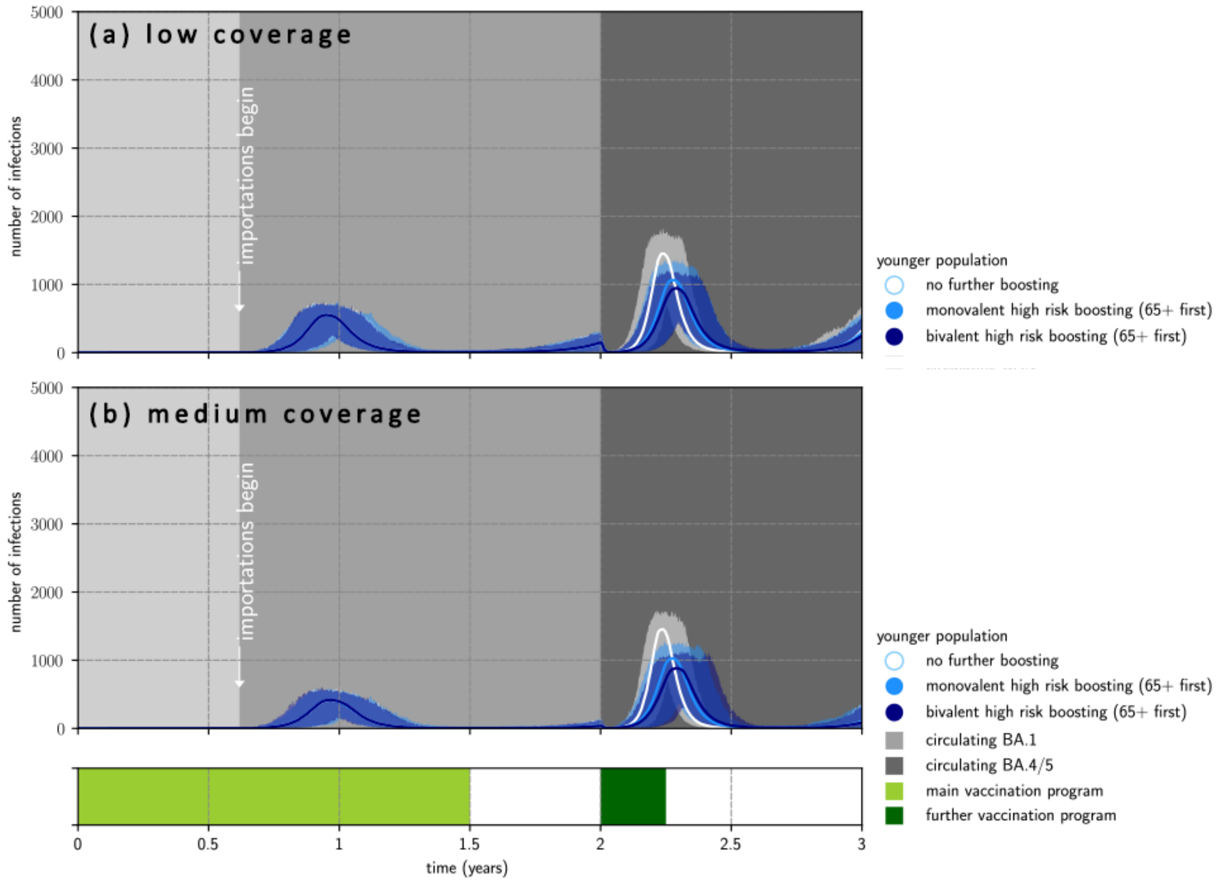

Figure E16: Outbreaks in the low transmission, low- and medium-vaccination coverage settings, for younger demographics, comparing monovalent vs bivalent boosting. (a) epidemic waves in a younger population with low vaccination coverage (initial coverage 20%); **(b)** with medium vaccination coverage (initial coverage 50%). All scenarios had an immune escape variant seeded at 2 years, with additional high-risk boosting at 2 years. The solid lines represent the pointwise median infections from 1000 simulations and the shaded regions represent the pointwise maximum and minimum infections. Results are for a population of 100,000 individuals.

Table E7: Median deaths (with 0.025 and 0.975 quantiles) between 1.5–3 years in the low and medium coverage scenarios for younger demographics, comparing high risk booster strategies using monovalent and bivalent vaccines. Each scenario has immune escape at 2·0 years, with boosting at 2.0 years. Results are for a population of 100,000 individuals.

	**Transmission potential**	**Vaccine coverage**	**Boosting strategy**	**Median deaths**
(a)	high TP	low	no further boosting	16·0 (9·0, 24·0)
	high TP	low	monovalent high-risk boosting	12·0 (5·0, 19·0)
	high TP	low	bivalent high-risk boosting	11·0 (5·0, 18·0)
(b)	high TP	medium	no further boosting	14·0 (7·0, 21·0)
	high TP	medium	monovalent high-risk boosting	10·0 (5·0, 16·0)
	high TP	medium	bivalent high-risk boosting	10·0 (4·0, 16·0)
(c)	low TP	low	no further boosting	16·0 (9·0, 24·0)
	low TP	low	monovalent high-risk boosting	11·0 (5·0, 17·0)
	low TP	low	bivalent high-risk boosting	9·0 (4·0, 16·0)
(d)	low TP	medium	no further boosting	16·0 (9·0, 25·0)
	low TP	medium	monovalent high-risk boosting	8·0 (3·0, 15·0)
	low TP	medium	bivalent high-risk boosting	7·0 (3·0, 13·0)

REFERENCES

[1] Zachreson C, Tobin R, Szanyi J, Walker C, Cromer D, Shearer FM, et al. Individual variation in vaccine immune response can produce bimodal distributions of protection. Vaccine. 2023;41(45):6630–6636. https://doi.org/10.1016/j.vaccine.2023.09.025.

[2] Khoury DS, Cromer D, Reynaldi A, Schlub TE, Wheatley AK, Juno JA, et al. Neutralizing antibody levels are highly predictive of immune protection from symptomatic SARS-CoV-2 infection. Nature Medicine. 2021 Jul;27(7):1205–1211. https://doi.org/10.1038/s41591-021-01377-8.

[3] Cromer D, Steain M, Reynaldi A, Schlub TE, Wheatley AK, Juno JA, et al. Neutralising antibody titres as predictors of protection against SARS-CoV-2 variants and the impact of boosting: a meta-analysis. The Lancet Microbe. 2022 Jan;3(1):e52–e61. https://doi.org/10.1016/S2666-5247(21)00267-6.

[4] Golding N, Ryan G, Lydeamore M.: Analyses to predict the efficacy and waning of vaccines and previous infection against transmission and clinical outcomes of SARS-CoV-2 variants. Accessed on 01/12/2022. Available from: https://github.com/goldingn/neuts2efficacy.

[5] Conway E, Walker C, Lydeamore M, Golding N, Ryan G, Mavec D, et al. Optimal timing of booster doses in a highly vaccinated population with minimal natural exposure to COVID-19. medRxiv [preprint]. 2024;https://doi.org/10.1101/2024.05.14.24307386.

[6] Hao T, Ryan GE, Lydeamore MJ, Cromer D, Wood JG, McVernon J, et al. Predicting immune protection against outcomes of infectious disease from population-level effectiveness data with application to COVID-19. Vaccine. 2025;55:126987. https://doi.org/10.1016/j.vaccine.2025.126987.

[7] Conway E, Walker C, Baker C, Lydeamore M, Ryan GE, Campbell T, et al. COVID-19 vaccine coverage targets to inform reopening plans in a low incidence setting. Proceedings of the Royal Society B: Biological Sciences. 2023;290. https://doi.org/10.1098/rspb.2023.1437.

[8] Australian Institute of Health and Welfare .: Hospitalisations and deaths following COVID-19, 2020–2022: a linked data analysis. Accessed on 03/06/2025. Available from: https://www.aihw.gov.au/reports/covid-19/hospitalisations-deaths-following-covid19-2020-22.

[9] Shearer FM, McCaw JM, Ryan G, Hao T, Tierney NJ, Lydeamore M, et al. Estimating the impact of test-trace-isolate-quarantine systems on SARS-CoV-2 transmission in Australia. Epidemics. 2024;47(100764). https://doi.org/10.1016/j.epidem.2024.100764.

[10] United Nations, Department of Economic and Social Affairs, Population Division.: World Population Prospects 2019, custom data acquired via website. Available from: https://population.un.org/wpp/DataQuery/.

[11] Willem L, Hoang TV, Funk S, Coletti P, Beutels P, Hens N. SOCRATES: an online tool leveraging a social contact data sharing initiative to assess mitigation strategies for COVID-19. BMC Research Notes. 2020 Jun;13(1). https://doi.org/10.1186/s13104-020-05136-9.

[12] Gimma A, Wong KL, Coletti P, Jarvis CI. CoMix social contact data (Poland) [Data set]. Zenodo. 2021;https://doi.org/10.5281/ZENODO.5041128.

[13] Gimma A, Wong KL, Coletti P, Jarvis CI. CoMix social contact data (Italy) [Data set]. Zenodo. 2021;https://doi.org/10.5281/ZENODO.5041112.

[14] Gimma A, Wong KL, Coletti P, Jarvis CI. CoMix social contact data (France) [Data set]. Zenodo. 2021;https://doi.org/10.5281/ZENODO.5040870.

[15] Coletti P, Wambua J, Gimma A, Willem L, Vercruysse S, Bieke Vanhoutte, et al. CoMix social contact data (Belgium) [Data set]. Zenodo. 2020;https://doi.org/10.5281/ZENODO.4035001.

[16] Melegaro A, Fava ED, Poletti P, Merler S, Nyamukapa C, Williams J, et al. Social contact data for Zimbabwe [Data set]. In PLOS ONE (Version 4). Zenodo. 2020;https://doi.org/10.5281/ZENODO.1127693.

[17] Béraud G, Kazmercziak S, Beutels P, Levy-Bruhl D, Lenne X, Mielcarek N, et al. Social contact data for France (Version 3) [Data set]. Zenodo. 2020;https://doi.org/10.5281/ZENODO.1157918.

[18] Leung K, Jit M, Lau EHY, Wu JT. Social contact data for Hong Kong [Data set]. In Scientific Reports (Version 2, Vol. 7). Zenodo. 2020;https://doi.org/10.5281/ZENODO.1165561.

[19] Horby Peter, Pham Quang Thai, Niel H, Nguyen Thi Thu Yen, Mai LQ, Thoang DD, et al. Social Contact Data For Vietnam [Data set]. Zenodo. 2018;https://doi.org/10.5281/ZENODO.1289474.

[20] Niel H, Goeyvaerts Nele, Aerts Marc, Shkedy Zib, Pierre VD, Beutels Philippe. Social contact data for Belgium (2006) (1.1) [Data set]. Zenodo. 2020;https://doi.org/10.5281/ZENODO.4059863.

[21] Lander W, Kerckhove KV, Chao Dennis L, Niel H, Beutels Philippe. Social contact data for Belgium (2010-2011) (1.2) [Data set]. Zenodo. 2020;https://doi.org/10.5281/ZENODO.4059825.

[22] Khoury DS, Docken SS, Subbarao K, Kent SJ, Davenport MP, Cromer D. Predicting the efficacy of variant-modified COVID-19 vaccine boosters. Nature Medicine. 2023 Mar;29(3):574–578. https://doi.org/10.1038/s41591-023-02228-4.

[23] Knock ES, Whittles LK, Lees JA, Perez-Guzman PN, Verity R, FitzJohn RG, et al. Key epidemiological drivers and impact of interventions in the 2020 SARS-CoV-2 epidemic in England. Science Translational Medicine. 2021;13(602):eabg4262. https://doi.org/10.1126/scitranslmed.abg4262.

[24] Commonwealth Government of Australia.: National Plan to transition Australia's National COVID-19 Response. Accessed on 01/12/2022. Available from: https://www.australia.gov.au/national-plan.

[25] Tobin RJ, Wood JG, Jayasundara D, Sara G, Walker CR, Martin GE, et al. Real-time analysis of hospital length of stay in a mixed SARS-CoV-2 Omicron and Delta epidemic in New South Wales, Australia. BMC Infectious Diseases. 2023;23. https://doi.org/10.1186/s12879-022-07971-6.

[26] Torres-Rueda S, Sweeney S, Bozzani F, Naylor NR, Baker T, Pearson C, et al. Stark choices: exploring health sector costs of policy responses to COVID-19 in low-income and middle-income countries. BMJ Global Health. 2021;6(12):e005759. https://doi.org/10.1136/bmjgh-2021-005759.

[27] World Health Organization.: Vaccine price data from public source and as reported by countries to WHO 2022. Available from: https://www.who.int/publications/m/item/c-19-vaccine-price-data-from-public-source-and-as-reported-by-countries-to-who.

[28] World Health Organization.: Analysis on COVID-19 vaccine price data from publicly available information and as reported by countries 2022. Available from: https://www.who.int/publications/m/item/analysis-on-covid-19-vaccine-price-data-from-publicly-available-information-and-as-reported-by-countries.

[29] Japanese Government.: The National Treasury's burden for the vaccination measures against COVID-19 report. Available from: https://www.mhlw.go.jp/content/000744297.pdf.

[30] Australian Government.: Budget 2021 - 2022: Budget Measures. In: Minister for Finance of the Commonwealth of Australia. Available from: https://parlinfo.aph.gov.au/parlInfo/search/display/display.w3p;query=Id%3A%22library%2Fbudget%2F2021_02%22.

[31] Yun JW, Choi MJ, Shin GS, Lim JO, Noh JY, Kim YK, et al. Cost-effectiveness of influenza vaccine strategies for the elderly in South Korea. PLoS One. 2019;14(1):e0209643. https://doi.org/10.1371/journal.pone.0209643.

[32] Xiong X, Li J, Huang B, Tam T, Hong Y, Chong KC, et al. Economic value of vaccines to address the COVID-19 pandemic in Hong Kong: a cost-effectiveness analysis. Vaccines. 2022;10(4):495. https://doi.org/10.3390/vaccines10040495.

[33] Griffiths U, Adjagba A, Attaran M, Hutubessy R, Van de Maele N, Yeung K, et al. Costs of delivering COVID-19 vaccine in 92 AMC countries. World Health Organization. 2021;8. https://www.corecommitments.unicef.org/kp/costs-of-delivering-covid19-vaccine-in-92-amc-countries.url.

[34] Griffiths U, Oyatoye I, Asman J, Mandalia N, Brenzel L, Brooks D, et al. Costs and predicted financing gap to deliver COVID-19 vaccines in 133 low-and middle-income countries. UNICEF. 2022;https://www.unicef.org/documents/costs-and-predicted-financing-gap-deliver-covid-19-vaccines-133-low-and-middle-income.

[35] Japanese Government.: 2020 Medical schedule fee points. Available from: https://www.mhlw.go.jp/stf/seisakunitsuite/bunya/0000188411_00027.html.

[36] Australian Government.: Medicare Benefits Schedule Book Operating from 21 July 2020. In: Department of Health. Available from: http://www.mbsonline.gov.au/internet/mbsonline/publishing.nsf/Content/Downloads-200701a.

[37] Hospital Authority of Hong Kong.: Hospital Authority of Hong Kong. Fees and Charges 2017. Available from: https://www.ha.org.hk/visitor/ha_visitor_index.as?Content_ID=10045&Lang=ENG&Dimension=100&Parent_ID=10044&Ver=HTML.

[38] Department of Health, Victorian Government.: Overnight stays 2022. Accessed 13 December 2022. Available from: https://www.health.vic.gov.au/patient-fees-charges/overnight-stays.

[39] Jo Y, Kim SB, Radnaabaatar M, Huh K, Yoo JH, Peck KR, et al. Model-based cost-effectiveness analysis of oral antivirals against SARS-CoV-2 in Korea. Epidemiology and Health. 2022;44. https://doi.org/10.4178/epih.e2022034.

[40] World Health Organization, et al.: WHO-CHOICE unit cost estimates for service delivery. Geneva: The Organization. Accessed 13 December 2022. Available from: https://www.who.int/teams/health-systems-governance-and-financing/economic-analysis/costing-and-technical-efficiency/quantities-and-unit-prices-(cost-inputs)/econometric-estimation-of-who-choice-country-specific-costs-for-inpatient-and-outpatient-health-service-delivery.

[41] Blakely T, Thompson J, Bablani L, Andersen P, Ouakrim DA, Carvalho N, et al. Association of Simulated COVID-19 Policy Responses for Social Restrictions and Lockdowns With Health-Adjusted Life-Years and Costs in Victoria, Australia. JAMA Health Forum. 2021 Jul;2(7):e211749. https://doi.org/10.1001/jamahealthforum.2021.1749.

[42] Global Burden of Disease Collaborative Network. Global Burden of Disease Study 2019 (GBD 2019) Disability Weights. Seattle, USA: Institute for Health Metrics and Evaluation (IHME). 2020;https://doi.org/10.6069/1W19-VX76.

[43] Singh BB, Devleesschauwer B, Khatkar MS, Lowerison M, Singh B, Dhand NK, et al. Disability-adjusted life years (DALYs) due to the direct health impact of COVID-19 in India, 2020. Scientific Reports. 2022;12(1):2454. https://doi.org/10.1038/s41598-022-06505-z.

[44] Faes C, Abrams S, Van Beckhoven D, Meyfroidt G, Vlieghe E, Hens N, et al. Time between symptom onset, hospitalisation and recovery or death: statistical analysis of Belgian COVID-19 patients. International journal of environmental research and public health. 2020;17(20):7560. https://doi.org/10.3390/ijerph17207560.

[45] Rees EM, Nightingale ES, Jafari Y, Waterlow NR, Clifford S, B Pearson CA, et al. COVID-19 length of hospital stay: a systematic review and data synthesis. BMC medicine. 2020;18:1–22. https://doi.org/10.1186/s12916-020-01726-3.

[46] Nomura S, Yamamoto Y, Yoneoka D, Haagsma JA, Salomon JA, Ueda P, et al. How do Japanese rate the severity of different diseases and injuries?—an assessment of disability weights for 231 health states by 37,318 Japanese respondents. Population Health Metrics. 2021 Apr;19(1). https://doi.org/10.1186/s12963-021-00253-4.

[47] Woods B, Revill P, Sculpher M, Claxton K. Country-level cost-effectiveness thresholds: initial estimates and the need for further research. Value in Health. 2016;19(8):929–935. https://doi.org/10.1016/j.jval.2016.02.017.

[48] Ochalek J, Lomas J, Claxton K. Estimating health opportunity costs in low-income and middle-income countries: a novel approach and evidence from cross-country data. BMJ Global Health. 2018;3(6):e000964. https://doi.org/10.1136/bmjgh-2018-000964.

[49] World Bank.: GDP per capita (current US$). Available from: https://data.worldbank.org/indicator/NY.GDP.PCAP.CD.

[50] Riedmann U, Chalupka A, Richter L, Sprenger M, Rauch W, Schenk H, et al. Estimates of SARS-CoV-2 Infections and Population Immunity After the COVID-19 Pandemic in Austria: Analysis of National Wastewater Data. The Journal of Infectious Diseases. 2025 02;231(5):e921–e928. https://doi.org/10.1093/infdis/jiaf054. https://academic.oup.com/jid/article-pdf/231/5/e921/61857786/jiaf054.pdf.

[51] Le TP, Abell I, Conway E, Campbell PT, Hogan AB, Lydeamore MJ, et al. Modelling the impact of hybrid immunity on future COVID-19 epidemic waves. BMC Infect Dis. 2024;(24):407. https://doi.org/10.1186/s12879-024-09282-4.

[52] Eales O, Teo M, Price DJ, Hao T, Ryan GE, Senior KL, et al. Temporal trends in test-seeking behaviour during the COVID-19 pandemic. medRxiv. 2024;https://doi.org/10.1101/2024.06.06.24308566. https://www.medrxiv.org/content/early/2024/06/07/2024.06.06.24308566.full.pdf.
